# Threefold-Hierarchical Transport of Highly Concentrated Aqueous Electrolyte Mediated by Environment-Reconstructed Ion Correlation Networks

**DOI:** 10.1007/s40820-026-02075-1

**Published:** 2026-02-03

**Authors:** Qiang Wang, Di Tian, Zhiguo Qu

**Affiliations:** MOE Key Laboratory of Thermal-Fluid Science and Engineering, School of Energy and Power Engineering, Xi’an Jiaotong University, Xi’an, 710049 People’s Republic of China

**Keywords:** Ion–ion correlation, Hydrogen bond network connectivity, Interfacial water structure, Heterogeneous aggregation, Nanoconfinement

## Abstract

Highly concentrated aqueous electrolytes (HCAEs) offer superior energy density and stability in energy conversion and storage than their diluted counterparts, attributed to enhanced ion transport and correlated ion structures. However, their underlying structure–transport relationships remain poorly understood in wide-temperature and nanoconfinement environments. This study captures electrolyte structure and transport fingerprints shaped by environmental factors, by combining experimental characterization with first-principles molecular simulations at sub-nanometer resolution. It is revealed that ultrahigh concentration changes electrolyte electronic states and forms ion correlation networks with extensive aggregates. These alterations reduce free water content and hydrogen bond network connectivity, resulting in notable deviation from the Nernst–Einstein (NE)-predicted conductivity. This deviation is thermal-alleviated by weakening ion correlations. Nanoconfined interfaces create oscillatory-decaying distribution and heterogeneous orientation in HCAE constituents, resulting in redrawn ion correlation networks and localized NE deviations. Such transport behaviors are further modulated by synergistic thermal-interfacial constraints. Taking NE deviations as descriptors, HCAE transport, mediated by environment-reconstructed ion correlation networks, is then summarized to present threefold-hierarchical variations due to ion concentration, thermal effect, and confinement extent. This threefold-hierarchical framework is transferable among diverse electrolytes, offering a localized insight for electrolyte evaluation in electrochemical energy devices.

**Supplementary Information:**

The online version contains supplementary material available at 10.1007/s40820-026-02075-1.

## Highlights

- Transport fingerprints of aqueous electrolytes are captured to be mediated by environment-reconstructed ion correlation networks.
- Taking the Nernst–Einstein deviations as descriptors, electrolyte transport presents threefold-hierarchical variations due to salt concentration, thermal effect, and nanoconfined interface.
- This threefold-hierarchical framework is transferable among diverse electrolytes, offering a localized insight for electrolyte evaluation in electrochemical energy devices.

**Supplementary Information:**

The online version contains supplementary material available at 10.1007/s40820-026-02075-1.

## Graphical abstract

**Figure:**
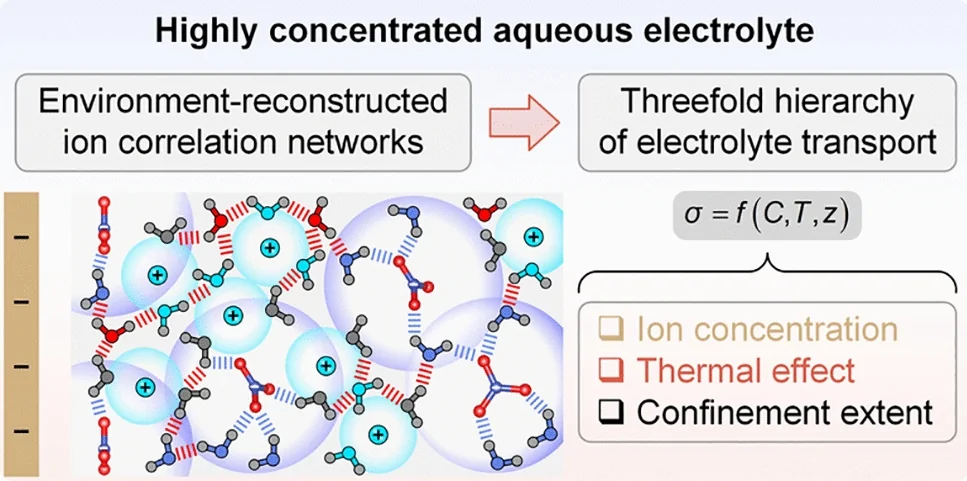

## Introduction

Ions serve as basic carriers in energy conversion and storage, and the application scenarios of electrolytes are extensible by altering ion charge density. Diluted aqueous electrolytes (DAEs) are widely used in ion separation [1, 2], nanogenerators [3, 4], and ion sensing [5]. By contrast, highly concentrated aqueous electrolytes (HCAEs), in which salt content exceeds that of solvents by weight and volume, have unique occurrence states of ions and solvents. They are promising candidates in ion batteries [6, 7], supercapacitors [8, 9], and electrocatalysis [10] due to high energy density and electrochemical stability. Precise evaluation of HCAE structure–transport relationship is vital for electrolyte design in electrochemical energy devices.

HCAE transport behaviors are primarily determined by ion concentration, thermal effect, and nanoconfined interface in applications. Ultrahigh concentration is basically responsible for HCAE transport fingerprints. From an ion insight, ion–ion correlations produce ion clusters and charge aggregates, inducing dense networks and collective ion motion. Distinct from solvent-free ionic liquids [11], this collective motion is more complex in HCAEs due to the participation of water solvent. For instance, neutral aggregates consisting of equal cations and anions do not contribute to electrolyte conductivity, and negative clusters make opposite contributions due to the dragged cations by anions [12, 13]. From a solvent insight, the less free-state water in HCAEs reduces solvent activity in lithium batteries [14, 15] and zinc batteries [16, 17]. Water can even reshape the aggregate composition, so that it no longer merely offers background resistance for ion transport. Given these complexities, the classical Nernst–Einstein (NE) relation presents notable deviations for evaluating HCAE transport [18, 19], despite its achievements in DAE-based scenarios such as desalination [20], water treatment [21], and ion logic devices [22]. This deviation stems from the fact that the NE relation neglects ion–ion-correlated motions and treats all ions equally, without distinguishing whether they belong to neutral or charged species. Hence, the intricate cation–anion–solvent interplay and water states still require exploration, thereby offering a bottom-up understanding of the structure–transport relationship of HCAEs.

The deviation from the NE relation in HCAE-based devices is further modulated by wide temperature ranges. For instance, the non-freezable bound-state solvents endow aqueous ion batteries and capacitors with robust low-temperature function [23–26], and the ultrahigh ion density enhances the power output of osmotic energy conversion devices in high-salinity and high-temperature situations [27]. These diverse scenarios imply a thermal-regulated equilibrium of dynamic formation and dissociation of ion aggregates, thereby altering the concentration of effective charge carriers. Temperature is thus a regulator of HCAE microstructures, including ion correlation and hydrogen bond (H-bond) networks, no longer merely an accelerator of species transport. This electrolyte transport behavior, determined by thermal-induced structure evolution, clearly deviates from the NE assumption that ions are independent charge carriers. Hence, the structure–transport relationship and the NE deviation of HCAEs require decoding under thermal regulations, aiming at the accurate prediction of device outputs.

HCAE microstructure and its NE deviation are even mysterious at nanoconfined interfaces, such as electrode pores in aqueous batteries [28], catalyst surfaces in CO_2_ electroreduction [29], and ion-selective channels in osmotic power generation [30, 31]. These interfacial scenarios have heterogeneous local environments, leading to position-dependent aggregation and orientation of species. HCAE structure and the NE deviation are thus expected to present spatial heterogeneity. However, the NE relation typically treats ion motion in a homogeneous medium when discussing the electrolyte transport, so the entire electrolyte space is roughly simplified to a single package. In fact, as preliminarily explored by spectroscopic techniques [32–34], sub-nanometer-resolution explorations are necessary to probe species occurrence states, thereby unraveling the interface-reshaped NE deviation for HCAE transport.

In this study, the visualized and quantified explorations of bulk and interfacial aqueous electrolytes are performed by integrating infrared (IR) and Raman characterization, pulsed-field gradient nuclear magnetic resonance (PFG-NMR) experiment, conductivity measurement, and first-principles molecular dynamics (FPMD) simulation. The origin and expression of ion correlation networks are clarified to reveal the environment-controlled HCAE transport. Taking NE deviations as descriptors, threefold-hierarchical variations in HCAE transport features are then summarized to revisit its behavior at sub-nanometer resolution. This hierarchical framework provides a localized insight for electrolyte evaluation in electrochemical applications.

## Experimental Section

### Experimental Characterization of Bulk Electrolyte

Due to the low cost and excellent conductivity, NaNO_3_ aqueous solutions serve as promising alternatives in energy conversion and storage. They are thus selected to evaluate the structure–transport relationship of aqueous electrolytes. In detail, NaNO_3_ salt is dissolved in distilled water to prepare electrolytes at 0.01, 0.71, and 7.29 M, representing DAE, moderately concentrated aqueous electrolytes (MCAE), and HCAE, respectively. MCAE is a transited concentration between the other two values. These concentrations are chosen by balancing the rationality of experimental conditions with the accessibility of subsequent FPMD simulations, thereby probing the concentration dependence of structure–transport association. The concentration of 0.01 M is generally a threshold of ideal diluted solutions. The concentration of 7.29 M meets the HCAE definition and approaches NaNO_3_ solubility limit. The concentration of 0.71 M shows an order difference from DAE and HCAE concentrations, and a lower value may cause fewer ions and inadequate sampling in simulations.

To probe the electrolyte structures, IR and Raman spectra are characterized for bulk DAE, MCAE, and HCAE under various temperature conditions. To probe the solvent water properties with concentration dependence, PFG-NMR experiments are then conducted at room temperature to obtain two-dimensional (2D) diffusion-ordered spectroscopy (DOSY) of ^1^H nuclei. Additional experiments are performed for HCAE at 274, 285, 315, and 330 K, to evaluate the temperature-dependent water transport. This range can avoid low-temperature freezing and high-temperature irreversible changes of HCAEs. The NMR signal is related to water diffusivity *D*_w_, and *D*_w_ values are fitted to extract the activation energy for water transport (*E*_a,w_) in HCAEs.

After clarifying the water behaviors, temperature-dependent conductivity measurements are performed for the three concentrations across a temperature range (270, 285, 300, 315, and 330 K). The tests are conducted at constant temperature (humidity: 20%) through the four-electrode method using a DZB-712F multiparameter analyzer (REX, INESA Scientific Instrument Co., Ltd.). Before testing, the instrument undergoes rigorous calibration using standard solutions to guarantee measurement accuracy. To ensure temperature uniformity, a stabilization period of 10–15 min is conducted after each temperature adjustment before data collection. During data collection, temperature and its relative deviation are monitored through integrated sensor arrays. All tests are repeated three times. To assess the concentration dependence of activation energy for electrolyte transport *E*_a,e_, the measured conductivity *σ*_exp_ is fitted using the Arrhenius equation. The definitions of *D*_w_, *E*_a,w_, *E*_a,e_, and *σ*_exp_ are shown in Note S1.

### FPMD Simulation of Bulk Electrolyte

To ensure consistency of concentration and temperature with experiments, bulk electrolyte models at 0.71 and 7.29 M are established for FPMD simulations, representing the MCAE and HCAE, respectively. DAE is not included in simulations for two reasons: Experimental results here indicate minor differences in solution environments between DAE and MCAE, and the limited model size makes it infeasible to reproduce concentrations low to 0.01 M. Hence, MCAE contains 3 Na⁺/NO_3_^−^ pairs and 222 water molecules (simulated at 300 K), and HCAE contains 31 Na⁺/NO_3_^−^ pairs and 143 water molecules (at 270, 300, and 330 K). Both cubic models show dimensions of 19.19 × 19.19 × 19.19 Å^3^. To minimize the equilibration time, classical MD simulations are performed to extract final snapshots as initial structures for FPMD simulations. FPMD simulations of these bulk models are conducted using the NVT ensemble. Computational details are shown in Note S2 and Table S1.

To uncover the electrolyte behaviors in diverse environments, this study analyzes physical quantities in four aspects, including electronic, structural, and thermodynamic properties, as well as transport performance. Electronic properties include electrostatic potential (ESP) distribution and projected density of states (PDOS). Structural properties involve the spatial distribution of species, radial distribution function (RDF, also written as *g*(*r*)), radial space charge *ρ*_*e*_(*r*), spatial distribution function (SDF), H-bond network, and water occurrence state. Specifically, H-bonds are classified into water–water and anion–water ones, denoted as HB_w-w_ and HB_A-w_, respectively, and the normalized H-bond number is calculated. Water occurrence states are divided into four types: not solvated by any ion (H_2_O_F_), solely solvated by cations (H_2_O_C_), solely solvated by anions (H_2_O_A_), and co-solvated by cations and anions (H_2_O_CA_).

The dissociation energy barrier Δ*G* of ion pairs is calculated to assess ion–ion correlation strength from a thermodynamic insight. Transport performance is assessed by water diffusivity (*D*_w_) and electrolyte conductivity. For a binary 1:1 electrolyte, the NE relation links electrolyte conductivity ${\sigma }_{\mathrm{NE}}$ with ion diffusivity:

1 $$\sigma_{{{\mathrm{NE}}}} = \sigma_{ + }^{{\mathrm{s}}} + \sigma_{ - }^{{\mathrm{s}}} = \frac{{F^{2} }}{RT}\left( {c_{ + } D_{ + } + c_{ - } D_{ - } } \right)$$

where $\sigma_{ + }^{{\mathrm{s}}}$ and $\sigma_{ - }^{{\mathrm{s}}}$ are cation and anion self-diffusion contributions, respectively. *c* and *D* are ion concentration and diffusivity, respectively. Subscripts + and − denote cation and anion, respectively. *F*, *R*, and *T* are the Faraday constant, gas constant, and temperature, respectively.

In fact, electrolyte conductivity is accurately a sum of self-diffusion and ion–ion-correlated contributions, as expressed by the Green–Kubo (GK) relation:

2 $$\sigma_{{{\mathrm{GK}}}} = \sigma_{ + }^{{\mathrm{s}}} + \sigma_{ - }^{{\mathrm{s}}} + \sigma_{ + + }^{{\mathrm{d}}} + \sigma_{ - - }^{{\mathrm{d}}} - 2\sigma_{ + - }^{{\mathrm{d}}}$$

In this expression, in addition to the self-diffusion contributions ($\sigma_{ + }^{{\mathrm{s}}}$ and $\sigma_{ - }^{{\mathrm{s}}}$), $\sigma_{ + + }^{{\mathrm{d}}}$, $\sigma_{ - - }^{{\mathrm{d}}}$, and $\sigma_{ + - }^{{\mathrm{d}}}$ denote the partial conductivity contributions arising from cation–cation, anion–anion, and cation–anion correlations, respectively. The factor of 2 before $\sigma_{ + - }^{{\mathrm{d}}}$ is derived from rigorous mathematical calculations, so it is generalized and independent of charges carried by cations and anions. Ion correlation networks thus result in deviations from the NE relation, depending on the ion–ion correlation strength. Two dimensionless quantities are then defined to quantify the NE deviation, including the Haven ratio *H*_*R*_ and cation transference number *t*_+_. *H*_*R*_ is defined as:

3 $$H_{R} = \frac{{\sigma_{{{\mathrm{NE}}}} }}{{\sigma_{{{\mathrm{GK}}}} }} = \frac{{\sigma_{ + }^{{\mathrm{s}}} + \sigma_{ - }^{{\mathrm{s}}} }}{{\sigma_{ + }^{{\mathrm{s}}} + \sigma_{ - }^{{\mathrm{s}}} + \sigma_{ + + }^{{\mathrm{d}}} + \sigma_{ - - }^{{\mathrm{d}}} - 2\sigma_{ + - }^{{\mathrm{d}}} }}$$

*t*_+_ is defined as the relative contribution of cation motion to $\sigma_{{{\mathrm{GK}}}}$:

4 $$t_{ + } = \frac{{\sigma_{ + }^{{\mathrm{s}}} + \sigma_{ + + }^{{\mathrm{d}}} - \sigma_{ + - }^{{\mathrm{d}}} }}{{\sigma_{ + }^{{\mathrm{s}}} + \sigma_{ - }^{{\mathrm{s}}} + \sigma_{ + + }^{{\mathrm{d}}} + \sigma_{ - - }^{{\mathrm{d}}} - 2\sigma_{ + - }^{{\mathrm{d}}} }}$$

Ion correlations are negligible in low-concentration electrolytes, so *H*_*R*_ values are close to one. However, in HCAEs and nanoconfinement scenarios, strong ion correlations and heterogeneous aggregates lead to *H*_*R*_ deviating from one and *t*_+_ with negative values. In this case, the transport behavior deviates from the NE relation. Details are shown in Note S1.

### FPMD Simulation of HCAE at Interfaces

HCAEs and carbon-based materials form a mature combination in electrochemical energy devices, and graphene is a representative carbon material [35]. Hence, this study confines a 7.29 M NaNO_3_ solution between two graphene sheets, forming two symmetrical graphene–HCAE interfaces for FPMD simulations. The lateral size in the *xy* plane of such an orthorhombic cell is 17.22 × 17.04 Å^2^. Its vertical dimension in the *z*-direction is 39 Å, which includes a 12 Å vacuum layer to minimize the periodic image interactions. FPMD simulations of these interfacial models are performed at 270, 300, and 330 K using the NVT ensemble. Detailed parameters are shown in Note S2.

To investigate HCAE behaviors at interfaces, the physical quantities involve electronic and structural properties as well as transport performance. Electronic property involves the electronic density difference ${\Delta }\rho_{{\mathrm{e}}}$ and Bader charge. Structural property includes species number density $\rho_{n}$ (e.g., ions, water molecules, and H-bonds), as well as anion and water orientations. Anion orientation at interfaces is assessed by a distance–angle colormap and orientation preference *P*_NP_ of anion plane. Water orientation is assessed by another distance–angle colormap and orientation preference *P*_wd_ of water dipole.

Moreover, the approach established for bulk electrolytes is followed to assess the position-dependent HCAE transport with sufficient statistical sampling. In detail, the partial conductivity, *H*_*R*_, and *t*_+_ are calculated separately in regions I, II, and III. System parameters (e.g., volume *V*, number of ions *N*_+_ and *N*_–_, and ion concentration) are adjusted according to the size and number density of each region. All structural properties and transport performance in interfacial systems are averaged over two symmetric interfaces. Details are shown in Note S1.

## Results and Discussion

### Comparison of DAE and HCAE

**Fig. 1:**
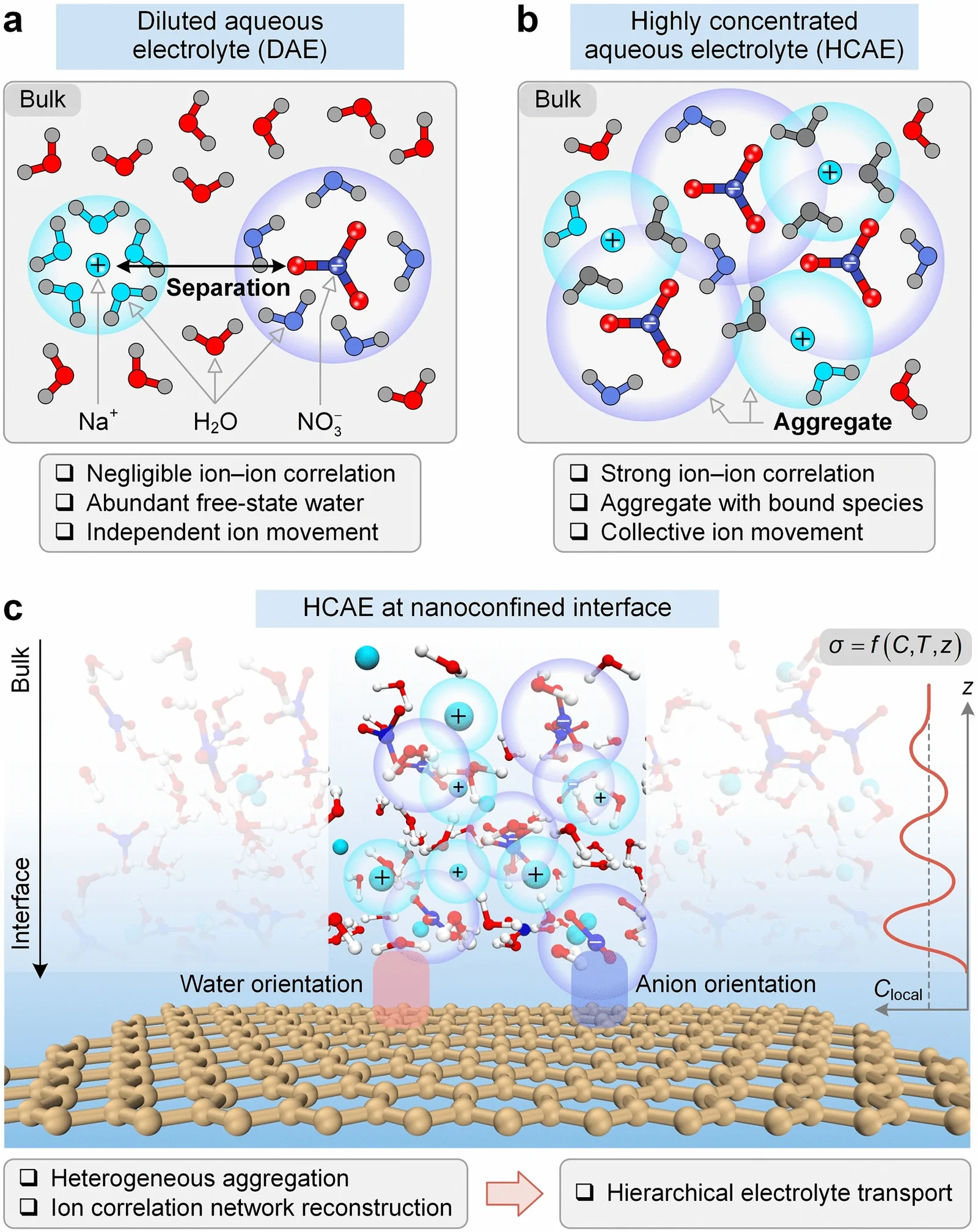
Comparison of DAE and HCAE. **a** and **b** DAE and HCAE in bulk states, respectively. **c** HCAE at nanoconfined interface

The transport behaviors of aqueous electrolytes rely on environments of ion concentration, temperature, and nanoconfinement. Figure 1 compares DAE and HCAE. The low-cost and low-concentration sodium nitrate (NaNO_3_) widely appears in desalination [36] and osmotic energy conversion [37]. It even has low viscosity and high conductivity at high concentration, serving as a promising alternative for aqueous energy storage [38, 39]. Hence, this study employs NaNO_3_ aqueous electrolyte to explore the intrinsic association between electrolyte microstructures and transport behaviors.

In terms of bulk DAE (Fig. 1a), ion–ion correlations are negligible due to solvent-separated ions and abundant free-state water, and ion presents independent movement without mutual interferences. The electrolyte transport thus conforms to the NE relation. In terms of bulk HCAEs (Fig. 1b), the crowded environment leads to overlapping hydration shells between neighboring ions and induces strong ion correlations. Ion correlation networks then produce aggregates with bound species and trigger the collective ion movement.

When HCAEs appear at the nanoconfined interface (Fig. 1c), in addition to solvation effects and ion correlations, the pattern and composition of aggregates also exhibit spatial heterogeneity, due to the disparate confinement levels at different distances from the surface. For instance, the local constituent concentration presents an oscillatory decay with an attenuated confinement level, and ion and water nearest to the surface adopt their preferred orientations. These fingerprints endow HCAEs with localized ion correlation networks and spatially hierarchical transport. Thermal effects further perturb the aggregate stability and reshape ion correlation at interfaces. Hence, electrolyte conductivity and its partial contributions must be described by complex functions of local ion concentration, temperature, and spatial position within interfacial regions. It is crucial to reveal the environment-controlled ion correlation networks and HCAE transport behaviors for electrolyte evaluation in energy conversion and storage.

### Research Framework

**Fig. 2:**
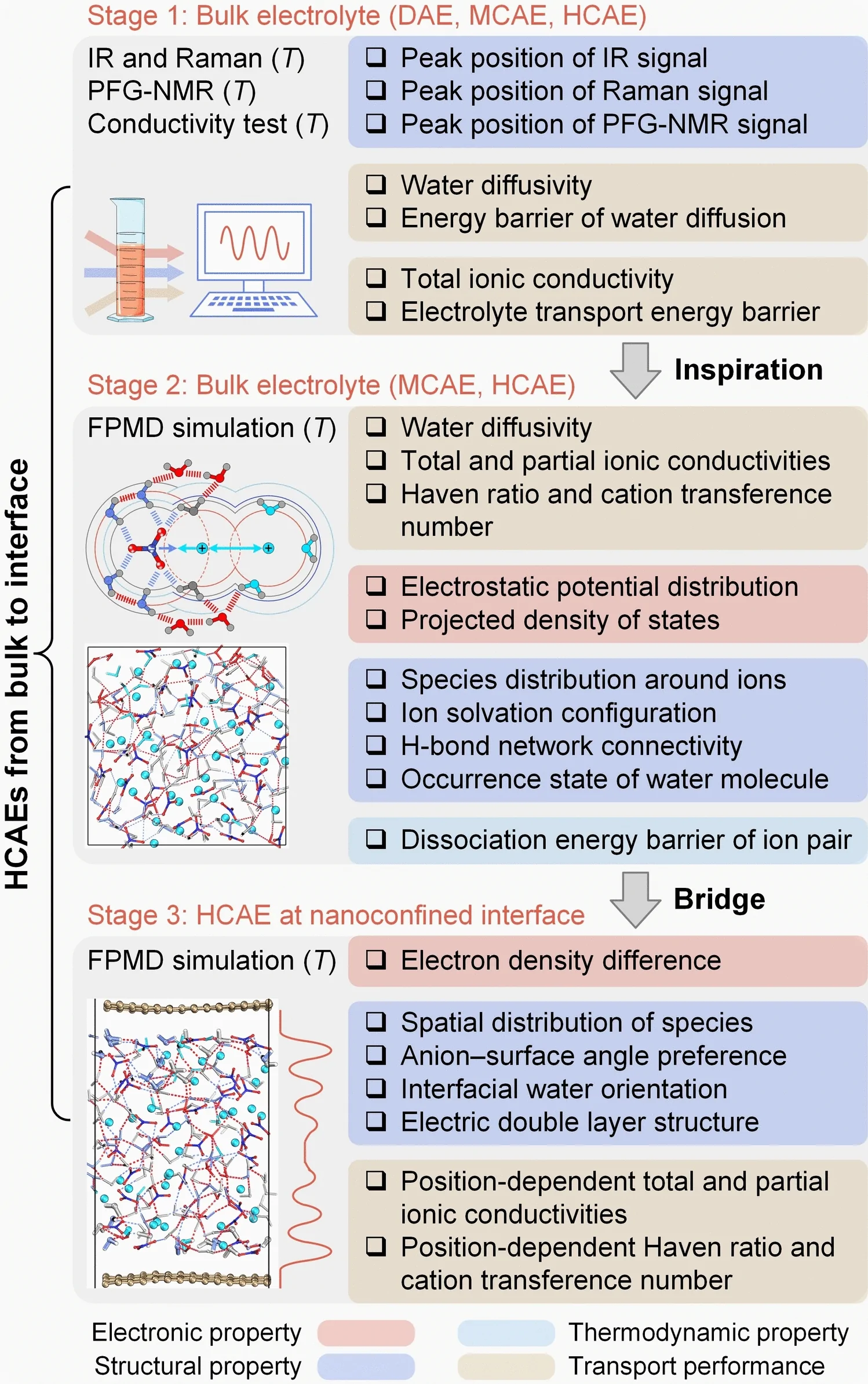
Research framework in this study

To probe the environment-shaped HCAE behaviors from bulk to interface, Fig. 2 shows the three-stage research framework. By integrating experimental characterizations with first-principles simulations, multiple environmental factors are discussed, including ion concentration, thermal effect, nanoconfined interface, and the thermal-interfacial coupling. The first stage probes the structure–transport relationship of bulk electrolytes under three concentration regimes, including DAEs, HCAEs, and MCAEs. IR and Raman spectra are employed to clarify the environment-dependent structural features. Given the critical water role in electrolyte transport, PFG-NMR is used to evaluate water structure and transport with concentration and temperature dependences. The conductivity is then measured to quantify the transport performance. Hence, the structure metrics include peak positions of IR, Raman, and NMR signals, and the transport metrics involve water diffusivity and its energy barrier, as well as electrolyte conductivity and its concentration-controlled energy barrier.

Electrolyte transport performance can be traced back to the intrinsic features of constituents. Inspired by the divergent structural and transport behaviors in experiments, the second stage performs FPMD simulations for bulk MCAE and HCAE. Such arrangements aim to quantify the contributions of ion correlations to electrolyte transport. The outputs involve water diffusivity as well as total and partial ionic conductivities. Meanwhile, *H*_*R*_ and *t*_+_ are descriptors to quantify the deviation from the NE relation-predicted conductivity. The second stage further probes the origins of environment-dependent transport from the insight of ion correlation networks. The outputs involve three aspects: electronic, structural, and thermodynamic properties. Electronic properties are the ESP distributions of isolated components and the PDOS of electrolyte systems. Structural properties involve the species distribution around ions, ion solvation structure, H-bond network connectivity, and water occurrence state. A thermodynamic property is the dissociation energy barrier of ion pairs.

HCAEs commonly appear at nanoconfined interfaces in applications, where experimental techniques suffer from limited spatial resolution for the distinct confinement levels. To address this challenge, the third stage focuses on interfacial HCAEs by performing room-temperature FPMD simulations, extending the fundamental insights obtained from bulk states to interfaces. This stage highlights the role of solid surfaces in reconstructing ion correlation networks and transport behaviors of HCAEs. Temperature-regulated FPMD simulations are further conducted to explore the electrolyte behaviors under the coupled impacts of nanoconfinement and thermal effect. The interface-specific outputs include electron density difference, spatial distribution of species, anion–surface angle preference, interfacial water orientation, and electric double layer (EDL) structure. Position-dependent HCAE transport at interfaces is then quantified by total and partial ionic conductivities, *H*_*R*_, and *t*_+_.

The above discussions link the electronic, structural, and thermodynamic properties with the transport performance. Subsequently, the NE deviation serves as a descriptor to summarize HCAE transport behaviors mediated by environment-reconstructed ion correlation networks, offering a reliable evaluation pathway for aqueous electrolytes in electrochemical devices.

### Spectroscopic Fingerprint and Transport Performance of Bulk Electrolyte

**Fig. 3:**
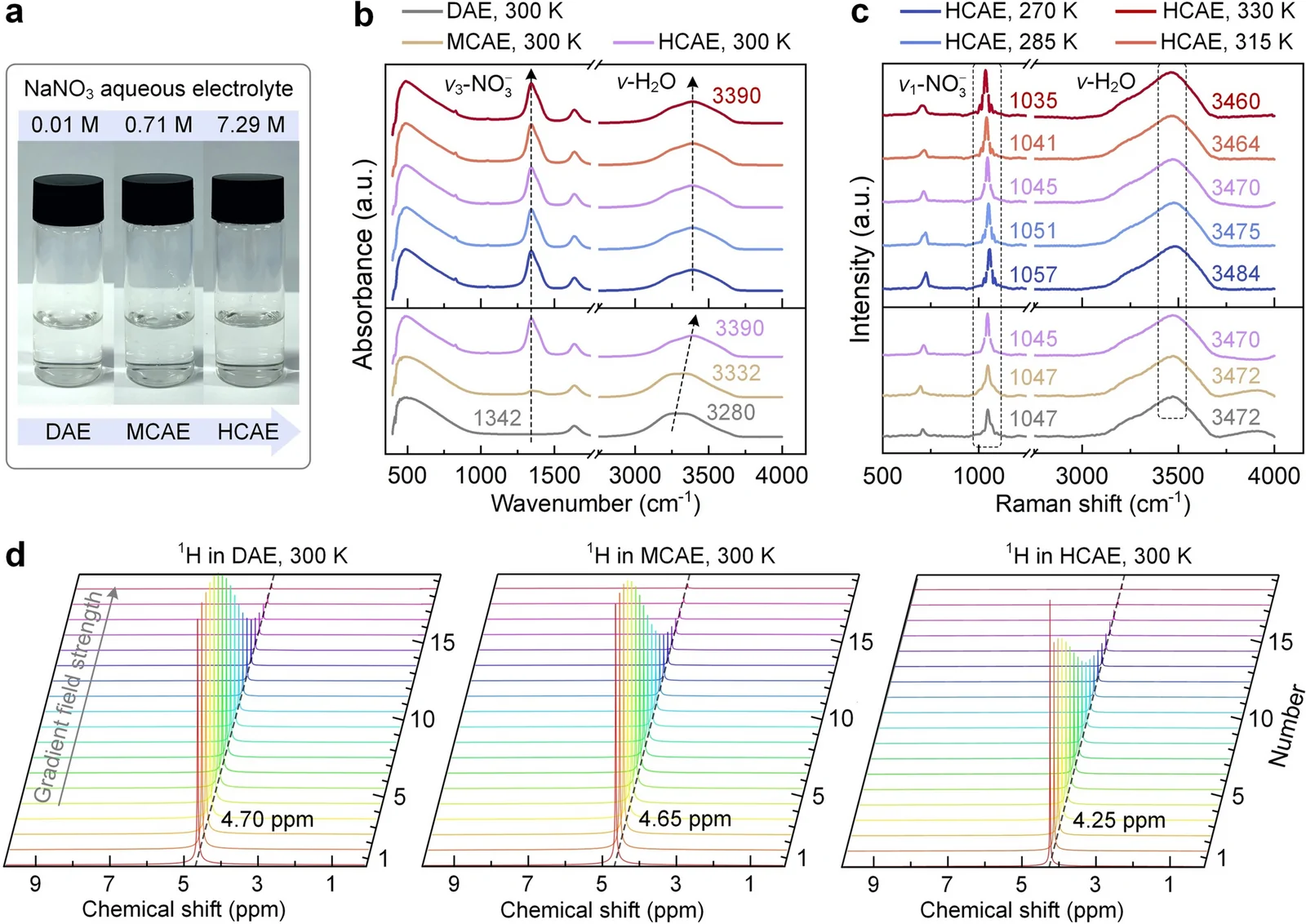
Environment-dependent spectroscopic fingerprint of bulk aqueous electrolytes. **a** Aqueous electrolyte samples used in experiments. **b** and **c** IR and Raman spectra of bulk aqueous electrolytes, respectively. Panels **b** and **c** share the same legend. IR spectra in panel (**b**) are normalized to the intensity of the band near 490 cm^−1^. Raman spectra in panel (**c**) are normalized to the *v*-H_2_O band intensity, and the *v*_1_-NO_3_^−^ band intensity in HCAEs is reduced to one-fifth of its normalized value for clarity. **d** Room-temperature PFG-NMR spectrum of ^1^H nucleus in DAE, MCAE, and HCAE, respectively

This study starts from experimental characterization for bulk electrolytes. Figure 3a shows NaNO_3_ aqueous electrolyte samples used for characterizations, covering DAE (0.01 M), MCAE (0.71 M), and HCAE (7.29 M). Figure 3b presents the environment-dependent IR spectra of the samples. The peak at 1342 cm^−1^ is the antisymmetric stretch in NO_3_^−^ ions (denoted as *v*_3_-NO_3_^−^), and the peak at 3000–3600 cm^−1^ indicates water O–H stretch (denoted as *v*-H_2_O). As an increased concentration from DAE to MCAE and HCAE at 300 K, the absorption peak of *v*_3_-NO_3_^−^ orderly evolves from negligible to remarkable. This result stems from the fact that water molecules fail to fully shield ion–ion interactions at an ultrahigh concentration, and strong ion–ion correlation places anions in a highly asymmetric local environment. Meanwhile, the absorption peak of *v*-H_2_O shifts from 3280 to 3332 and 3390 cm^−1^ with an enlarged concentration. This concentration-dependent shift stems from the impaired role of strong HB_w–w_ and the enhanced role of weak HB_A–w_ without fully breaking H-bond networks. This alteration is confirmed by the decomposed *v*-H_2_O spectra (Fig. S1), inspired by previous reports [26, 40].

Figure 3c displays the environment-dependent Raman spectra of the samples. The peak at 1030–1060 cm^−1^ indicates the symmetric N–O stretch in NO_3_^−^ ions (denoted as *v*_1_-NO_3_^−^). As the concentration increases from DAE to MCAE at 300 K, the peaks of *v*_1_-NO_3_^−^ and *v*-H_2_O remain at 1047 and 3472 cm^−1^, respectively, implying a minor difference in the electrolyte environment between DAE and MCAE. Upon increasing the concentration to HCAE, the peaks of *v*_1_-NO_3_^−^ and *v*-H_2_O shift to 1045 and 3470 cm^−1^, respectively. This result stems from that the ultrahigh concentration weakens anion N–O bonds by enhancing cation–anion interactions, and it impedes water O–H bonds by forming the crowded aggregates. In terms of temperature-affected HCAEs, the *v*_1_-NO_3_^−^ peak shifts from 1057 to 1035 cm^−1^ with temperature from 270 to 330 K, indicating the thermal-weakened anion N–O bonds. Meanwhile, the *v*-H_2_O peak shifts from 3484 to 3460 cm^−1^. This specific shift, deviating from the pattern in pure water and diluted electrolytes, is reasonable in HCAEs, as previously reported [26]. Intrinsically, the *v*-H_2_O peak is co-shaped by multiple factors, such as anion hydrophilicity and species microstructure. Thermal effects alter H-bond strength, relax the electrolyte structure, and rearrange ion–water coordination in HCAEs simultaneously. The eventual output mimics a decreased concentration (still belonging to the HCAE), shifting the *v*-H_2_O peak to lower frequencies. Ion concentration and thermal effects thus reshape ion–ion and ion–water interactions in aqueous electrolytes.

Note that the *v*-H_2_O peak appears at different locations in IR and Raman spectra, and it shifts much more in IR than in Raman results with a transition from DAE to MCAE and HCAE. This discrepancy arises since the two techniques probe different aspects of the water O–H stretch. In IR spectra, the water O–H stretch is highly sensitive to variations in dipole moment, which is strongly affected by H-bond strength. From DAE to MCAE and HCAE, the strong HB_w–w_ are progressively replaced with weak HB_A–w_, leading to a pronounced blue shift of *v*-H_2_O. In contrast, Raman scattering is governed by changes in molecular polarizability. The *v*-H_2_O band in Raman spectra represents a collective vibration averaged over diverse water species, including weakly H-bonded and non-H-bonded OH groups. Hence, its peak position exhibits only a small shift with ion concentration, consistent with the previous report [41].

Figure 3d shows the ^1^H PFG-NMR spectrum at 300 K. The ^1^H peaks of DAE and MCAE are located at 4.70 and 4.65 ppm (Fig. S2), respectively, implying a minor difference in the solution environments. The ^1^H peak negatively shifts to 4.25 ppm in HCAE, due to the reduced electron cloud density around H nuclei, which stems from the enhanced H-bond deshielding effect due to more HB_A–w_ in HCAEs. As shown in Fig. S3, the ^1^H peak in HCAE positively moves from 4.10 to 4.40 ppm as temperature rises from 274 to 330 K. This result is ascribed to the enhanced electron cloud density around H nuclei, which is caused by the reduced H-bond deshielding effect due to fewer HB_A-w_ under thermal effects.

**Fig. 4:**
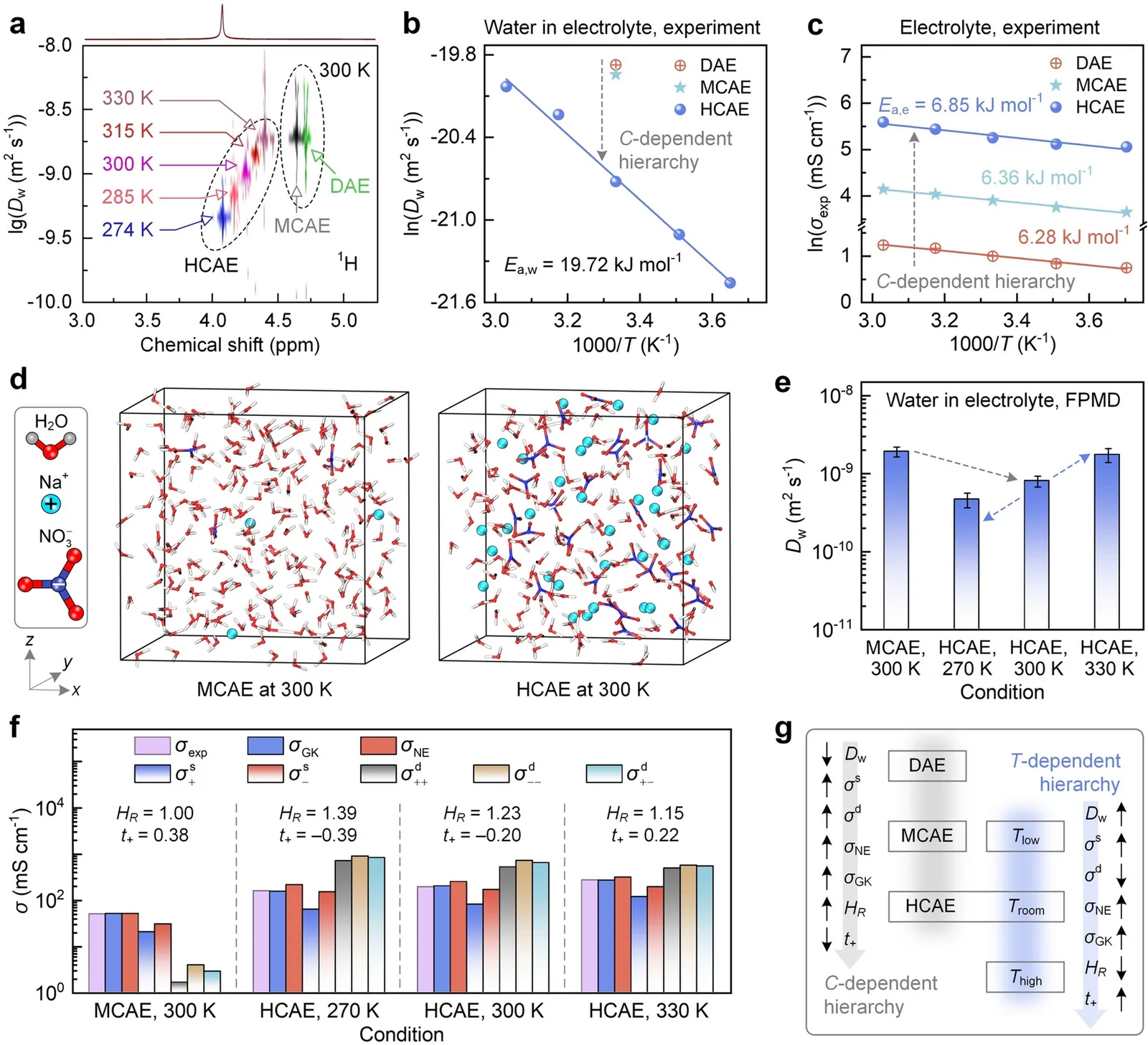
Environment-dependent transport performance of bulk aqueous electrolytes. **a** 2D DOSY spectrum of ^1^H nucleus obtained from PFG-NMR measurements. **b** and **c** Water diffusivity and ionic conductivity from experiments. **d**–**f** Snapshots of aqueous electrolyte, water diffusivity, and ionic conductivity from simulations, respectively. The error bars in panel (**e**) are standard deviations. Haven ratio (*H*_*R*_) and cation transference number (*t*_+_) are listed in panel (**f**). **g** Summary of environment-dependent transport performance

The peak shift reappears in ^1^H 2D DOSY (Fig. 4a), where *D*_w_ reduces with concentration at 300 K and grows with temperature in HCAEs. As shown in Fig. 4b and Table S2, from DAE to MCAE and HCAE at 300 K, *D*_w_ decreases from 23.4 × 10^−10^ to 21.8 × 10^−10^ and 10.0 × 10^−10^ m^2^ s^−1^, indicating the hindered water diffusion by high concentrations. From 274 to 330 K for HCAEs, *D*_w_ increases from 4.80 × 10^−10^ to 20.0 × 10^−10^ m^2^ s^−1^, implying the thermal-promoted water diffusion in HCAEs, with an energy barrier *E*_a,w_ of 19.72 kJ mol^−1^.

According to experimental measurements, Fig. 4c shows the electrolyte conductivity *σ*_exp_ as a function of concentration and temperature, and the data are shown in Table S3. *σ*_exp_ increases with concentration at a certain temperature. It changes from 2.71 mS cm^−1^ in DAE to 191.37 mS cm^−1^ in HCAE at 300 K due to more ion carriers. *σ*_exp_ also increases with temperature at a certain concentration. It rises from 157.07 mS cm^−1^ at 270 K to 268.37 mS cm^−1^ at 330 K in HCAE, due to the thermal-promoted ion movement. Notably, although DAE and MCAE present significant discrepancies in *σ*_exp_, their energy barriers are almost identical, with *E*_a,e_ values at 6.28 and 6.36 kJ mol^−1^, respectively. This consistency suggests that their electrolyte environments are largely similar, with comparable microscopic resistance for ion transport. In contrast, *E*_a,e_ increases to 6.9 kJ mol^−1^ in HCAEs, implying a significant variation in electrolyte environments. The densely packed ions in HCAEs intensify ion correlations and form ion aggregates that move collectively, thereby increasing the resistance to ion transport.

To reproduce experimental observations and offer atomic-level mechanistic explanations, FPMD simulations are performed for bulk aqueous electrolytes. Given the minor differences in solution environments between DAE and MCAE and the feasibility of model size, the 0.71 M solution is employed to represent MCAE, compared against the 7.29 M solution representing HCAE at 300 K. To evaluate the temperature-dependent NE deviation in HCAEs, simulations are also conducted at 270 and 330 K to generate outputs for comparison. Convergence tests for simulation parameters are provided in Fig. S4.

Figure 4d compares simulation snapshots of bulk MCAE and HCAE. Snapshots at different temperatures and the thermal equilibration are shown in Fig. S5. The simulated *D*_w_ agrees well with experiments (Fig. 4e, Table S2), and they reproduce the high-concentration suppressed and thermally enhanced *D*_w_. Figure 4f shows the simulated electrolyte conductivity and its partial contributions. These results are derived from EMSDs in Fig. S6, with data shown in Table S4. $\sigma_{{{\mathrm{GK}}}}$ calculated from the rigorous GK relation matches with experimental values *σ*_exp_, confirming the reliable simulation results. For MCAE at 300 K, $\sigma_{{{\mathrm{GK}}}}$ primarily originates from ion self-diffusion contributions (*i.e.*, $\sigma_{ + }^{{\mathrm{s}}}$ and $\sigma_{ - }^{{\mathrm{s}}}$, denoted as $\sigma^{{\mathrm{s}}}$), and contributions from ion-correlated motions (i.e., $\sigma_{ + + }^{{\mathrm{d}}}$, $\sigma_{ - - }^{{\mathrm{d}}}$, and $\sigma_{ + - }^{{\mathrm{d}}}$, denoted as $\sigma^{{\mathrm{d}}}$) are an order of magnitude lower. Electrolyte conductivity derived from the NE relation ($\sigma_{{{\mathrm{NE}}}}$) is thus almost equal to $\sigma_{{{\mathrm{GK}}}}$, with *H*_*R*_ at 1.00 and *t*_+_ at 0.38. Hence, the NE relation is acceptable for describing electrolyte transport in MCAEs.

With an increased concentration to HCAE at 300 K, *D*_+_ and *D*_–_ are reduced (Table S5) to imply the impeded ion self-diffusion. However, the ultrahigh concentration indicates more ion carriers to contribute $\sigma_{ + }^{{\mathrm{s}}}$ and $\sigma_{ - }^{{\mathrm{s}}}$. Eventually, the positive effect of increased carrier density outweighs the negative impact of slowed ion motion, leading to an enlarged $\sigma_{{{\mathrm{NE}}}}$. $\sigma_{{{\mathrm{NE}}}}$ even exceeds $\sigma_{{{\mathrm{GK}}}}$ in the HCAE, with *H*_*R*_ at 1.23 and a negative *t*_+_ at − 0.20, indicating the pronounced NE deviation. This deviation stems from the negatively charged aggregates due to the bound cation with multiple anions, so that cations are dragged to cause net negative contributions to electrolyte conductivity. The NE deviation is even temperature-dependent in HCAEs. $\sigma_{{{\mathrm{NE}}}}$ far exceeds $\sigma_{{{\mathrm{GK}}}}$ at 270 K, and *H*_*R*_ and *t*_+_ are 1.39 and − 0.39, respectively, indicating a significant NE deviation. At 330 K, *H*_*R*_ decreases to 1.15, and *t*_+_ returns to be positive (0.22), implying a mitigated NE deviation. Note that although these transport outputs are based on the limited model size (19.19^3^ Å^3^) and sampling time (24 ps), their statistical convergences are confirmed by the comparable results upon large model sizes and long simulation durations (Fig. S7).

Figure 4g summarizes the environment-regulated transport of bulk aqueous electrolytes. From DAE to HCAE, the increased ion carriers are accompanied by less water, resulting in crowded microenvironments and ion aggregates. This change limits the free space available for species motion and reduces *D*_w_. Meanwhile, the increased self-diffusion and ion-correlated contributions (*i.e.*, $\sigma^{{\mathrm{s}}}$ and $\sigma^{{\mathrm{d}}}$) enlarge $\sigma_{{{\mathrm{NE}}}}$ and $\sigma_{{{\mathrm{GK}}}}$. The enhancement in $\sigma^{{\mathrm{d}}}$ surpasses that in $\sigma^{{\mathrm{s}}}$, so *H*_*R*_ rises and *t*_+_ reduces, suggesting a growing NE deviation. In HCAEs, thermal effects disrupt aggregate structures, strengthen ion self-diffusion, and suppress ion correlations. $\sigma_{{{\mathrm{NE}}}}$ and $\sigma_{{{\mathrm{GK}}}}$ are thus enlarged with the increased *D*_w_, due to the promoted $\sigma^{{\mathrm{s}}}$ and suppressed $\sigma^{{\mathrm{d}}}$. *H*_*R*_ and *t*_+_ are thus reduced and ascended, respectively, implying an alleviated NE deviation.

### Concentration- and Thermal-Reconstructed Ion Correlation Networks in Bulk Electrolyte

**Fig. 5:**
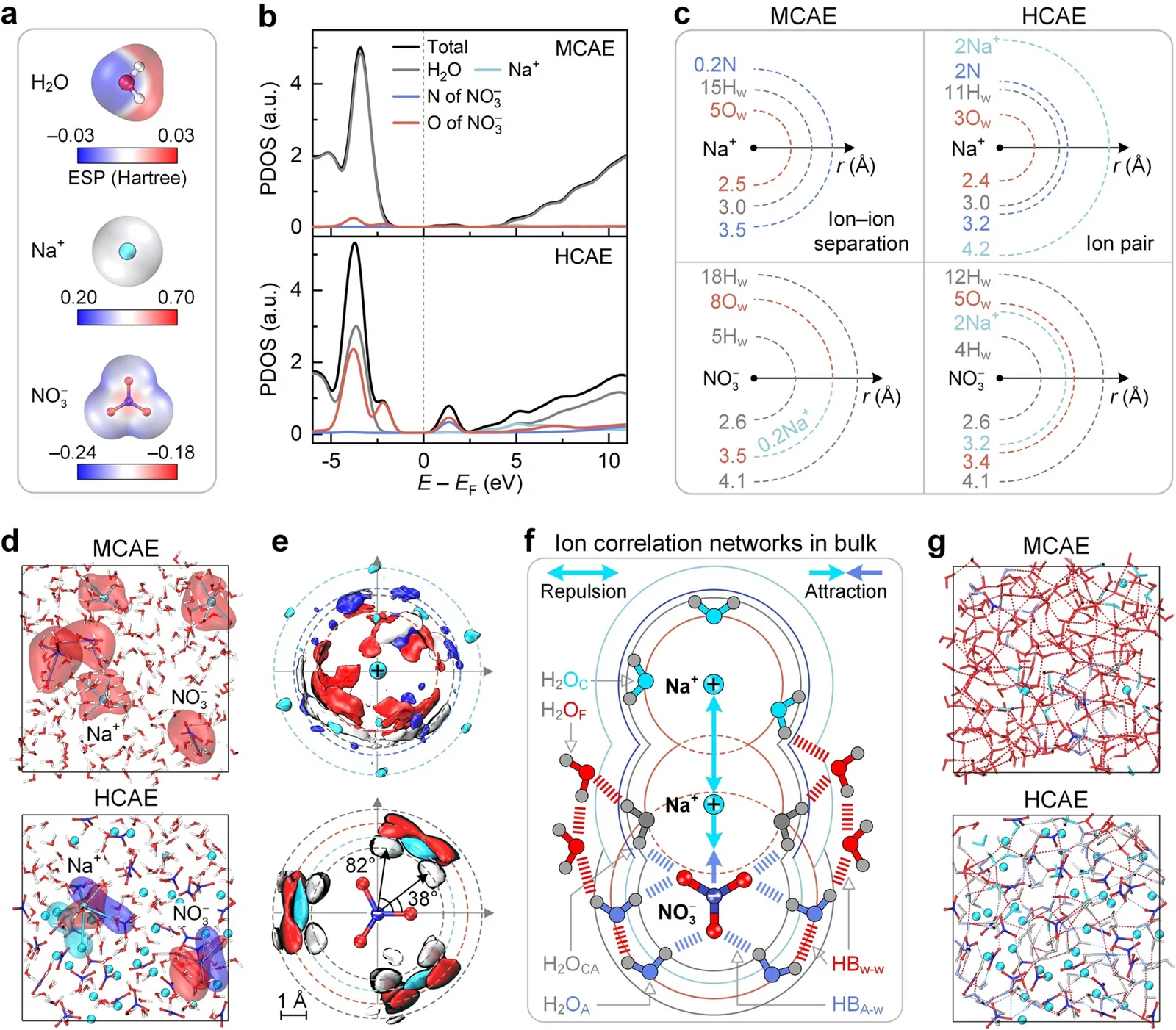
Graphical cognition of concentration-dependent ion correlation networks in bulk aqueous electrolytes. **a** ESP distribution of water molecule and ions. **b** PDOS of MCAE and HCAE. **c** Radial species distribution around ions. O_w_ and H_w_ denote water oxygen and hydrogen atoms, respectively, and N denotes NO_3_^−^ nitrogen atom. **d** Solvation configuration of cation and anion. Transparent areas in red, cyan, and blue are vdW volumes of the solvated water molecule, cation, and anion, respectively. **e** Spatial distribution function of cation and anion in HCAE. Color-filled volumes in red, white, cyan, and blue denote the space occupied by O, H, Na, and N atoms, respectively. **f** and **g** Schematic diagram and snapshots of ion correlation networks in bulk aqueous electrolytes, respectively. HB_w–w_ and HB_A–w_ denote water–water and anion–water H-bonds, respectively. H_2_O_F_, H_2_O_C_, H_2_O_A_, and H_2_O_CA_ denote water molecules in a free state, solely solvated by cation, solely solvated by anion, and co-solvated by cation and anion, respectively

The electronic, structural, and thermodynamic properties are then analyzed to explain the above phenomena. As shown in Fig. 5a, the polar water molecule has an uneven ESP distribution, serving as both H-bond donors and acceptors. Na⁺ and NO_3_^−^ ions have positive and negative ESP values, respectively. NO_3_^−^ even presents planar geometry and heterogeneous ESP distribution, enabling the HB_A–w_ formation. Combining the isolated species with diverse proportions yields bulk electrolytes at various concentrations. As shown in Fig. 5b, NO_3_^−^ has a certain contribution to the lowest unoccupied molecular orbital (LUMO) feature in MCAE, which stems from the intrinsic electronic structure of anions. As an enlarged concentration from MCAE to HCAE, ion correlations increase the NO_3_^−^ contribution to the LUMO feature, enabling the prioritized anion decomposition in HCAE. Such results highlight the superior electrochemical stability of HCAE, making it suitable for applications in energy conversion and storage.

The electronic properties endow unique structural features for HCAE. Figure 5c compares species distribution around ions, derived from the RDFs (Fig. S8). Na^+^ ion in MCAE features a water-rich hydration shell comprising five water oxygen (O_w_ at 2.5 Å) and 15 water hydrogen (H_w_). This pattern stems from ion–dipole interactions, where cations prefer to coordinate with lone pairs on water oxygen. NO_3_^−^ nitrogen atoms are sparsely distributed and distant from Na^+^, indicating an ion–ion separation. Moreover, five H_w_ and eight O_w_ (at 3.5 Å) appear around NO_3_^−^. Water hydrogen thus prefers to approach anions through H-bonds, resulting in a larger hydration shell with higher water content. In contrast, Na^+^ and NO_3_^−^ ions of HCAEs present less water in their hydration shells, and adjacent anions and cations are prevalent. This result implies the emergence of ion pairs through electrostatic interactions. The densely packed ion pairs enhance ion correlations and promote the formation of extensive aggregates. These results are supported by the cluster size distribution analyses (Fig. S9), inspired by previous reports [42–44].

As shown in Fig. 5d, we do not observe contact ion pairs in MCAE in the current simulation, indicating the absence of observable overlaps between cation and anion hydration shells. In contrast, ion hydration shells feature fewer water molecules and include counterions in HCAE, indicating notable alterations in ion coordination environments. These alterations are supported by ion topological structures (Fig. S10), inspired by a previous report [45]. Figure 5e shows the SDFs in HCAE to capture the directional information of such environments. Species around the Na^+^ ion distribute diffusely, whereas cations and water molecules exhibit distinct angular preferences around the NO_3_^−^ ion, attributed to the enhanced ion correlations and HB_A–w_.

Figure 5f illustrates ion correlation networks in HCAEs upon these observations, involving ion pairs, H-bonds, and solvation effects. The crowded environment generates ion pairs together with the coexisting HB_w–w_ and HB_A–w_. Water molecules have distinct occurrence states, including H_2_O_F_, H_2_O_C_, H_2_O_A_, and H_2_O_CA_. These interactions form ion aggregates and induce heterogeneity in HCAEs, as described in Fig. S11. Figure 5g compares snapshots of ion correlation networks. HB_w–w_ and H_2_O_F_ are dominant in MCAE, whereas HB_A–w_ and H_2_O_CA_ are prevalent in HCAE. Moreover, H-bond breaking and formation are explicitly observed between adjacent snapshots (Video S1), and water occurrence states undergo transient transitions in bulk MCAE and HCAE systems. These phenomena suggest the dynamic microstructures in aqueous electrolytes.

**Fig. 6:**
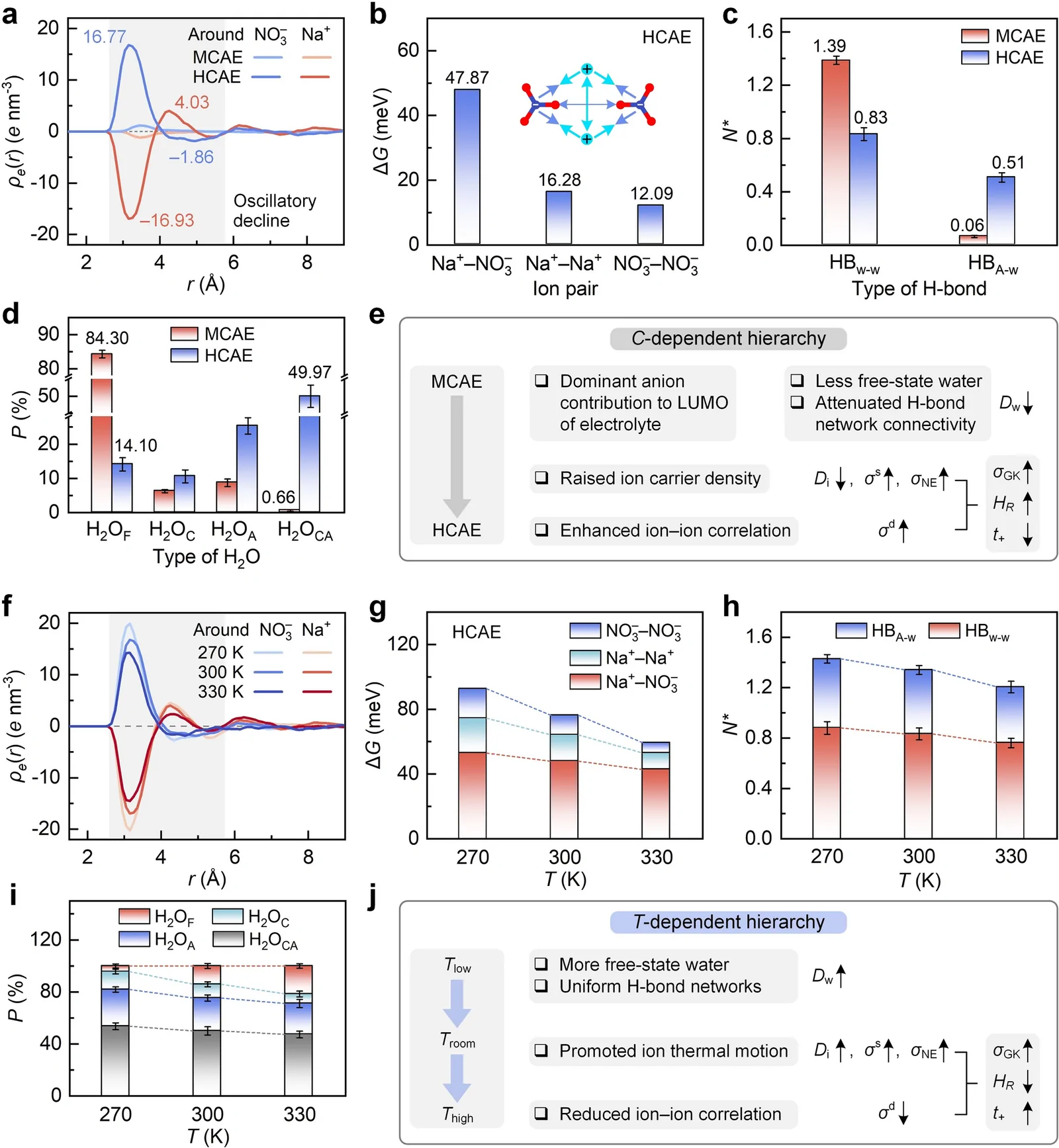
Quantified cognition of environment-regulated ion correlation networks in bulk aqueous electrolytes. **a** Concentration-regulated space charge density around ions. **b** Dissociation energy barrier of ion pair in HCAE. **c** and **d** Concentration-regulated normalized H-bond number and water proportion, respectively. **e** Concentration-regulated relationship between ion correlation network and transport performance. In panels (**a**–**e**), the concentration dependence is discussed at room temperature. **f** Temperature-regulated space charge density around ions. **g** Temperature-regulated dissociation energy barrier of ion pair. **h** and **i** Temperature-regulated normalized H-bond number and water proportion, respectively. **j** Temperature-regulated relationship between ion correlation network and transport performance. In panels (**f**–**j**), the temperature dependence is discussed focusing on HCAE

The above discussions reveal the concentration-dependent solvation configurations and ion correlation networks in bulk electrolytes from graphic insights, and such dependence is further quantified below. According to Fig. S12, the space around a central ion is primarily occupied by water molecules in MCAE, whereas it is held by extensive ions in HCAE, implying the altered charge distribution around ions. As shown in Fig. 6a, the radial space charge density *ρ*_*e*_(*r*) around the central ion remains near zero in MCAE, due to the screened ion–ion electrostatic interactions by water. In contrast, *ρ*_*e*_(*r*) successively presents a minimum of − 16.93 and a maximum of 4.03 around Na^+^ in HCAE, and it in turn shows a maximum of 16.77 and a minimum of − 1.86 around NO_3_^−^. These peak values stem from that ions in HCAE are surrounded by multiple counterions to form ion aggregates, as supported by the degree distribution of ion aggregate (Fig. S13), inspired by a previous report [45]. To quantify the ion pair strength, Fig. 6b compares the dissociation free energy barriers Δ*G* of ion pairs in HCAE, as derived from the potential of mean force in Fig. S14. Δ*G* reaches 47.87 kJ mol^−1^ for cation–anion pairs, followed by 16.28 and 12.09 kJ mol^−1^ for cation–cation and anion–anion pairs, respectively.

H-bond network strength in bulk electrolytes is evaluated from its configuration and number upon the geometric criterion (Fig. S15). Based on the H-bond length–angle colormap (Fig. S16) inspired by a previous report [46], HB_A–w_ is weaker and fewer than HB_w–w_ in bulk MCAE. As a transition from MCAE to HCAE, the role of strong HB_w–w_ is impaired, and the role of weak HB_A–w_ is enhanced. Based on their time-evolved results (Fig. S17), Fig. 6c shows the normalized number of H-bonds N*, which denotes the effective H-bond number formed per water molecule. N* reaches 1.39 for HB_w–w_ and 0.06 for HB_A–w_ in MCAE, whereas it drops to 0.83 for HB_w–w_ and rises to 0.51 for HB_A–w_ in HCAE. The concentration-dependent variation in H-bonds is supported by 2D maps of H-bond density (Fig. S18) and degree distribution of H-bond networks (Fig. S19), inspired by a previous report [45]. All these phenomena suggest the weakened H-bonds without fully breaking H-bond networks as an enlarged concentration, consistent with the concentration-induced shift of water frequency in IR spectra (Fig. 3b). Figure 6d presents the concentration-dependent proportions of water states, derived from the time-evolved results (Fig. S20). H_2_O_F_ and H_2_O_CA_ occupy 84.30% and 0.66% in MCAE, respectively, whereas H_2_O_F_ drops to 14.10% and H_2_O_CA_ rises to 49.97% in HCAE. Therefore, an ultrahigh concentration makes a dominance transition from free-state to bound-state water, which is unfavorable for water diffusion.

Upon these discussions, Fig. 6e shows the concentration-dependent association between ion correlation networks and transport behaviors in bulk electrolytes. The LUMO contribution becomes anion-dominated with a transition from MCAE to HCAE. The crowded environment then reduces free water and disrupts H-bond network connectivity, explaining the decreased *D*_w_ observed in experiments (Fig. 4b) and simulations (Fig. 4e). The raised ion carrier density even compensates for the hindered self-diffusion of individual ions due to ion crowding (reducing *D*_i_), enlarging $\sigma^{{\mathrm{s}}}$ and $\sigma_{{{\mathrm{NE}}}}$. It also enlarges $\sigma^{{\mathrm{d}}}$ by enhancing ion correlations. Eventually, $\sigma_{{{\mathrm{GK}}}}$ and *H*_*R*_ are magnified, and *t*_+_ is declined, explaining the concentration-dependent NE deviation.

Following the same route, ion correlation networks restructured by thermal effects in HCAE are examined. *ρ*_*e*_(*r*) amplitude reduces with rising temperature (Fig. 6f), suggesting the weakened ion correlation. Hence, the dissociation energy barriers (Δ*G*, Fig. 6g) decrease with temperature for all ion pairs, primarily reflecting the increased entropic contribution at higher temperatures. Ion pair dissociation and recombination are then shown by the probability distribution of cation–anion distance (Fig. S21), and a linear association between Δ*G* and *H*_*R*_ is further observed (Fig. S22), despite the limited data sample. Hence, the decrease in the dissociation energy barrier of ion pair accounts for reduced NE deviation at elevated temperatures. Moreover, the reduced N* values appear in both the strong HB_w–w_ (from 0.88 to 0.84 and 0.77) and weak HB_A-w_ (from 0.55 to 0.51 and 0.45) with a raised temperature from 270 to 300 and 330 K (Fig. 6h), suggesting a more uniform H-bond distribution, as verified in Fig. S18. This alteration indicates an enlarged proportion of HB_w–w_ from 61.54 to 62.22% and 63.11%. The temperature-dependent H-bond number is confirmed by the H-bond degree distribution (Fig. S19). Furthermore, the proportions of bound-state water (H_2_O_C_, H_2_O_A_, H_2_O_CA_) are decreased (Fig. 6i), along with an increased free-state water (H_2_O_F_) proportion from 4.36% to 14.10% and 21.56%. Thermal effects thus modulate H-bond strength and rearrange ion–water coordination in HCAEs simultaneously. These impacts explain the *v*-H_2_O peak shifting toward lower frequencies (Fig. 3c), and they further suggest an accelerated species diffusion under thermal effects.

As a summary (Fig. 6j), thermal heating induces less free-water fraction and homogeneous H-bond networks, thereby enhancing water diffusivity *D*_w_. Meanwhile, the enhanced ion motion promotes *D*_i_, $\sigma^{{\mathrm{s}}}$, and $\sigma_{{{\mathrm{NE}}}}$; the reduced ion–ion correlations suppress $\sigma^{{\mathrm{d}}}$. Eventually, these changes result in increased $\sigma_{{{\mathrm{GK}}}}$ and *t*_+_ as well as decreased *H*_*R*_, offering underlying explanations for the mitigated NE deviation with rising temperature.

### Interface-Reconstructed Ion Correlation Networks and Electrolyte Transport

The above discussions elucidate the change patterns and mechanisms of the environment-controlled NE deviation in bulk electrolytes. The insight expansion from bulk to interfaces will offer profound guidance for electrolyte design. Hence, this study establishes graphene–HCAE interfaces for FPMD simulations at 300 K. The outputs are compared with bulk results to reflect the interface-reconstructed ion correlation networks and NE deviations. FPMD simulations are further performed at different temperatures to probe the coupled effects of nanoconfinement and thermal conditions. These arrangements allow analysis of electronic and structural properties as well as transport performance related to the interface.

**Fig. 7:**
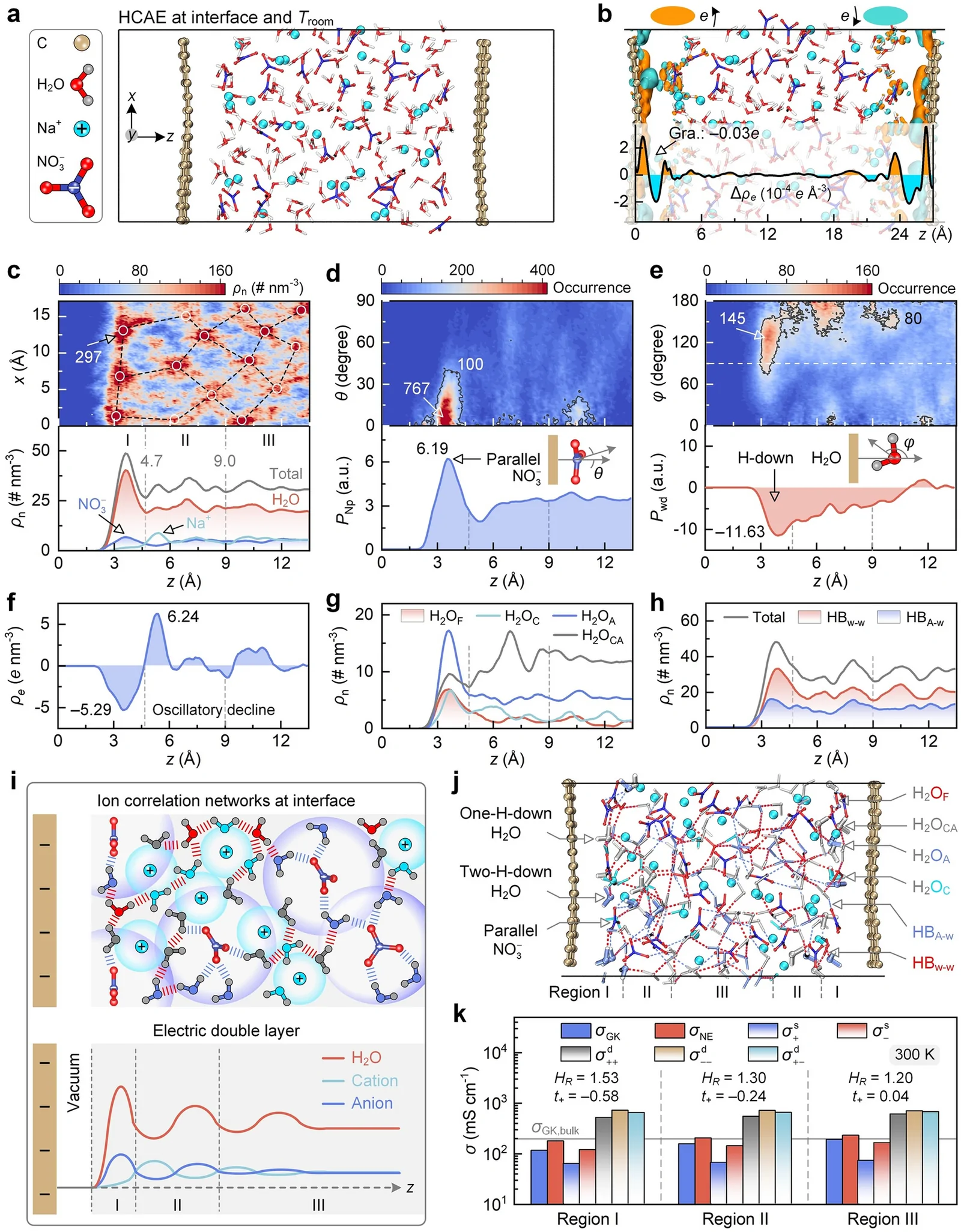
Room-temperature ion correlation networks and transport performance of HCAE at interfaces. **a** Snapshot of graphene–electrolyte interface. **b** Projected map and planar-averaged profile of electron density difference. Electron depletion and accumulation are displayed by orange and cyan with isosurfaces of 5 × 10^−4^ e Bohr^−3^, respectively. **c** Projected map of atomic number density and *z*-direction number density of species. White circles and black dashed lines denote ion correlation networks. Graphene surface is at the *z*-position of zero. **d** Distance–angle colormap and *z*-direction preference *P*_Np_ of anion orientation. *θ* is defined between the surface normal and the NO_3_^−^ plane normal. *θ* value near 0° denotes a parallel anion orientation. *P*_Np_ is the product of anion number density and the ensemble-averaged value of cos*θ*. The larger *P*_NP_ indicates a more pronounced tendency of parallel anion orientation. **e** Distance–angle colormap and *z*-direction preference *P*_wd_ of water dipole orientation. *φ* is defined between the surface normal and the opposite of water dipole. *φ* close to 128° and 180° denote one-H-down and two-H-down orientations, respectively. *P*_wd_ is the product of water number density and the ensemble-averaged value of cos*φ*. The maximum occurrence in panels (**c**–**e**) is marked by numbers. **f**–**h** *z*-direction number density of space charge, water molecules, and H-bonds, respectively. **i** Schematic diagram of ion correlation networks and EDL at interfaces. **j** Snapshot of ion correlation networks at interfaces. **k** Region-dependent ionic conductivities. Haven ratio (*H*_*R*_) and cation transference number (*t*_+_) are listed by numbers

Figure 7a shows a snapshot of the graphene–HCAE interface, and its thermal equilibration is shown in Fig. S23. Figure 7b depicts the electron density difference to provide an electronic-level insight into confinement-regulated electrolyte behaviors. The presence of surfaces induces slight redistributions of interfacial electron clouds, resulting in a negative Bader charge of − 0.03*e* on the surface. In terms of species spatial distribution (Fig. 7c), the atomic number density map presents multiple spot-like aggregation patterns, and the patterns are interconnected to form ion networks. Notably, a vacuum layer in 2.0 Å from the surface is devoid of any species, whereas the peak density of 297 nm^−3^ appears at 3.5 Å. One-dimensional (1D) number density (i.e., concentration) indicates a pronounced peak within 4.7 Å from the surface, attributed to surface adsorption. The density then oscillates slightly in subsequent regions. Upon these oscillations, the interface is divided into regions I (2.0–4.7 Å, nearest to the surface), II (4.7–9.0 Å, next-nearest to the surface), and III (9.0–13.5 Å, far from the surface).

Specifically, region I is water-dominated, and anion density is more enriched and closer to the surface than cations, although the surface has weakly negative charges. This feature stems from the coexisting ion adsorption and ion correlation. In detail, compared with Na^+^ ions, NO_3_^−^ ions with parallel orientation can approach the surface with a shorter distance and more negative adsorption energy (Fig. S24). Such anion adsorption with parallel orientation can overwhelm the weak electrostatic repulsion induced by surface charges, enabling anion accumulation closer to the surface than cation. Moreover, cation–anion correlation allows the formation of neutral or weakly charged clusters. These large-sized species (consisting of multiple cations and anions) occupy the interfacial space, apparently allowing anions to approach closer to the surface. In subsequent regions, alternating peaks of cation and anion concentrations are observed, and all species show oscillatory decay to uniform distributions. The region-dependent aggregations suggest spatial variations in ion transport mechanisms. Ions near the surface exhibit collective motion as aggregates, whereas those farther away engage in faster self-diffusion.

Species orientation preferences are also region-dependent at interfaces. Upon the definition of orientations (Fig. S25), the distance–angle colormap of anion orientation shows a maximum occurrence of 767 at *θ* near 0° and nearly zero occurrence at 90° in region I (Fig. 7d), indicating a preference of parallel orientation. In other regions, angle occurrences are diffusely distributed without preference. The 1D orientation preference of the anion plane (*P*_Np_) is further analyzed to reflect the coupling between anion concentration and its angle feature. *P*_Np_ has a peak of 6.19 in region I and flattens out in other regions. Hence, despite the limited timescale and system size in simulations, the results support the conclusions of parallel anion orientation in the region nearest to the surface and the spatial hierarchy of such orientation preference.

In terms of water dipole orientation (Fig. 7e), the high occurrence appears in a *φ* range of 80°–150° in region I, implying a preferred orientation at one-H-down structure. A *φ* range of 150–180° is then dominant with a two-H-down structure in region II, and angle occurrence is broadly distributed without specific orientation in region III. Meanwhile, the orientation preference of water dipole (*P*_wd_) has a minimum of − 11.63 in region I, and it decays toward zero in subsequent regions. Note that water alignment features emerge within the space up to 10 Å above the surface, attributed to the unique interfacial structure of HCAEs. In detail, water alignment at interfaces is the result of competition between the surface and bulk effects. Due to fewer HB_w–w_ in HCAEs than in MCAE (Fig. 6c), the impact of bulk H-bonds on the interfacial water alignment is reduced, and the ordered water pattern can extend to distant spaces. Moreover, ion aggregation and water alignment are concomitant in interfacial HCAEs. Water molecules, oriented by ions at interfaces, can alter water configuration in subsequent spaces through the correlations among hydrated ions, propagating water alignment features outward. Hence, water alignment above the surface can be regarded as an extension of water ordering at interfaces.

The spatial distribution and orientational preferences of HCAE components result in region-dependent behaviors. Figure 7f shows the spatial charge density *ρ*_*e*_ induced by the discrepancy in local concentrations of cation and anion. *ρ*_*e*_ is negative to − 5.29 *e* nm^−3^ in region I due to excess anions, and it transforms into positive to 6.24 *e* nm^−3^ in region II. *ρ*_*e*_ then fluctuates around zero in region III, indicating an oscillatory decay in net charge accumulation at interfaces. Figure 7g shows the number density of water molecules. H_2_O_A_ density is dominant in region I, followed by H_2_O_CA_, H_2_O_C_, and H_2_O_F_, attributed to anion enrichment and cation depletion in this region. H_2_O_CA_ density rises and then stabilizes in regions II and III, whereas the other water molecules decline. In terms of H-bond density (Fig. 7h), HB_w–w_ peaks in region I due to water enrichment, and it fluctuates in regions II and III due to water content oscillations. In contrast, HB_A–w_ always remains low due to fewer interfacial anions relative to water.

As a summary, Fig. 7i illustrates the EDL structure of interfacial HCAEs. Cation and anion hydration shells with distinct sizes nest with each other, thereby forming ion correlation networks and ion aggregates. Electrolyte constituents even present hierarchical distribution and oscillatory decay at interfaces. In region I nearest to the surface, anions are enriched with parallel orientation, and water has a content exceeding ions and emerges in H-down structures. These features lead to the enrichment of anion-solvated water and water–water H-bond networks. In region II, cation content surpasses anion, and the species orientation features are weakened. In region III, cation and anion concentrations are identical, and species orientation almost disappears. For graphic understanding, Fig. 7j displays a snapshot of ion correlation networks for interfacial HCAEs. Water states and H-bond types even show transient fluctuations over time (Video S2), along with the dynamic dissociation and combination of ion pairs (Fig. S26).

The structural features result in region-dependent transport behavior and NE deviation for interfacial HCAEs (Fig. 7k, Table S6). Compared with bulk HCAEs at 300 K (Fig. 4f), the high anion concentration in region I causes the highest conductivity contribution from anion–anion correlations ($\sigma_{ - }^{{\mathrm{d}}}$), whereas the self-diffusion contribution ($\sigma^{{\mathrm{s}}}$) is suppressed by the reduced ion diffusivity upon surface adsorption (Table S7). $\sigma_{{{\mathrm{NE}}}}$ and $\sigma_{{{\mathrm{GK}}}}$ are thus low and divergent from each other due to ion aggregation, corresponding to *H*_*R*_ of 1.53 and *t*_+_ of –0.58. In regions II and III, the reduced surface effect allows the weakened species aggregation, which restores $\sigma^{{\mathrm{s}}}$ and decreases $\sigma_{ - }^{{\mathrm{d}}}$. Hence, *H*_*R*_ decreases to 1.30 and 1.20, and *t*_+_ rises to − 0.24 and 0.04 in regions II and III, respectively. Note that the hierarchically reduction in ion–ion-correlated transport from regions I to III may partially stem from surface dielectric effects [47]. Specifically, the presence of a solid surface can reduce dielectric constants at interfaces, attributed to the anisotropic dipolar electric field and the excluded-volume effect of low-dielectric confining materials. In summary, such findings indicate a hierarchical deviation from the NE relation under nanoconfinement. This deviation is most significant in the nearest region to the surface, and it presents stepwise declines in subsequent regions and eventually approaches the deviation extent in bulk states.

### Interface–Thermal-Co-reconstructed Ion Correlation Networks and Electrolyte Transport

**Fig. 8:**
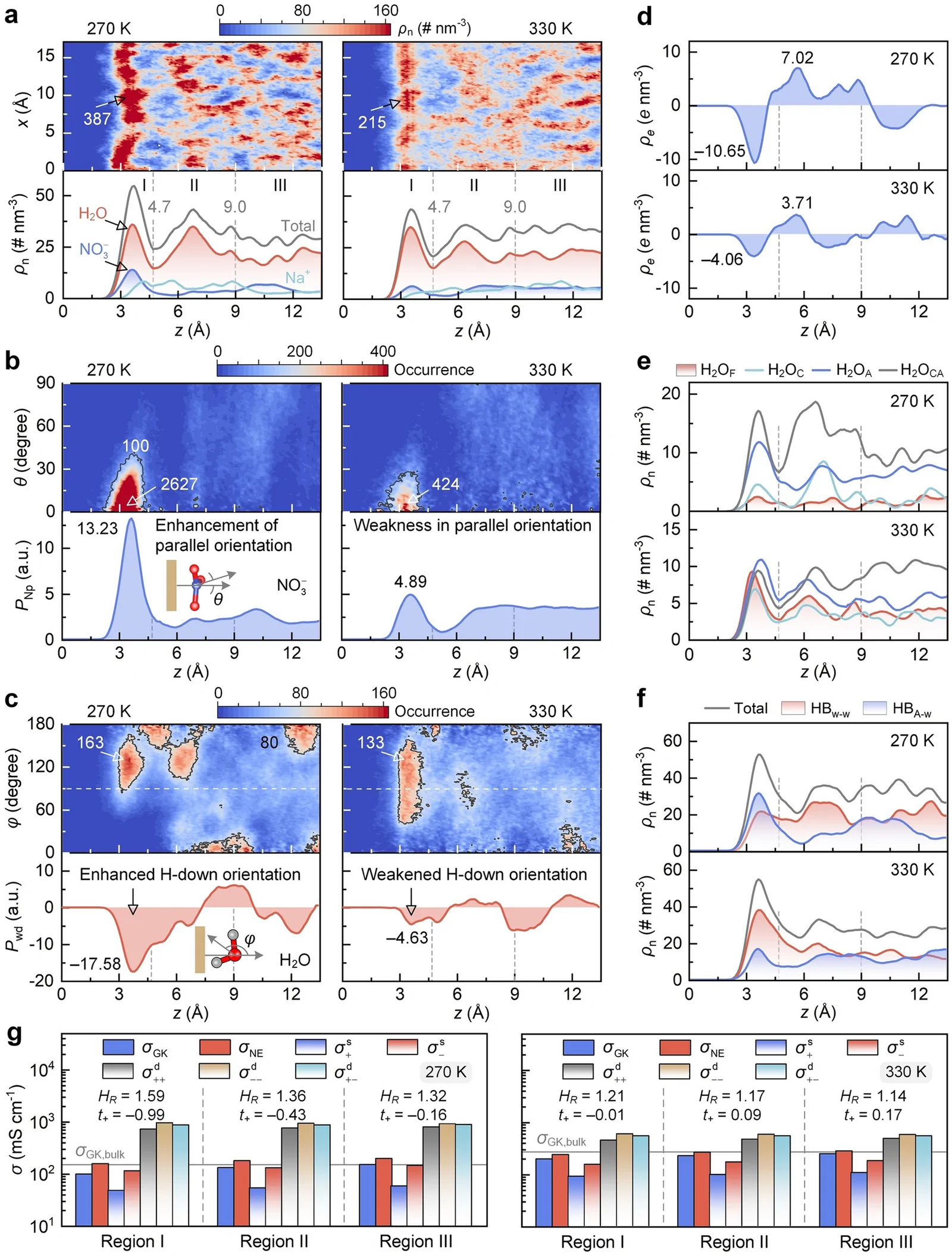
Temperature-dependent ion correlation networks and transport performance of HCAE at interfaces. **a** Projected map of atomic number density and *z*-direction number density of species. Graphene surface corresponds to the *z*-position of zero. **b** Distance–angle colormap and *z*-direction preference *P*_Np_ of NO_3_^−^ plane orientation. The larger *P*_NP_ value indicates a more pronounced tendency of parallel anion orientation. **c** Distance–angle colormap and *z*-direction *P*_wd_ preference of water dipole orientation. The maximum occurrence in panels (**a**–**c**) is listed by numbers. **d**–**f** *z*-direction number density of space charge, water molecules, and H-bonds, respectively. **g** Region-dependent ionic conductivities. Haven ratio (*H*_*R*_) and cation transference number (*t*_+_) are listed by numbers

FPMD simulations are then conducted at 270 and 330 K for interfacial HCAEs, and the snapshots and thermal equilibrations are shown in Fig. S23. This arrangement aims to probe the coupling impacts of thermal effect and nanoconfinement on ion correlation networks and the NE deviations. Compared with room-temperature outputs (Fig. 7c), region I has more pronounced aggregation patterns with a peak atomic number density of 387 nm^−3^ at 270 K (Fig. 8a), and the aggregation diminishes in regions II and III. Meanwhile, cation and anion densities increase in region I, leading to a higher total ion concentration due to the suppressed self-diffusion at a low temperature. In subsequent regions, species distributions exhibit oscillatory decay and eventually stabilize. By contrast, aggregation patterns are weakened in the entire space at 330 K. The density peak decreasing to 215 nm^−3^ in region I indicates the reduced species concentrations, and species distributions then approach homogeneity in regions II and III. These changes originate from the thermal-weakened anion adsorption, thereby reducing ion accumulation at interfaces.

The altered aggregation patterns further lead to changes in the orientation features of species at interfaces. Compared with room-temperature outputs (Fig. 7d), the distance–angle heatmap of anion orientation at 270 K shows an increased occurrence maximum of 2627 at *θ* near 0° in region I (Fig. 8b), and the occurrence pattern is absent in other regions. Meanwhile, the *P*_Np_ of 13.23 in region I is higher than that at 300 K (6.19), and it is flat in other regions. These results suggest that a low temperature enhances the parallel alignment of anions in the nearest region to the surface. In contrast, the occurrence pattern presents a maximum of 424 at 330 K, and the *P*_Np_ (4.89) in region I is lower than the room-temperature output. Hence, thermal heating weakens the parallel orientation of anions at the interface.

In terms of water dipole orientation (Fig. 8c), compared with a room-temperature condition (Fig. 7e), more occurrence patterns appear at 270 K, and they are concentrated in a *φ* range of 90°–150° in region I, with an increased maximum of 163 than that at room temperature (145). Meanwhile, *P*_wd_ is more negative (− 17.58) than that at room temperature (− 11.63) in region I, and it exhibits oscillatory decay toward zero in other regions. A low temperature thus reinforces water one-H-down orientation at interfaces. In contrast, the occurrence patterns shift toward a *φ* range of 40°–140° in region I at 330 K, lower than the *φ* range of 80°–150° observed at room temperature (Fig. 7e), and the occurrence peak decreases to 133. Furthermore, the negative peak of *P*_wd_ is − 4.63 in region I at 330 K. These findings imply the suppressed one-H-down structure of water molecules due to thermal heating.

The temperature-dependent distribution and orientation of constituents further modulate the HCAE behaviors at interfaces. Compared with room-temperature outputs (Fig. 7f), although *ρ*_*e*_ reserves an oscillatory decay at 270 K (Fig. 8d), the augmented discrepancy in cation and anion concentrations results in a more negative peak in region I (− 10.65 *e* nm^−3^) and a more positive peak in region II (7.02 *e* nm^−3^), suggesting the enlarged oscillation amplitude. Conversely, due to the narrowed difference between cation and anion concentrations at 330 K, *ρ*_*e*_ peaks in regions I (− 4.06 *e* nm^−3^) and II (3.71 *e* nm^−3^) approach zero, indicating the thermal-weakened charge oscillation. Moreover, compared with the room-temperature distribution (Fig. 7g), the H_2_O_CA_ accumulation feature is consolidated in region I at 270 K (Fig. 8e), whereas the contents of H_2_O_C_, H_2_O_A_, and H_2_O_F_ decrease. This alteration stems from the enhanced ion–ion correlations at low temperature, resulting in more water molecules in bound states. In contrast, H_2_O_CA_ and H_2_O_F_ densities fade and grow in region I at 330 K, respectively, leading to comparable contents among all water states. Hence, thermal effects dissociate aggregates and release water into free states. In addition, compared with the room-temperature output (Fig. 7h), the density peak of HB_A–w_ exceeds that of HB_w–w_ in region I at 270 K (Fig. 8f), whereas HB_A–w_ and HB_w–w_ densities descend and rise at 330 K, respectively. These changes stem from temperature-regulated concentrations of anion and water, thereby altering the H-bond network connectivity in interfacial HCAEs.

Upon the altered ion correlations, thermal effects affect the transport behaviors of HCAEs at interfaces (Fig. 8g). Compared with room-temperature outputs (Fig. 7k), a low temperature retards ion motions and enhances ion correlations, leading to depressed $\sigma^{{\mathrm{s}}}$ and enlarged $\sigma^{{\mathrm{d}}}$ in all regions. $\sigma_{{{\mathrm{NE}}}}$ and $\sigma_{{{\mathrm{GK}}}}$ thus decline, with more reduction in $\sigma_{{{\mathrm{GK}}}}$. These changes enlarge *H*_*R*_ to 1.59 and reduce *t*_+_ to − 0.99 in region I, implying an amplification of NE deviation. In contrast, a high temperature promotes ion self-diffusion and weakens ion correlations, producing the opposite outputs. Specifically, *H*_*R*_ decreases to 1.21 and *t*_+_ increases to − 0.01 in region I, implying the mitigated NE deviation. Therefore, thermal effects can restructure ion correlation networks at interfaces, thereby altering the hierarchical features of the NE deviation.

### Threefold Hierarchy of HCAE Transport

**Fig. 9:**
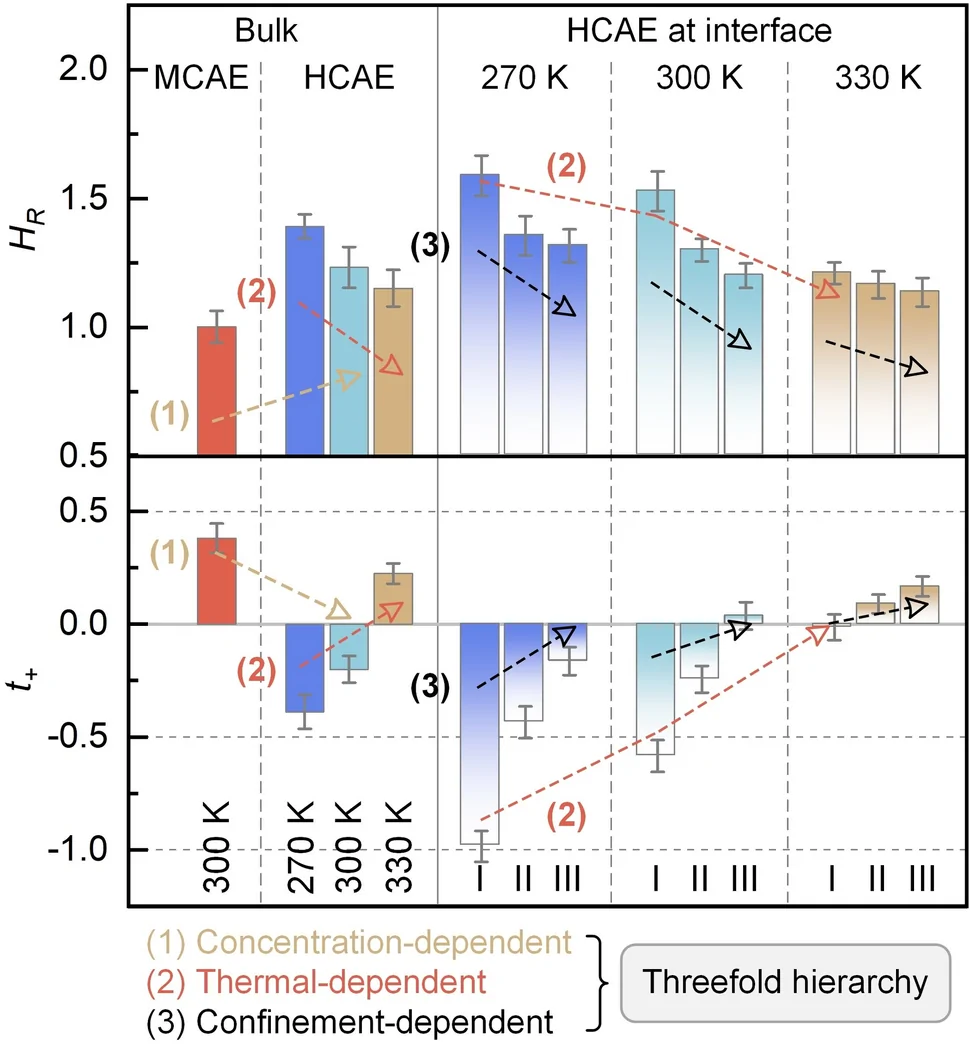
Threefold hierarchy of HCAE transport. *H*_*R*_ and *t*_+_ are Haven ratio and cation transference number, respectively. I, II, and III are the nearest-neighbor, next-near-neighbor, and far-away regions relative to the surface, respectively. Regions from I to III present a successive decline of nanoconfinement extent. The error bars denote standard deviations

According to the above findings, environmental factors can alter the electronic, structural, and thermodynamic properties, thereby reshaping the transport behaviors of aqueous electrolytes. Figure 9 summarizes the transport performance of aqueous electrolytes under diverse conditions of ion concentration, temperature, and nanoconfinement. These outputs are exhibited by taking NE deviations as descriptors and quantified by *H*_*R*_ and *t*_+_. In terms of room-temperature bulk electrolytes, HCAEs exhibit denser ion correlation networks than MCAEs and thus result in substantial NE deviation, as reflected by a *H*_*R*_ far greater than one and a negative *t*_+_. For bulk HCAEs, thermal effects can partially mitigate such deviation by weakening ion–ion correlations. When HCAEs are located at nanoconfined interfaces, the reshaped ion correlation networks induce a spatial hierarchy in the NE deviation. The strongest deviation emerges in the nearest region to the surface, as reflected by the highest *H*_*R*_ and the most negative *t*_+_. When HCAEs are concurrently modulated by nanoconfinement and thermal effects, all regions present the reduced NE deviation, as shown by the diminished *H*_*R*_ and positively shifted *t*_+_. Hence, HCAE transport behaviors present a threefold-hierarchical variation, i.e., from diluted to ultrahigh concentration, from low to high temperature, and from bulk to interface.

To confirm the extensibility of such hierarchy in other systems, LiTFSI aqueous electrolyte is then selected for probing its environment-controlled structure–transport relationship, given its widespread applications. These discussions rely on FPMD simulations under various conditions of ion concentration, temperature, and nanoconfined interface, with details in Note S3 and Fig. S27. Based on these results, compared with NaNO_3_ systems, the discrepant ion species and charges induce fluctuations in structural and transport outputs of LiTFSI systems. However, LiTFSI transport behaviors still present a threefold-hierarchical variation due to ion concentration, thermal effects, and nanoconfinement, *i.e.*, from diluted to ultrahigh concentration, from low to high temperature, and from bulk to nanoconfined interface. Hence, the threefold-hierarchical transport behaviors summarized in this study present a certain extensibility.

Inspired by the above threefold hierarchy, the accurate evaluation of electrolyte transport behaviors can no longer treat the entire electrolyte space as a single package, and the region-specific discussion is necessary from a localized insight. Since the NE relation shows deviation in capturing the HCAE transport and fails to respond to environmental changes, such a localized insight is valuable for modifying the NE relation. In detail, the NE relation modification relies on an integrated consideration of electrolyte transport regulated by ion concentration, thermal effects, and nanoconfinement. This regulation is based on variations in the underlying electronic, structural, and thermodynamic properties of aqueous electrolytes.

In addition to the salt concentration, thermal effects, and nanoconfinement, other factors in electrochemical devices may also alter electrolyte microstructure and reshape the NE deviation. These factors include the material nature and surface charge of solid substrates, the chemical reactivity and proton transfer behavior of electrolytes, as well as the strength and direction of interfacial electric fields. Hence, future modification of the NE relation may benefit from incorporating these factors into more complex expressions. The outputs would provide routes for predicting electrolyte transport performance in electrochemical energy devices.

## Conclusions

In summary, electrochemical characterizations and FPMD simulations are combined to perform the visualized and quantified investigations for bulk and interfacial aqueous electrolytes. The impacts of ion concentration, thermal effect, and nanoconfinement on transport behaviors are traced back to the alterations of electronic, structural, and thermodynamic properties. Bulk HCAEs at room temperature are found to exhibit rare free-state water and attenuated H-bond network connectivity. These features encourage the extensive formation of dense aggregates and ion correlation networks, leading to prominent deviations from the NE relation. Such deviation is mitigated by thermal effects due to the weakened microaggregates and ion–ion correlations in HCAEs. Ion correlation networks of HCAEs are further reshaped at interfaces, resulting in the oscillating decay distributions and position-dependent orientations of species. The NE relation thus presents hierarchical deviations, and the most prominent deviation is observed in the nearest region to the surface. Based on these findings, by taking NE deviations as descriptors, a threefold hierarchy of HCAE transport behaviors is summarized to depend on the concentration, thermal, and confinement factors. This hierarchical fingerprint is extensible to other aqueous electrolytes, linking the microstructural feature of environment-reconstructed ion correlation networks and the macroscopic transport performance of aqueous electrolytes. This work advances the underlying concept of ion transport from a localized insight at sub-nanometer resolution, guiding electrolyte reformation and operating condition regulation in electrochemical energy devices.

## Supplementary Information

Below is the link to the electronic supplementary material.

Supplementary file1 (DOCX 12401 KB)

Supplementary file2 (MP4 14904 KB)

Supplementary file3 (MP4 14771 KB)
