# Supplementary material for: Threefold-Hierarchical Transport of Highly Concentrated Aqueous Electrolyte Mediated by Environment-Reconstructed Ion Correlation Networks
Source: Nanomicro Lett. 2026 Feb 3;18:231. doi: 10.1007/s40820-026-02075-1 (PMC12868553; doi:10.1007/s40820-026-02075-1)
Supplement: Supplementary file 1 — Supplementary file1 (DOCX 12401 KB) [file 40820_2026_2075_MOESM1_ESM.docx]

Supporting Information for

**Threefold-Hierarchical Transport of Highly Concentrated Aqueous Electrolyte Mediated by Environment-Reconstructed Ion Correlation Networks**

Qiang Wang^1#^, Di Tian^1#^, Zhiguo Qu^1^*

^1^MOE Key Laboratory of Thermal-Fluid Science and Engineering, School of Energy and Power Engineering, Xi’an Jiaotong University, Xi’an 710049, P. R. China

^#^ Qiang Wang and Di Tian contributed equally to this work.

* Corresponding author. E-mail: [zgqu@mail.xjtu.edu.cn](mailto:zgqu@mail.xjtu.edu.cn) (Zhiguo Qu)

## Note S1 Definition of physical quantity

**S1.1 Physical quantity in experimental characterization**

Based on the Stejskal–Tanner equation [S1], the PFG-NMR signal intensity is related to the water diffusivity *D*_w_, as expressed by:

$D_{\text{w}}=-\ln\left[\frac{S\left(g\right)}{S\left(0\right)}\right]\frac{3}{\gamma^{2}g^{2}\delta^{2}\left(3\Delta-\delta\right)}$ (S1)

where *S*(*g*) and *S*(0) are the signal intensities under gradient strengths of *g* and zero, respectively. *γ* is the gyromagnetic ratio of the observed nucleus, *δ* is the duration of the gradient pulse, and *Δ* is the time between gradient pulses. A linear fit can be obtained between ln[*S*(*g*)] and *γ*^2^*g*^2^*δ*^2^(*Δ*–*δ*/3) by applying different *g* values, from which the slope yields the diffusivity *D*_w_. To extract the activation energy for solvent transport in HCAEs, *D*_w_ at diverse temperatures is further fitted using the Arrhenius equation:

$E_{\text{a,w}}=-RT\ln\frac{D_{\text{w}}}{D_{0}}$ (S2)

where *R*, *T*, and *D*_0_ are the universal gas constant, temperature, and fitting parameter, respectively.

To assess the concentration dependence of activation energy for electrolyte transport *E*_a,e_, the measured conductivity *σ*_exp_ is fitted using the Arrhenius equation:

$E_{\text{a,e}}=-RT\ln\frac{\sigma_{\text{exp}}}{\sigma_{0}}$ (S3)

where *σ*_0_ is a pre-exponential fitting parameter.

**S1.2 Physical quantity in FPMD simulation of bulk electrolyte**

In terms of electronic properties, the ESP distribution on the van der Waals (vdW) surface of isolated electrolyte components (cation, anion, and water molecule) reflects their charge features, and the vdW surface is the isosurface of electron density at 0.001 e Bohr^–3^. PDOS analysis can identify the component contribution to the lowest unoccupied molecular orbital (LUMO) energy level. The component dominating the LUMO is susceptible to reduction.

In terms of structural properties, the 2D density distributions of electrolyte components are analyzed to visualize their aggregation behaviors affected by concentration and temperature. RDF, also written as *g*(*r*), describes the spatial orderliness of species distribution around ions in a sphere-averaged manner:

$g\left(r\right)=\frac{\rho_{\text{b}}\left(r\right)}{\rho_{\text{b,avg}}}=\frac{V}{N_{\text{b}}}\left\langle\frac{\text{d}N_{\text{b}}\left(r\right)}{4\pi r^{2}\text{d}r}\right\rangle$ (S4)

where *ρ*_b_(*r*) is the number density (*i.e.*, *N*_b_(*r*)) of observed species *b* at a radial distance *r* from the central ion, and *ρ*_b,avg_ is the average number density of *b*, calculated as the number of *b* divided by system volume *V*. $\left\langle\ldots\right\rangle$ is the ensemble average. When calculating the RDF of NO_3_^–^, the nitrogen atom serves as the ion center. Coordination number (CN) and radial space charge can then be derived to quantify ion solvation structure. CN is defined as the number of *b* within a spherical region of radius *r* around the central ion:

$\text{CN}=\int_{0}^{r} 4\pi r^{2}\rho_{\text{b,avg}}g\left(r\right)\text{d}r$ (S5)

The radial space charge *ρ_e_*(*r*) is defined as the difference between the number density of cation and anion at distance *r* from the central ion:

$\rho_{e}\left(r\right)=\left[\rho_{\text{b,avg}}g\left(r\right)\right]_{+}-\left[\rho_{\text{b,avg}}g\left(r\right)\right]_{-}$ (S6)

This quantity defines the extent of ion aggregation around the central ion, serving as a structural indicator of ion–ion correlation strength. SDF is calculated to capture the angular and spatial orientation of species around an ion. It is defined as the ratio of the local number density *ρ*_b_(*x*, *y*, *z*) to the average number density *ρ*_b,avg_:

$\Omega\left(x,y,z\right)=\frac{\rho_{\text{b}}\left(x,y,z\right)}{\rho_{\text{b,avg}}}$ (S7)

For the H-bond network, H-bonds are considered present when the donor-acceptor distance is less than 3.5 Å and the donor-hydrogen-acceptor angle exceeds 140° (Fig. S15). To evaluate the H-bond network strength, H-bond length–angle colormaps are drawn. The averaged H-bond number *N*_HB,avg_ is then computed and then normalized as:

$N*=\frac{N_{\text{HB,avg}}}{N_{\text{w}}}$ (S8)

where *N*_w_ is the total number of water molecules. *N** denotes the effective number of H-bonds per water molecule, and its larger value indicates denser H-bond networks. Furthermore, the 2D distribution of H-bond density is analyzed to visualize the H-bond aggregation behaviors.

According to the classification of water occurrence states, H_2_O_CA_ encounters the strongest solvation effect due to interactions with both cation and anion, whereas H_2_O_F_ represents the free and unbound states. The proportion *P* of each water type is then calculated as:

$P=\frac{N_{\text{w,avg}}}{N_{\text{w}}}$ (S9)

where *N*_w,avg_ denotes the average number of each type of water molecule. *N*_w_ denotes the total number of water molecules. A lower number and proportion of H_2_O_F_ indicates that solvent water is less prone to reduction, suggesting higher electrochemical stability of electrolytes.

To assess ion–ion correlation strength from a thermodynamic insight, RDF between two species is transformed into the potential of mean force (PMF, also written as *ω*(*r*)):

$\omega\left(r\right)=-k_{\text{B}}T\ln\left[g\left(r\right)\right]$ (S10)

where *k*_B_ and *T* are the Boltzmann constant and temperature, respectively. Subsequently, the dissociation free energy barrier Δ*G* of an ion pair is defined as:

$\Delta G=\omega_{\max}-\omega_{\min}$ (S11)

where *ω*_min_ is the minimum PMF value at the first peak of *g*(*r*). *ω*_max_ is the maximum PMF value at the first valley of *g*(*r*). Δ*G* thus quantifies the energy barrier required for surrounding ions to dissociate from the central ion.

In terms of transport performance, water diffusivity *D*_w_ is calculated from the water mean square displacement (MSD):

$D_{\text{w}}=\frac{1}{6}\lim_{t\to\infty} \frac{\text{d}\left(\text{MSD}\right)}{\text{d}t}=\frac{1}{6}\lim_{t\to\infty} \frac{\text{d}}{\text{d}t}\left\langle\frac{1}{N_{\text{w}}}\sum_{i=1}^{N_{\text{w}}} \left[\Delta\overrightarrow{R}_{i}\left(t\right)\right]^{2}\right\rangle$ (S12)

where *N*_w_ is the total number of water molecules, and $\Delta\vec{R}_{i}\left( t \right)$ is the displacement vector of the *i*-th water molecule over time *t*. The 24-ps trajectories of bulk aqueous electrolytes are divided into three blocks, each with a length of 8 ps. A *D*_w_ value is then derived on each block by linear fitting of MSD profiles. The final *D*_w_ result is an average of the three derived values with a standard deviation.

For ionic conductivity in monovalent electrolytes in this study, the partial conductivities listed in Equation 2 in the main text are calculated by:

$\sigma_{+}^{\text{s}}=\frac{e^{2}}{6k_{\text{B}}TV}\lim_{t\to\infty} \frac{\text{d}\left(\text{EMSD}_{+}^{\text{s}}\right)}{\text{d}t}=\frac{e^{2}}{6k_{\text{B}}TV}\lim_{t\to\infty} \frac{\text{d}}{\text{d}t}\left\langle\sum_{i=1}^{N_{+}} \left[\Delta\overrightarrow{R}_{i}\left(t\right)\right]^{2}\right\rangle$ (S13)

$\sigma_{-}^{\text{s}}=\frac{e^{2}}{6k_{\text{B}}TV}\lim_{t\to\infty} \frac{\text{d}\left(\text{EMSD}_{-}^{\text{s}}\right)}{\text{d}t}=\frac{e^{2}}{6k_{\text{B}}TV}\lim_{t\to\infty} \frac{\text{d}}{\text{d}t}\left\langle\sum_{j=1}^{N_{-}} \left[\Delta\overrightarrow{R}_{j}\left(t\right)\right]^{2}\right\rangle$ (S14)

$\sigma_{++}^{\text{d}}=\frac{e^{2}}{6k_{\text{B}}TV}\lim_{t\to\infty} \frac{\text{d}\left(\text{EMSD}_{++}^{\text{d}}\right)}{\text{d}t}=\frac{e^{2}}{6k_{\text{B}}TV}\lim_{t\to\infty} \frac{\text{d}}{\text{d}t}\left\langle\sum_{i=1}^{N_{+}} \sum_{ii\neq i} \Delta\overrightarrow{R}_{i}\left(t\right)\cdot\Delta\overrightarrow{R}_{ii}\left(t\right)\right\rangle$ (S15)

$\sigma_{--}^{\text{d}}=\frac{e^{2}}{6k_{\text{B}}TV}\lim_{t\to\infty} \frac{\text{d}\left(\text{EMSD}_{--}^{\text{d}}\right)}{\text{d}t}=\frac{e^{2}}{6k_{\text{B}}TV}\lim_{t\to\infty} \frac{\text{d}}{\text{d}t}\left\langle\sum_{j=1}^{N_{-}} \sum_{jj\neq j} \Delta\overrightarrow{R}_{j}\left(t\right)\cdot\Delta\overrightarrow{R}_{jj}\left(t\right)\right\rangle$ (S16)

$\sigma_{+-}^{\text{d}}=\frac{e^{2}}{6k_{\text{B}}TV}\lim_{t\to\infty} \frac{\text{d}\left(\text{EMSD}_{+-}^{\text{d}}\right)}{\text{d}t}=\frac{e^{2}}{6k_{\text{B}}TV}\lim_{t\to\infty} \frac{\text{d}}{\text{d}t}\left\langle\left[\sum_{i=1}^{N_{+}} \Delta\overrightarrow{R}_{i}\left(t\right)\right]\cdot\left[\sum_{j=1}^{N_{-}} \Delta\overrightarrow{R}_{j}\left(t\right)\right]\right\rangle$ (S17)

Here, EMSD refers to the effective MSD contributed by various ion motion components: self-diffusion of cation and anion ($\text{EMSD}_{+}^{\text{s}}$ and $\text{EMSD}_{-}^{\text{s}}$, respectively) as well as correlated motion between cation–cation, anion–anion, and cation–anion pairs ($\text{EMSD}_{++}^{\text{d}}$, $\text{EMSD}_{--}^{\text{d}}$, and $\text{EMSD}_{+-}^{\text{d}}$, respectively). *N*_+_ and *N*_–_ are the numbers of cation and anion. *e*, *k*_B_, *T*, and *V* are the elementary charge, Boltzmann constant, temperature, and system volume, respectively. $\Delta\vec{R}_{i}\left( t \right)$ and $\Delta\vec{R}_{j}\left( t \right)$ are the displacement vectors of cation *i* and anion *j*, respectively. A partial conductivity is also derived on each block by linear fitting of such EMSD profiles for bulk aqueous electrolytes. The eventual partial conductivity is an average of the three derived values.

By comparing the definitions of MSD and EMSD, $\text{EMSD}_{+}^{\text{s}}$ is the product of cation MSD and its number *N*_+_, and $\text{EMSD}_{-}^{\text{s}}$ is the product of anion MSD and its number *N*_–_. Hence, $\sigma_{+}^{\text{s}}$ and $\sigma_{-}^{\text{s}}$ can be rewritten as:

$\begin{matrix} \sigma_{+}^{\text{s}}=\frac{N_{+}e^{2}}{6k_{\text{B}}TV}\lim_{t\to\infty} \frac{\text{d}}{\text{d}t}\left\langle\frac{1}{N_{+}}\sum_{i=1}^{N_{+}} \left[\Delta\overrightarrow{R}_{i}\left(t\right)\right]^{2}\right\rangle\\ =\frac{N_{+}e^{2}}{k_{\text{B}}TV}\frac{1}{6}\lim_{t\to\infty} \frac{\text{d}\left(\text{MSD}_{+}^{\text{s}}\right)}{\text{d}t}=\frac{N_{+}e^{2}}{k_{\text{B}}TV}D_{+}=\frac{F^{2}}{RT}c_{+}D_{+} \end{matrix}$ (S18)

$\begin{matrix} \sigma_{-}^{\text{s}}=\frac{N_{-}e^{2}}{6k_{\text{B}}TV}\lim_{t\to\infty} \frac{\text{d}}{\text{d}t}\left\langle\frac{1}{N_{-}}\sum_{j=1}^{N_{-}} \left[\Delta\overrightarrow{R}_{j}\left(t\right)\right]^{2}\right\rangle\\ =\frac{N_{-}e^{2}}{k_{\text{B}}TV}\frac{1}{6}\lim_{t\to\infty} \frac{\text{d}\left(\text{MSD}_{-}^{\text{s}}\right)}{\text{d}t}=\frac{N_{-}e^{2}}{k_{\text{B}}TV}D_{-}=\frac{F^{2}}{RT}c_{-}D_{-} \end{matrix}$ (S19)

These expressions imply that the self-diffusion conductivity arises from coupled contributions of electrolyte temperature, ion concentration, and ion diffusivity.

**S1.3 Physical quantity in FPMD simulation of HCAE at interfaces**

In terms of electronic property, the extent of interfacial charge redistribution is visualized by the electronic density difference $\Delta\rho_{e}$:

$\Delta\rho_{e}=\rho_{\text{sys}}-\rho_{\text{S}}-\rho_{\text{L}}$ (S20)

where $\rho_{\text{sys}}$, $\rho_{\text{S}}$, and $\rho_{\text{L}}$ are the electron densities of the entire interface, solid surface, and liquid electrolyte, respectively. The plane-averaged $\Delta\rho_{e}$ and Bader charge analysis are further used to quantify interfacial electron transfer and surface charge.

In terms of structural property, the 2D atomic number density $\rho_{\text{n}}$ visualizes the interfacial aggregation of constituents:

$\rho_{\text{n}}=\frac{\sum_{i} \Delta y_{i}\rho_{i}}{\sum_{i} \Delta y_{i}}$ (S21)

where Δ*y_i_* is the grid length in the *y*-direction and $\rho_{i}$ is the atomic number density at grid point *i*. The vertical number density $\rho_{\text{n}}\left( z \right)$ is then calculated for cation, anion, and water molecules:

$\rho_{\text{n}}\left(z\right)=\frac{\sum_{ij} \Delta x_{i}\Delta y_{j}\rho_{i,j}}{\sum_{ij} \Delta x_{i}\Delta y_{j}}$ (S22)

where Δ*x_i_* and Δ*y_j_* are the grid lengths along *x* and *y* directions, respectively. $\rho_{i,j}$ is the number density at the grid point (*i*, *j*). Upon the summed number density of all components, the interfacial region is divided into regions I, II, and III, which are nearest, next nearest, and far away relative to the surface, respectively. Similar distributions are also calculated for spatial charge density, four types of water molecules, and two types of H-bonds.

To describe anion orientations at interfaces, the angular distribution function (ADF) is computed to capture the probability distribution of angle *θ* between two position vectors $kl$ and $km$:

$\theta=\left\langle\cos^{-1}\left(\frac{r_{kl}^{2}+r_{km}^{2}-r_{lm}^{2}}{2r_{kl}r_{km}}\right)\right\rangle$ (S23)

where *k* is the shared origin point. *l* and *m* are endpoints of vectors. *r* is the point-point distance, and $\left\langle\ldots\right\rangle$ denotes ensemble averaging. In this study, two vectors are defined along the surface normal direction and perpendicular to the NO_3_^–^ plane, respectively. An angle *θ* close to 0° and 90° denotes parallel and vertical anion orientations relative to surfaces, respectively. To quantify the distance dependence of anion orientation, the point-plane distance function (PLDF) is employed to represent the ensemble-averaged NO_3_^–^–surface distance. The combined distribution function (CDF) of ADF and PLDF is then used to generate 2D colormaps, thereby visualizing the coupled distance–angle dependence of anion orientation at interfaces. Moreover, to demonstrate the synergistic effect between the angle *θ* and anion number density $\rho_{\text{n}}$, the anion-plane orientation preference *P*_NP_ is defined as:

$P_{\text{NP}}=\rho_{\text{n}}\left\langle\cos\theta\right\rangle$ (S24)

where $\left\langle\ldots\right\rangle$ denotes an average over the lateral plane and simulated ensemble. *θ* range is from 0° to 90°. The larger *P*_NP_ indicates a more pronounced tendency of parallel anion orientation.

The same idea is applied to capture water orientation at interfaces. In detail, the angle *φ* is defined between the surface normal and the opposite of water dipole, and its ADF is combined with PLDF to form the corresponding CDF. *φ* range is from 0° to 180°. *φ* near 128° and 180° denote one-H-down and two-H-down structures, respectively. To quantify the synergism between the angle *φ* and water number density, water dipole preference *P*_wd_ is defined as:

$P_{\text{wd}}=\rho_{\text{n}}\left\langle\cos\varphi\right\rangle$ (S25)

Two *φ* values with the same separation from 90° suggest a distinct water dipole orientation. In this situation, the positive *P*_wd_ indicates a dominant orientation with *φ* below 90°, whereas the negative *P*_wd_ indicates a dominant orientation with *φ* over 90° (H-down orientation, as shown in Fig. S25). The larger absolute value of *P*_wd_ even denotes a more pronounced tendency of such water orientation.

In addition, the 40-ps trajectories of interfacial HCAEs are divided into four blocks, each with a length of 10 ps. The partial conductivity is also calculated from block averaging.

## Note S2 Computational details

**S2.1 Basic parameters**

In this study, all simulations are conducted in the CP2K/Quickstep package [S2, S3]. Electronic structure calculations are performed using density functional theory (DFT) within the hybrid Gaussian and plane wave framework [S4]. The 1s electron of H, 2s and 2p electrons of C, O, and N, as well as 2s, 2p, and 3s electrons of Na, are treated as valence electrons with the double-ζ valence polarization (DZVP-MOLOPT-SR-GTH) basis set [S5], while the electronic cores are represented by Goedecker-Teter-Hutter pseudopotentials [S6, S7]. Perdew-Burke-Ernzerhof (PBE) functionals are used to describe the exchange-correlation effects [S8], and the Grimme D3 method is used to perform the dispersion correction [S9, S10]. The orbital transformation (OT) optimization algorithm is used [S11], and the direct inversion in the iterative subspace (DIIS) minimizer is used for self-consistent field convergence, ensuring the energy is converged to 2 × 10^–6^ Hartree.

The absolute and relative energy cutoffs (*i.e.*, *E*_cutoff_ and *E*_cutoff,rel_) in the CP2K are tested to balance the accuracy and efficiency. In detail, the coarsely optimized graphene–electrolyte configuration (consisting of Na^+^ ions, NO_3_^–^ ions, water molecules, and graphene surfaces) is extracted, aiming to evaluate the force applied on each atom at various combinations of *E*_cutoff_ and *E*_cutoff,rel_. The root mean squared errors (RMSE) of atomic forces along *x*, *y*, and *z*-direction are then calculated by

$\text{RMSE}_{F}=\sqrt{\frac{1}{3N}\sum_{i=1}^{3N} \left(F_{i}-F_{i,0}\right)^{2}}$ (S26)

where *N* is the number of atoms in a system, *F_i_* is the atomic force at the tested *E*_cutoff_ and *E*_cutoff,rel_, *F_i_*_,0_ is the atomic force at *E*_cutoff_ and *E*_cutoff,rel_ of 2000 and 200 Ry, respectively, serving as the benchmark. According to the test results shown in Fig. S4, *E*_cutoff_ and *E*_cutoff,rel_ of 400 and 40 Ry are used for all simulations.

**S2.2 FPMD simulations**

As a branch of FPMD, second-generation Car-Parrinello molecular dynamics (SG-CPMD) is an efficient and accurate method due to the combined Car-Parrinello and Born-Oppenheimer molecular dynamics [S12]. It employs DFT to calculate interatomic forces in the nuclear equation of motion based on the Born–Oppenheimer approximation, and it retains large integration time steps and preserves calculation efficiency simultaneously. Following this idea, SG-CPMD simulations are conducted for all systems with periodic boundary conditions in all directions. The always stable predictor-corrector (ASPC) method is used at the order *k* = 2 for the propagation of electronic degrees of freedom [S13]. The simulations then propagate the equations of motion based on the modified Langevin equation [S14], as expressed by

 (S27)

where *M_I_* and ***R****_I_* are ionic mass and coordinate, respectively. **F**_BO_ is the exact but inherently unknown Born–Oppenheimer (BO) force. **F**_ASPC_ is the force calculated from the ASPC method. **Ξ***_I_* is a random noise as the sum of **Ξ**_I_ and **Ξ**_L_. To guarantee an accurate sampling of the Boltzmann distribution, **Ξ***_I_* should obey the following fluctuation-dissipation theorem

$\left\langle\mathbf{\Xi}_{I}\left(0\right)\mathbf{\Xi}_{I}\left(t\right)\right\rangle=6\left(\gamma_{\text{L}}+\gamma_{\text{D}}\right)M_{I}k_{\text{B}}T\delta\left(t\right)$ (S28)

where *k*_B_ is the Boltzmann constant, *T* is the absolute temperature, and *t* is the time. *γ*_L_ is a Langevin friction coefficient, and it is set to 0.005 fs^–1^. *γ*_D_ is a kind-dependent damping coefficient to mimic the dissipation, and it is adjusted by running pre-equilibration simulations to realize the energy conservation and the convergence at the correct target temperature (270, 300, and 330 K). In this study, *γ*_D_ is set to 7 × 10^–4^ fs^–1^ for Na^+^ and NO_3_^–^ ions, 2.2 × 10^–4^ fs^–1^ for water molecules, and 1 × 10^–5^ fs^–1^ for the graphene surfaces. The correction step is obtained by five iterations.

For the bulk aqueous electrolyte systems with concentration and temperature regulations, the initial 3-ps of trajectory is regarded as the equilibrium period, followed by the production period of 24 ps. For the graphene–electrolyte interface systems with temperature regulations, one carbon atom on each graphene surface is fixed to maintain the thickness of the vacuum layer. The initial 3-ps trajectory is regarded as the equilibrium period, followed by the production period of 40 ps. The time step is set to 1 fs. The energy conservation and thermal equilibrium in the production periods confirm the parameter rationality arranged in this study. After simulations, the data analyses are performed using Multiwfn [S15], TRAVIS [S16, S17], and self-developed Python scripts, and VMD is used for visualization [S18].

## Note S3 Extensibility of threefold-hierarchical transport of HCAEs

The threefold hierarchy of HCAE transport behaviors in the main text is derived based on NaNO_3_ aqueous electrolytes. To explore the extensibility of such a hierarchy in other aqueous systems, LiTFSI aqueous electrolyte is then selected as a representative for FPMD simulations, considering its widespread role in electrochemical energy conversion and storage. The discussed systems cover various conditions in ion concentration, temperature, and nanoconfinement. The obtained results enable an exploration of LiTFSI transport affected by environmental factors. For comparative analysis, the working conditions, model setup, simulation details, and post-processing procedures for LiTFSI electrolytes are identical to those for NaNO_3_ electrolytes. Figure S27 shows the critical outputs for evaluating the electrolyte microstructures, including ion correlation networks, H-bonds, and water occurrence states. The transport behaviors are quantified by Haven ratio *H_R_* and cation transference number *t*_+_.

The results indicate that bulk MCAE (LiTFSI) is dominated by water-water H-bonds (HB_w-w_) and free-state water molecules (H_2_O_F_) at room temperature, as shown in Fig. S27a, S27c, and S27d. In contrast, bulk HCAE (LiTFSI) forms ion correlation networks dominated by anion-water H-bonds (HB_A-w_) and cation-anion co-solvated water molecules (H_2_O_CA_), as shown in Fig. S27b, S27c, and S27d. Compared with NaNO_3_ aqueous electrolytes, the discrepant ion species and charges result in certain variations in the H-bond number and water proportion in LiTFSI electrolytes. However, the qualitative conclusions remain that the ultrahigh concentration can reshape ion correlation networks. Such networks of HCAE (LiTFSI) are further redrawn by the nanoconfined interface (Fig. S27e). Similarly, thermal effects can weaken the correlation strength within both bulk and interfacial LiTFSI electrolytes, which is not detailed here.

Figure S27f presents the environment-controlled transport features of LiTFSI aqueous electrolytes. Although the *H_R_* and *t*_+_ values differ from those of NaNO_3_ systems to a certain extent, they consistently exhibit the threefold-hierarchical variations induced by ion concentration, thermal effects, and nanoconfinement, *i.e.*, from diluted to ultrahigh concentration, from low to high temperature, and from bulk to interface. Therefore, the threefold-hierarchical transport behaviors summarized in this study present a certain extensibility.

**Supplementary Figures**


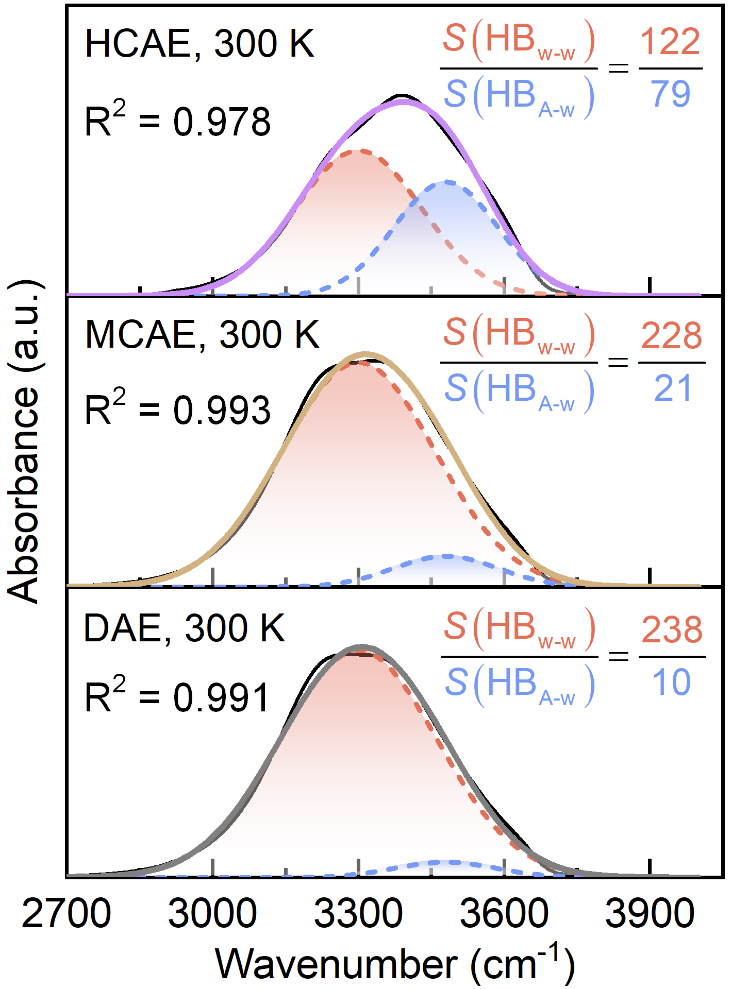


**Fig. S1** Gaussian fitting-based decomposition of the *v*-H_2_O of IR spectra for bulk aqueous electrolytes at 300 K. Black lines denote the experimentally measured *v*-H_2_O band reproduced from Fig. 3b in the main text. *S*(HB_w-w_) and *S*(HB_A-w_) denote the peak areas contributed from strong HB_w-w_ (red dashed line) and weak HB_A-w_ (blue dashed line), respectively

As an increased ion concentration from DAE to MCAE and HCAE, the ratio of *S*(HB_w-w_) to *S*(HB_A-w_) is altered from 238/10 to 228/21 and 122/79. This phenomenon implies an impaired role of strong HB_w-w_ and an enhanced role of weak HB_A-w_, suggesting the attenuated H-bond networks without fully breaking.


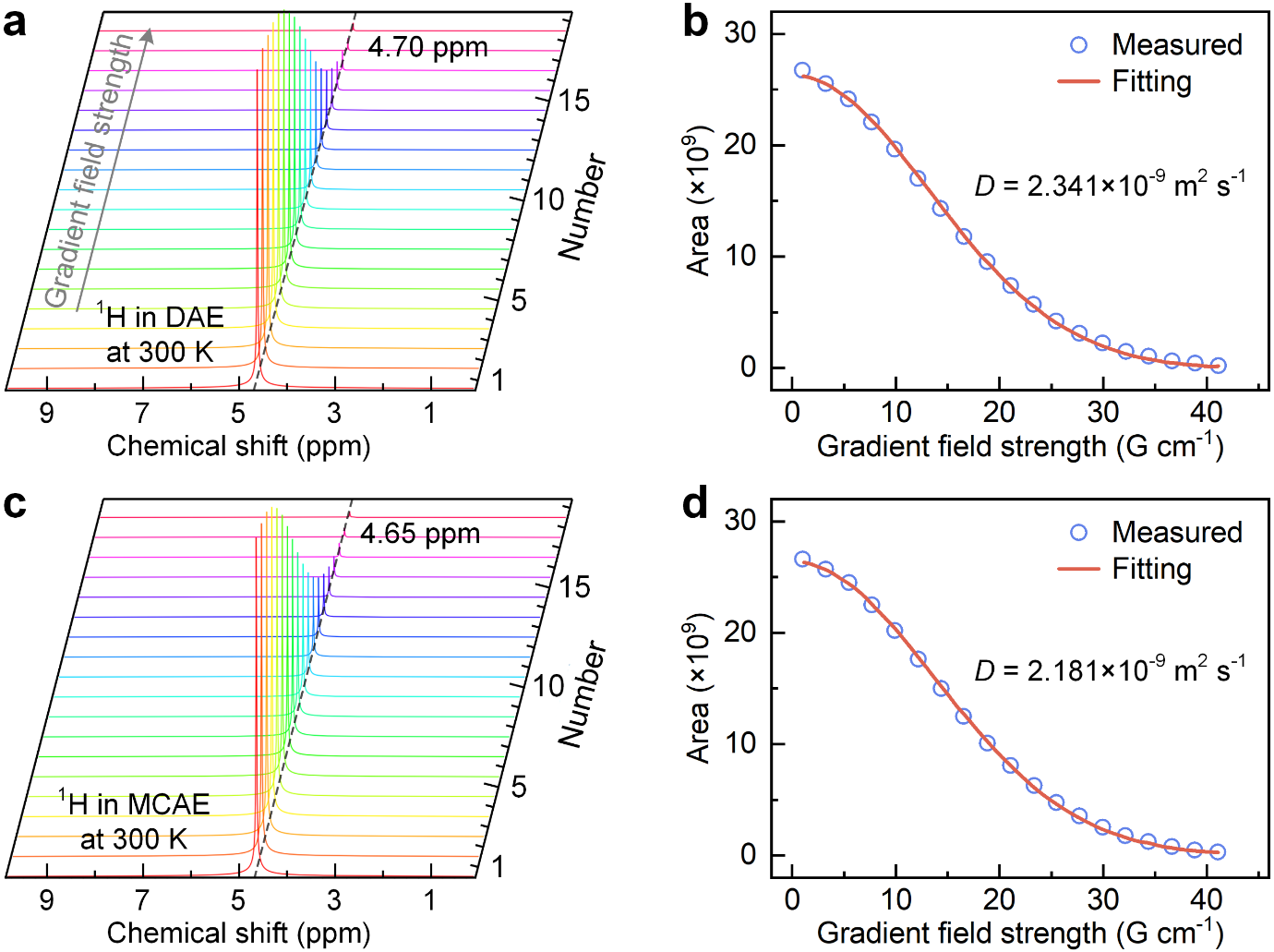


**Fig. S2** PFG-NMR results of ^1^H nucleus in bulk aqueous electrolytes. **a–b** PFG-NMR spectrum and diffusion peak area in DAE at 300 K, respectively. **c–d** PFG-NMR spectrum and diffusion peak area in MCAE at 300 K, respectively. In panels (b) and (d), blue points show the measured data, and the red line is a non-linear fit. Panels (a) and (c) are reproduced from Fig. 3d in the main text

The peak position of chemical shift in DAE is at 4.70 ppm, which is almost identical to that in MCAE (4.65 ppm). The differences in solution environments between DAE and MCAE are thus minor.


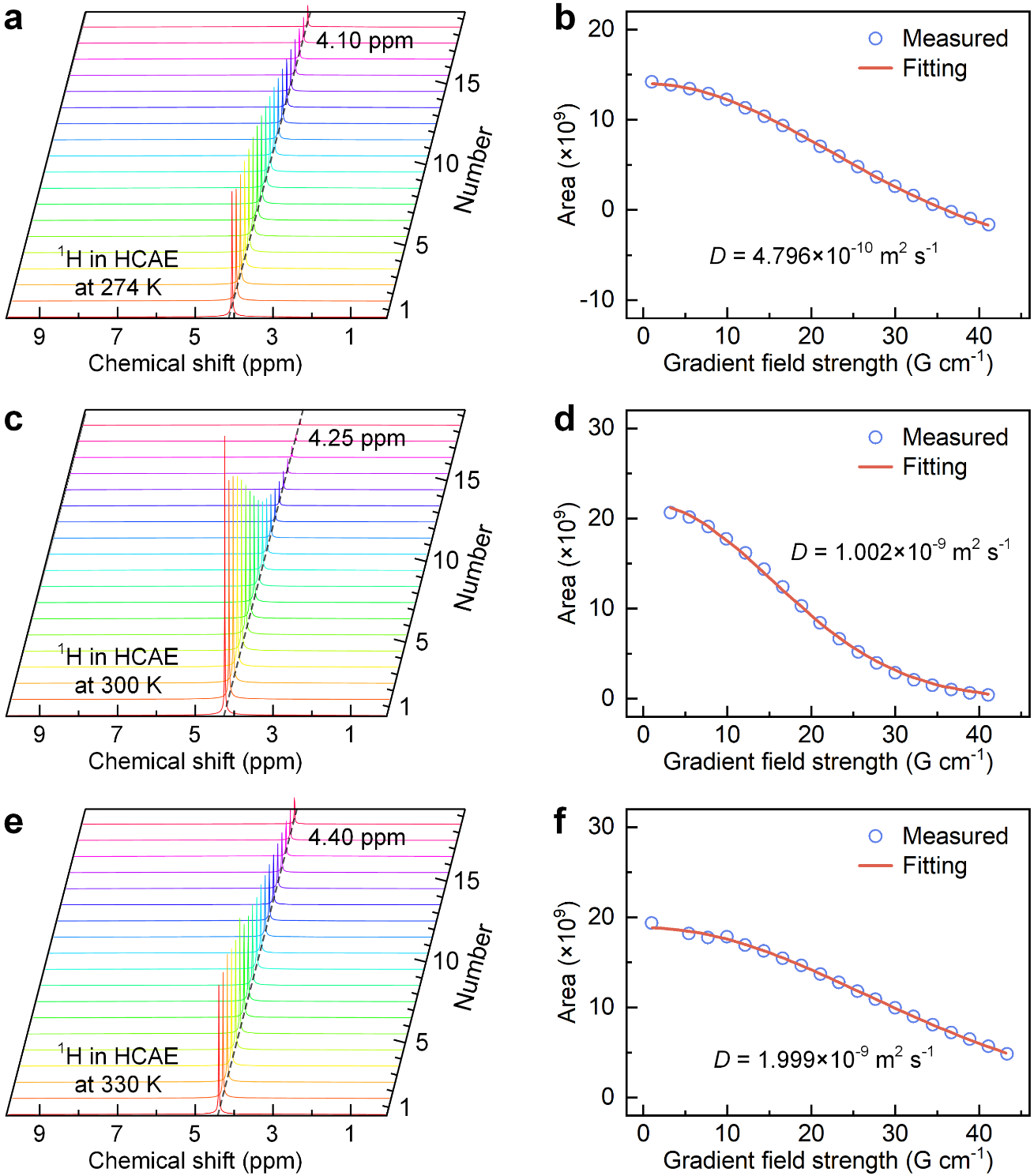


**Fig. S3** PFG-NMR results of ^1^H nucleus in bulk HCAE. **a–b** PFG-NMR spectrum and diffusion peak area at 274 K, respectively. **c–d** PFG-NMR spectrum and diffusion peak area at 300 K, respectively. **e–f** PFG-NMR spectrum and diffusion peak area at 330 K, respectively. In (b), (d), and (f), blue points show the measured data, and the red line is a non-linear fit. Panel (c) is reproduced from Fig. 3d in the main text

The peak position of chemical shift for HCAE at 300 K is at 4.25 ppm, exhibiting a negative movement compared with that for MCAE at 300 K (Fig. S2c). For HCAE, the peak position of the chemical shift shows a monotonically positive shift with increasing temperature, *i.e.*, from 4.10 ppm at 274 K to 4.40 ppm at 330 K. This phenomenon is attributed to the thermal-enhanced electron density around H atoms in water molecules, consistent with the temperature-dependent linear relationship in a previous report [S19].


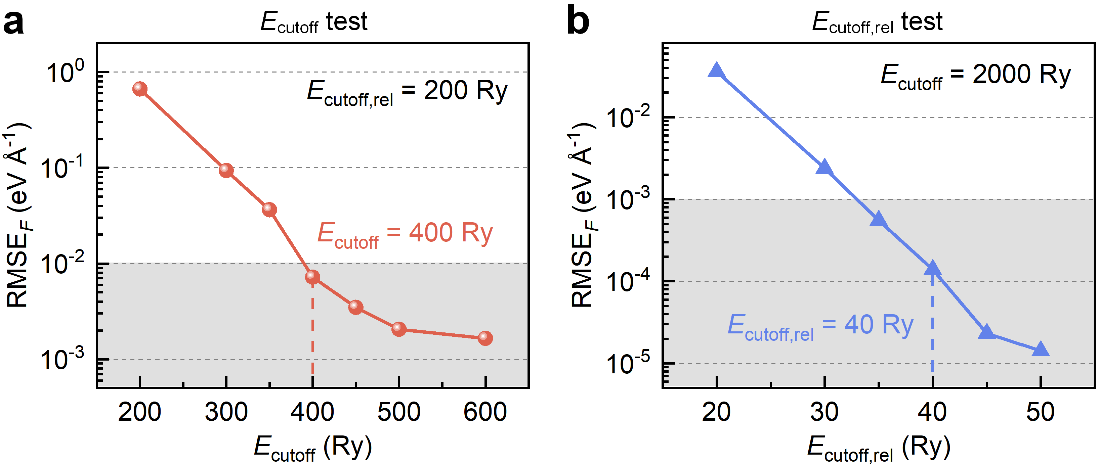


**Fig. S4** Root mean square error (RMSE) on atomic force in convergence tests. **a** Absolute energy cutoff *E*_cutoff_. **b** Relative energy cutoff *E*_cutoff,rel_. According to the test results, *E*_cutoff_ of 400 Ry and *E*_cutoff,rel_ of 40 Ry are adopted for simulations, respectively.


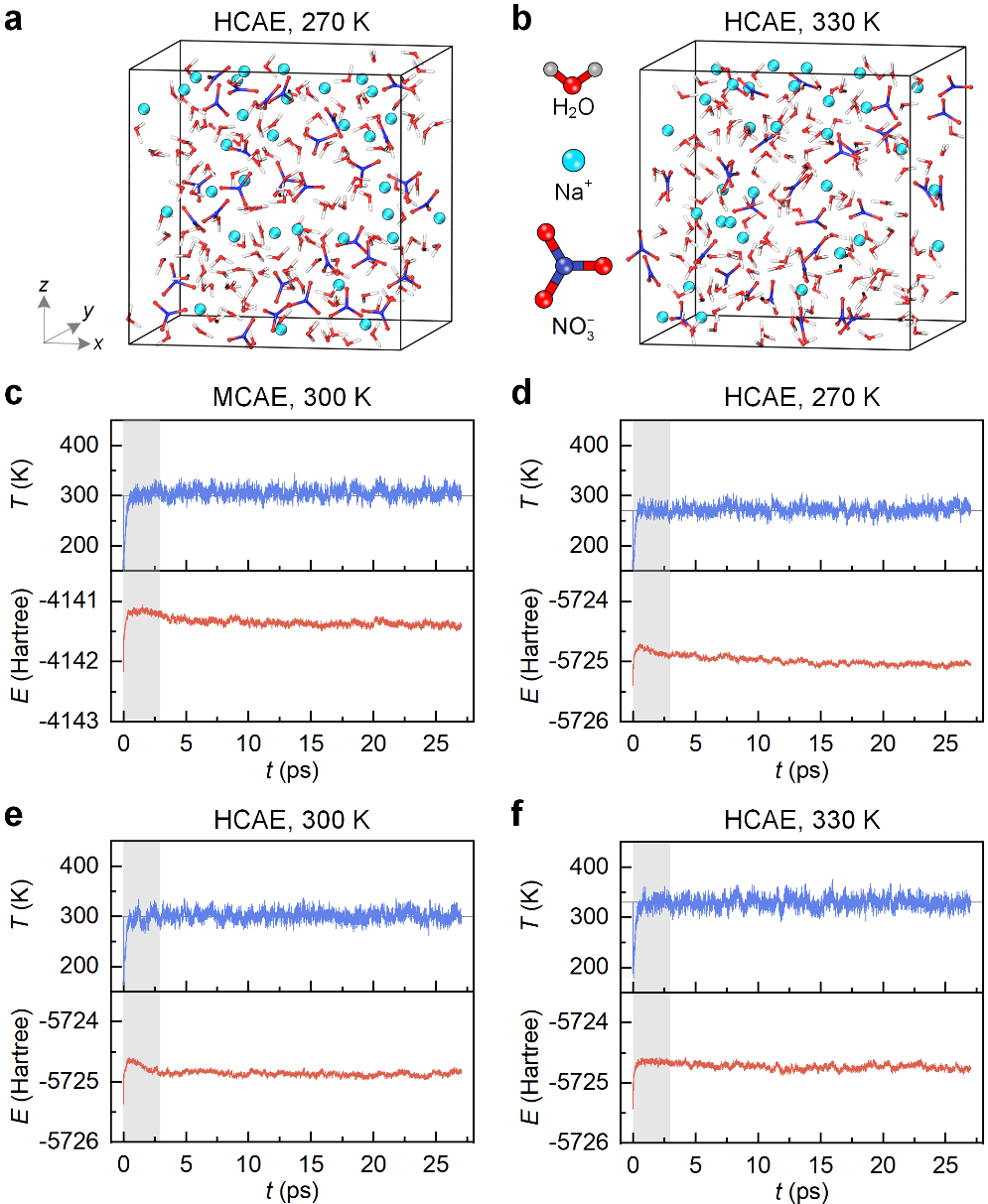


**Fig. S5** Snapshots and thermal equilibrium in FPMD simulations for bulk aqueous electrolytes. **a–b** Snapshots of HCAE at 270 and 330 K, respectively. **c–f** Time evolutions of temperature and energy of MCAE at 300 K, HCAE at 270 K, HCAE at 300 K, and HCAE at 330 K, respectively. Grey regions represent the 3-ps equilibrium periods. Time intervals from 3 to 27 ps denote the production periods, during which the temperature and energy slightly fluctuate around the target values. The systems thus achieve thermal equilibrium for a sufficiently long period.


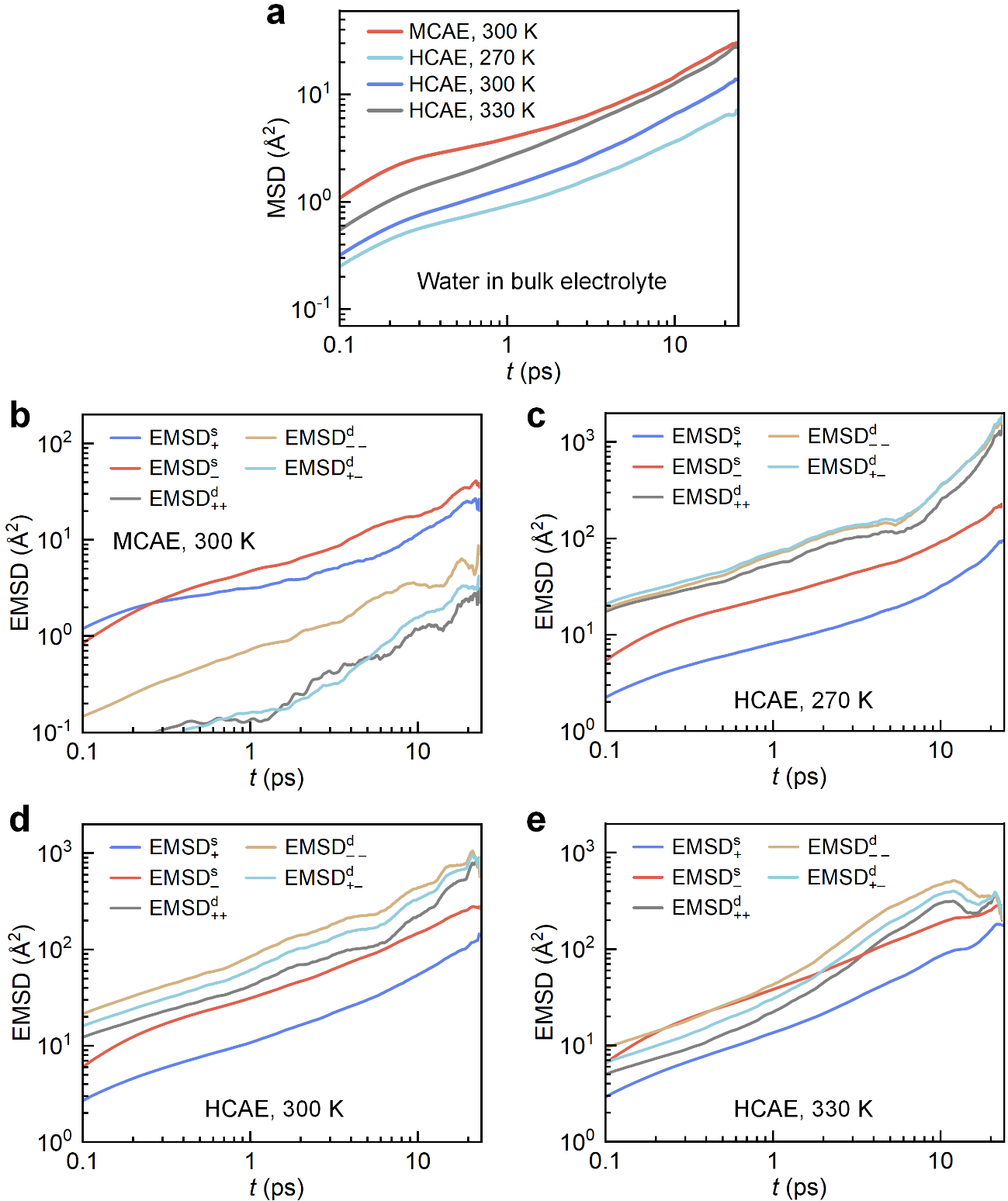


**Fig. S6** Mean square displacement (MSD) and effective MSD (EMSD) of bulk aqueous electrolytes. **a** Log-log plot of water MSD versus time *t*. **b–e** Log-log plot of ion EMSD versus time *t* of MCAE at 300 K, HCAE at 270 K, HCAE at 300 K, and HCAE at 330 K, respectively


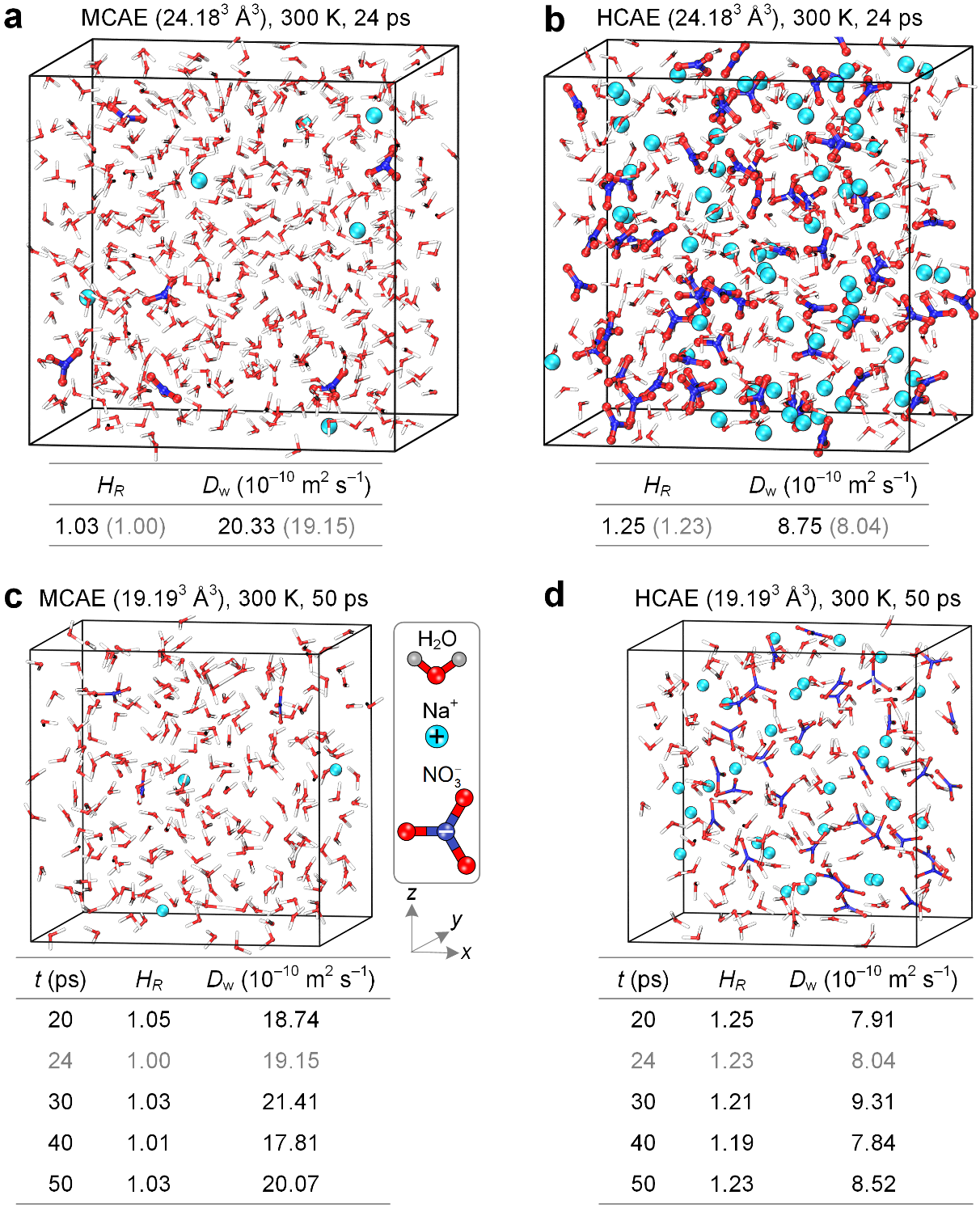


**Fig. S7** Simulation results upon the large model size and long sampling time. **a–b** Simulation snapshots and transport outputs of bulk MCAE and HCAE with a large model size (24.18^3^ Å^3^), respectively. **c–d** Simulation snapshots and transport outputs of bulk MCAE and HCAE with long sampling time up to 50 ps, respectively. In panels (c–d), snapshots are reproduced from Fig. 4d. In all panels, transport outputs include *H_R_* and water diffusivity *D*_w_. Gray texts denote the results based on 19.19^3^ Å^3^ and 24 ps reported in the main text. Based on the results, *H_R_* and *D*_w_ upon large model sizes and long simulation durations are comparable to those upon 19.19^3^ Å^3^ and 24 ps, with differences below 10%. The transport performance reported in the main text thus achieves satisfactory convergence with respect to model size and sampling time


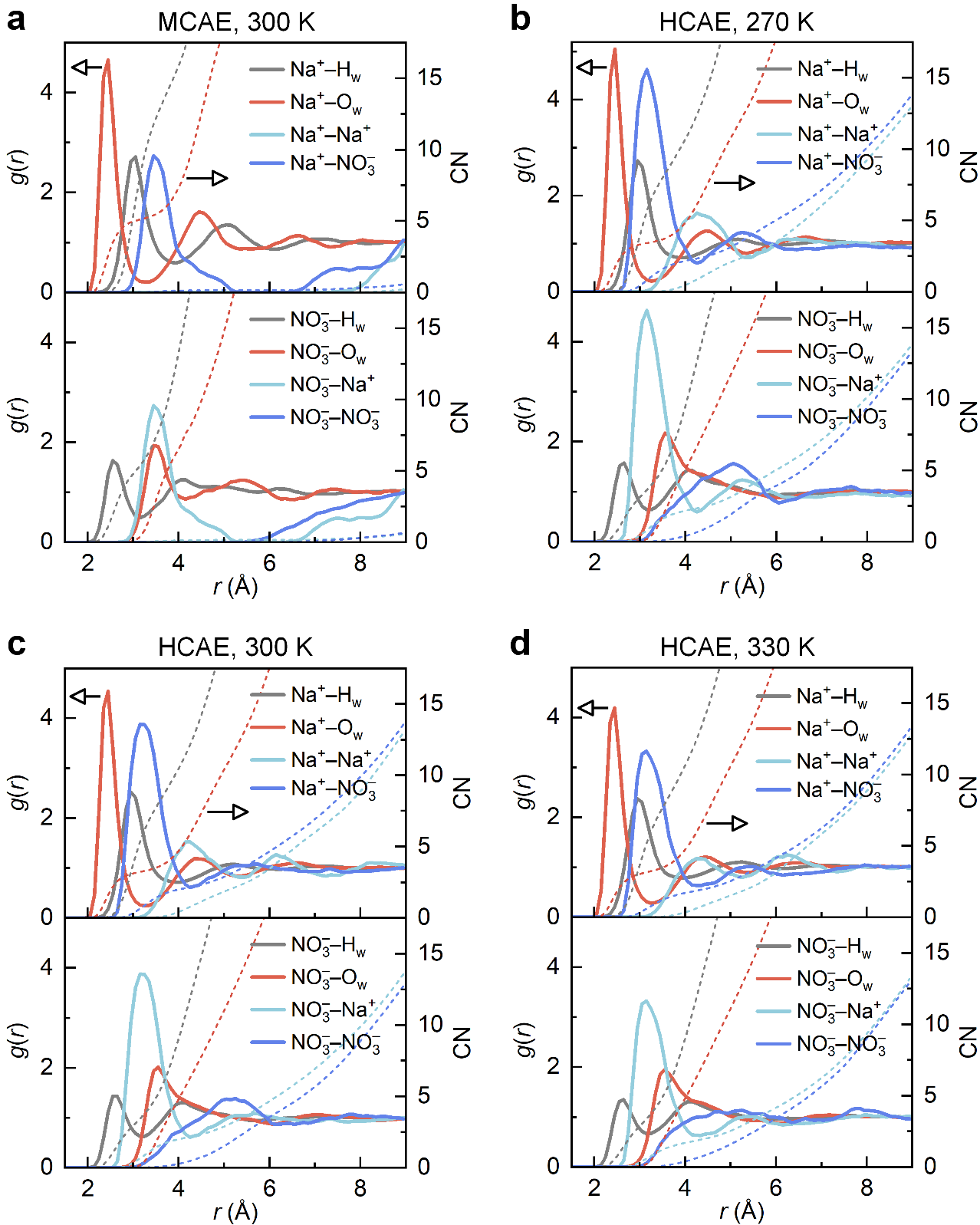


**Fig. S8** Radial distribution functions *g*(*r*) for bulk aqueous electrolytes under concentration and temperature regulations. **a–d** MCAE at 300 K, HCAE at 270 K, HCAE at 300 K, and HCAE at 330 K, respectively. Solid lines denote *g*(*r*) on the left Y-axis and dashed lines denote coordination number (CN) on the right Y-axis. O_w_ and H_w_ denote oxygen and hydrogen atoms in water molecules, respectively

**
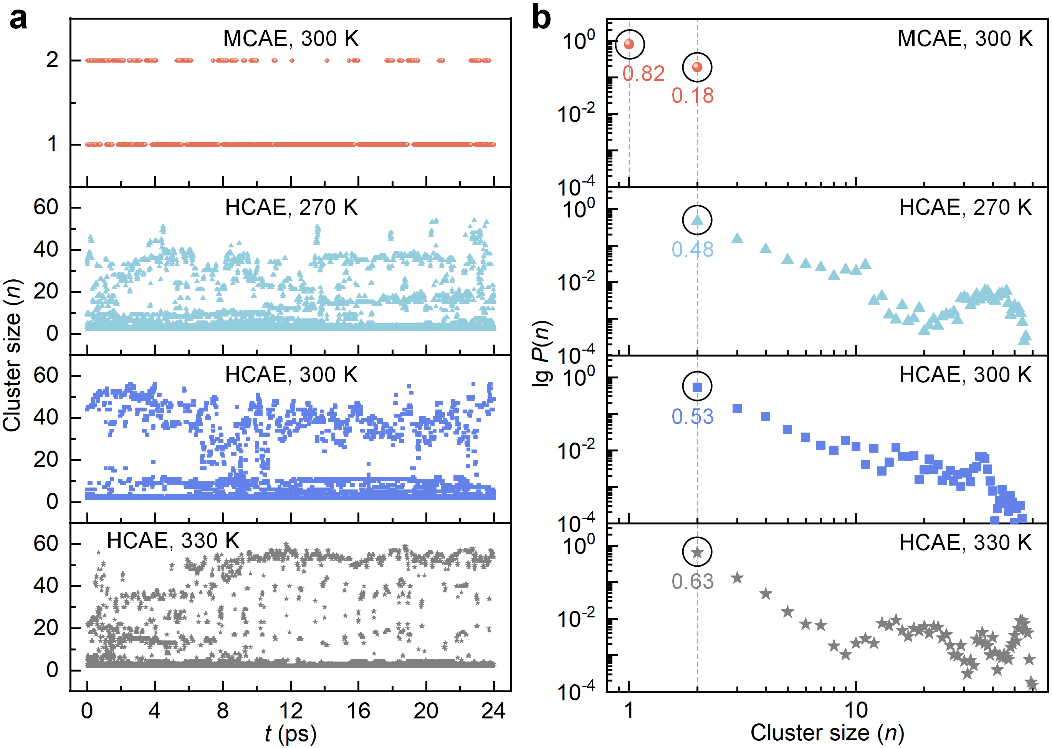
**

**Fig. S9** Cluster size analyses of ions in bulk MCAE and HCAE. **a** Time evolution of cluster size (*n*). **b** Probability distribution of cluster size. The distance criterion for determining ion state stems from the first RDF(Na^+^–NO_3_^–^) valley (*i.e.*, 4.2 Å) shown in Fig. S8. The cluster size of one denotes an isolated ion, the size of two denotes a small ion pair, and the larger sizes represent aggregates consisting of multiple cations and anions

In panel (a), the ion cluster size always changes between one and two for MCAE, and the size of one is dominant over time. Hence, ions are primarily isolated and less connected in the MCAE system. In contrast, multiple ion clusters with distinct sizes generally coexist at each moment in HCAEs, implying the diversity of ion cluster types.

In panel (b), the probabilities of isolated and ion-pair states are 0.82 and 0.18 in MCAE, respectively. In contrast, the isolated ion is absent, and the small ion pair is dominant in HCAEs. This result is consistent with the structure-averaged outputs shown in Fig. 5c. As temperature rises from 270 to 300 and 330 K in HCAEs, the probability of small ion pairs is enlarged from 0.48 to 0.53 and 0.63, corresponding to fewer ion aggregates with large sizes. Therefore, thermal effects can weaken ion–ion correlation and reduce the large-sized ion aggregates, in line with the results shown in Fig. 6g.


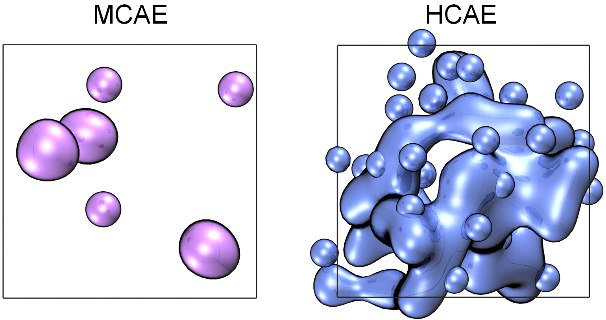


**Fig. S10** Topological structures of ions in bulk MCAE (purple) and HCAE (light blue). The two snapshots are identical to those in Fig. 5d. The dissolved ions are less connected in MCAE, whereas the large-size ion clusters and network-like ion aggregates are formed in HCAE.


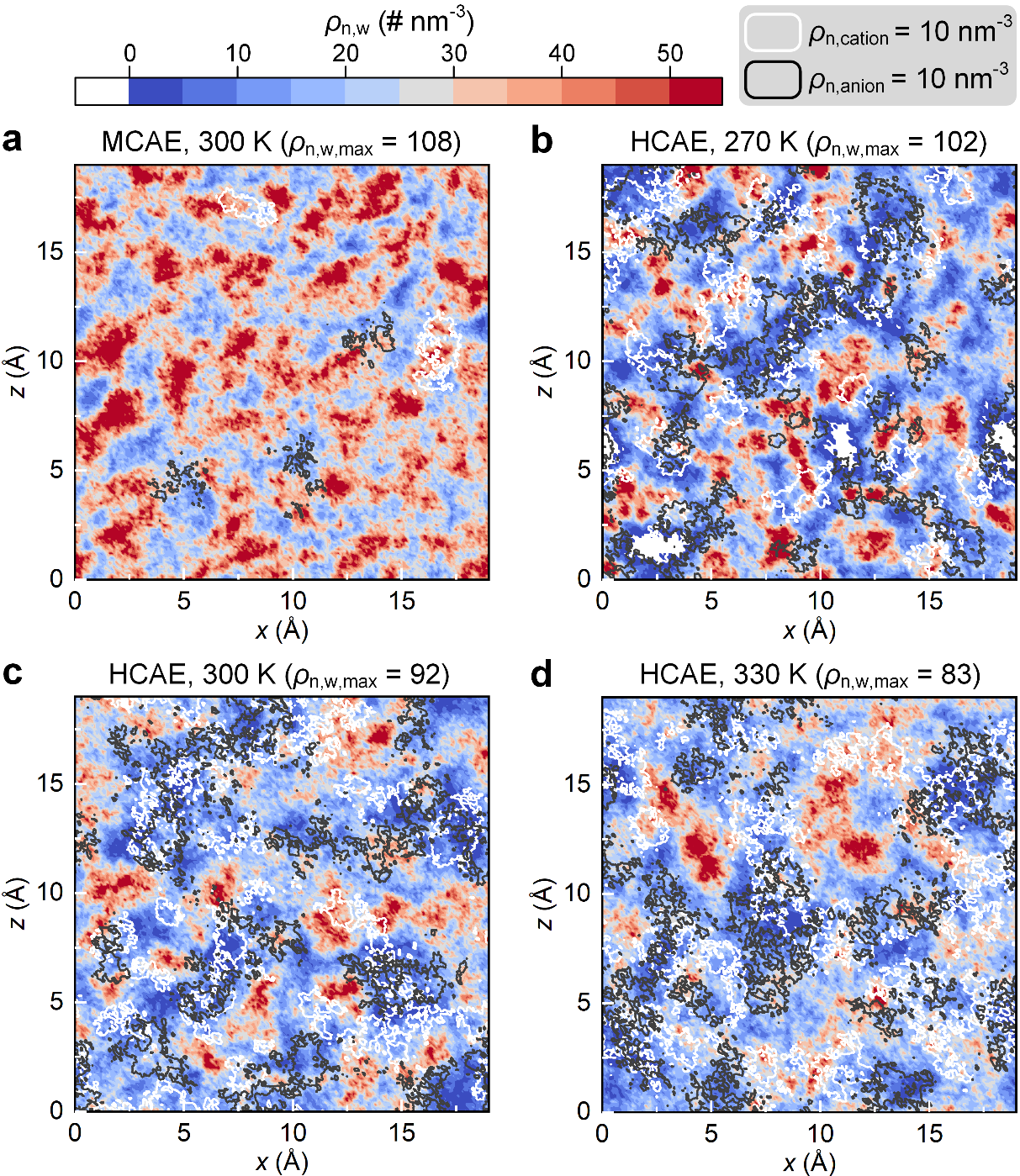


**Fig. S11** Atomic number density map of constituents in bulk aqueous electrolytes. **a–d** MCAE at 300 K, HCAE at 270 K, HCAE at 300 K, and HCAE at 330 K, respectively. Atomic number density of water molecule is shown by the colormap. Atomic number densities of cation and anion are shown by white and black contours with the number density of 10 nm^–3^, respectively.

In panel (a), water molecules show high-density spots with uniform distributions, whereas the high-density regions of cation and anion are scattered and separated from each other. Ions in MCAE are thus separated by water molecules. In panel (b), water molecules show a remarkable heterogeneous distribution within the entire electrolyte space. Certain regions are occupied by high-concentration water molecules, whereas other regions are water-poor and even water-less. Meanwhile, cations and anions are mainly found in water-poor areas, and they are close to each other and even overlap. Hence, HCAE presents aggregate patterns and structured distributions at low temperatures. In panels (c–d), the high-density water spots decrease sequentially, and ions tend to be widely distributed throughout the space. Thermal effects thus disintegrate aggregates in HCAE, and the constituents tend to show homogeneous distributions.


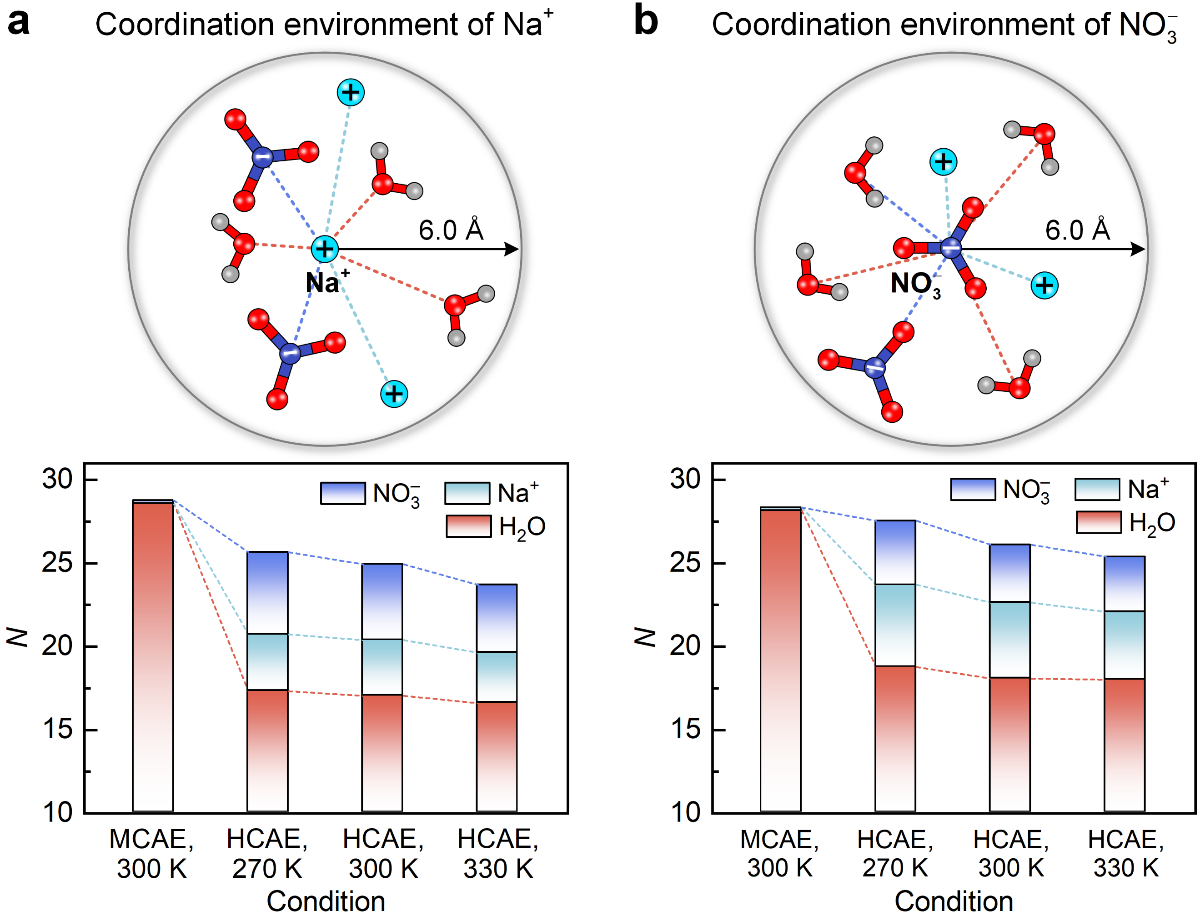


**Fig. S12** Ion coordination environment in bulk aqueous electrolytes. **a** Cation. **b** Anion. The number (*N*) of constituents here is extracted from the coordination number (CN) shown in Fig. S8. The radial distance of 6.0 Å is chosen since constituents have already reached at least the first *g*(*r*) peak before this distance, as shown in Fig. S8.

In panel (a), cation coordination environment for MCAE at 300 K contains the highest total number of constituents, in which water molecules are the overwhelming majority. As the concentration increases to HCAE, water molecules in cation coordination environment decrease significantly but are still more than anion, with cation being the least abundant. Hence, compared with MCAE, cation coordination environment in HCAE involves the remarkable participation of ion constituents, resulting in the formation of aggregates. Anions are even more than cations around the central cation, in line with the results of Fig. 6b in the main text. As the temperature increases from 270 K to 330 K, water molecules and ions in the cation coordination environment monotonically decrease. Hence, thermal effects alter the component proportion in the aggregates and reduce the strength of ion–ion correlations, in line with the results of Fig. 6g in the main text. The same conclusions can be obtained from the anion coordination environment in panel (b).

**
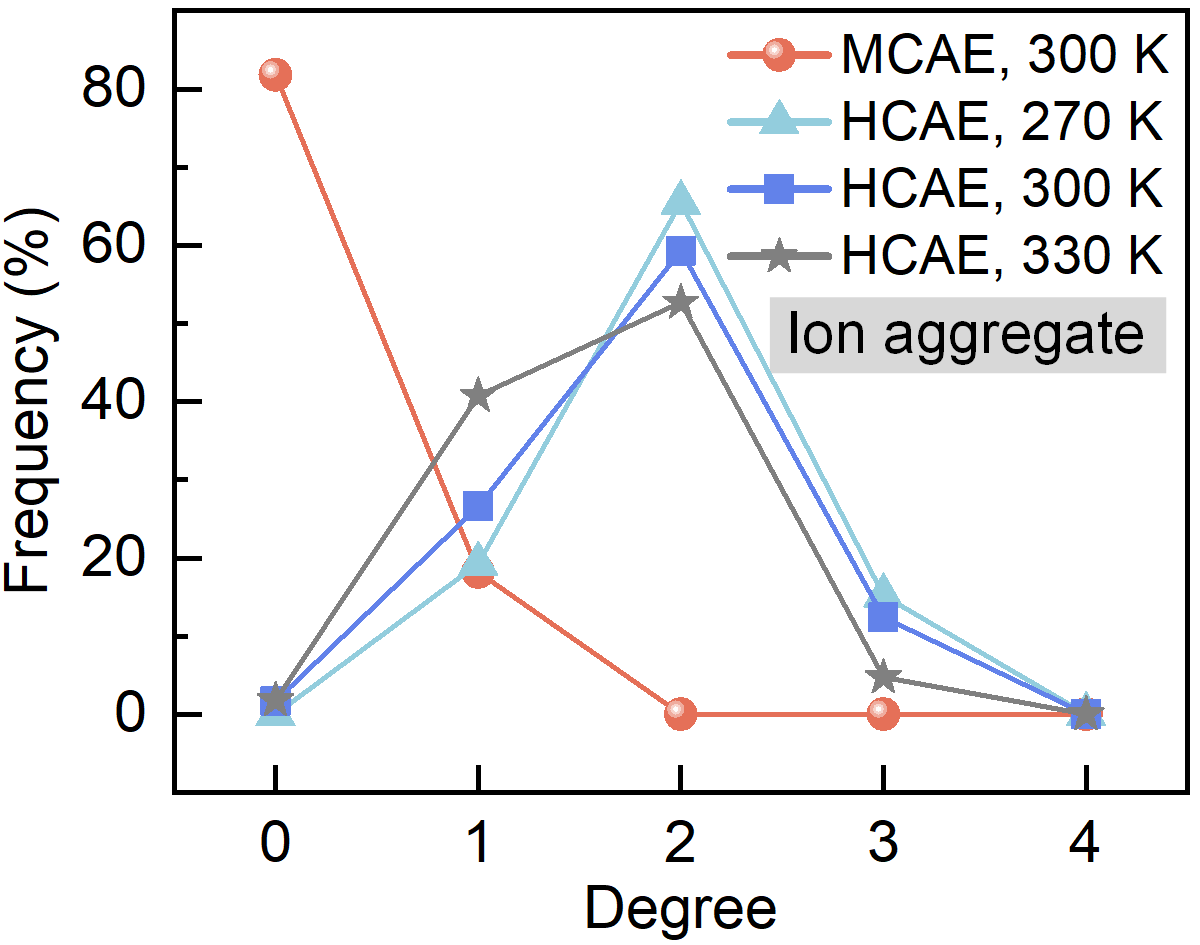
**

**Fig. S13** Degree distribution of ion aggregate in bulk aqueous electrolytes

In terms of ion aggregate, the essence of degree distribution is a frequency distribution of NO_3_^–^ ion number around a referenced Na^+^ ion, where the position of the first RDF(Na^+^–NO_3_^–^) valley (*i.e.*, 4.2 Å, Fig. S8) serves as a distance criterion to determine Na^+^–NO_3_^–^ edges. The distribution is primarily at the degree of zero for MCAE at 300 K, implying less correlation among ions. In contrast, the average degree is higher (approximately two) in HCAE at 300 K, suggesting that each Na^+^ is surrounded by two counterions. Ion aggregates are thus formed in HCAE. The distribution for HCAE even moves to a low degree under thermal effects, implying the weakened ion aggregates and thus the promoted ion transport in HCAE.


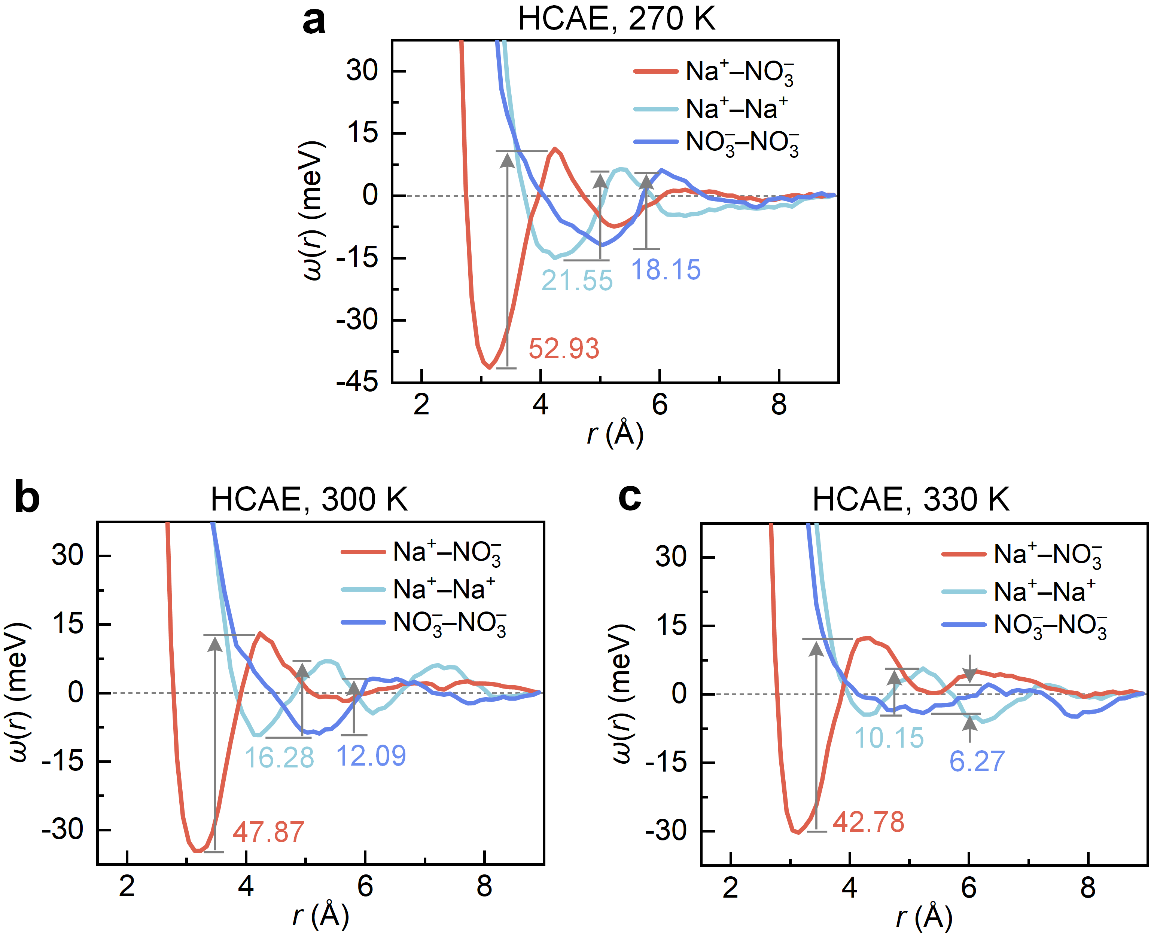


**Fig. S14** Potential of mean force for ion pair dissociation in HCAE in temperature-regulated bulk states. **a** 270 K. **b** 300 K. **c** 330 K

In panel (a), the energy barrier required to be overcome is 52.93 meV for HCAE at 270 K for dissociating an anion from the first coordination shell of central cation, implying a crowded ion arrangement in HCAE. This barrier is prominently higher than that for dissociating a cation from the central cation (21.55 meV) and for dissociating an anion from the central anion (18.15 meV). In panels (b–c), all energy barriers decrease monotonically, suggesting that thermal effects contribute to the disintegration of ion–ion correlations.


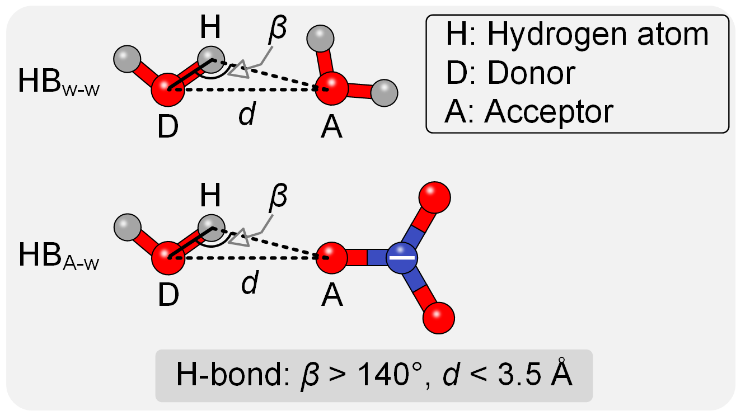


**Fig. S15** Geometric criterion of H-bonds. H-bonds are formed when the donor-acceptor distance is less than 3.5 Å and the donor-H-acceptor angle exceeds 140°. HB_w-w_ and HB_A-w_ represent water-water and anion H-bonds, respectively.


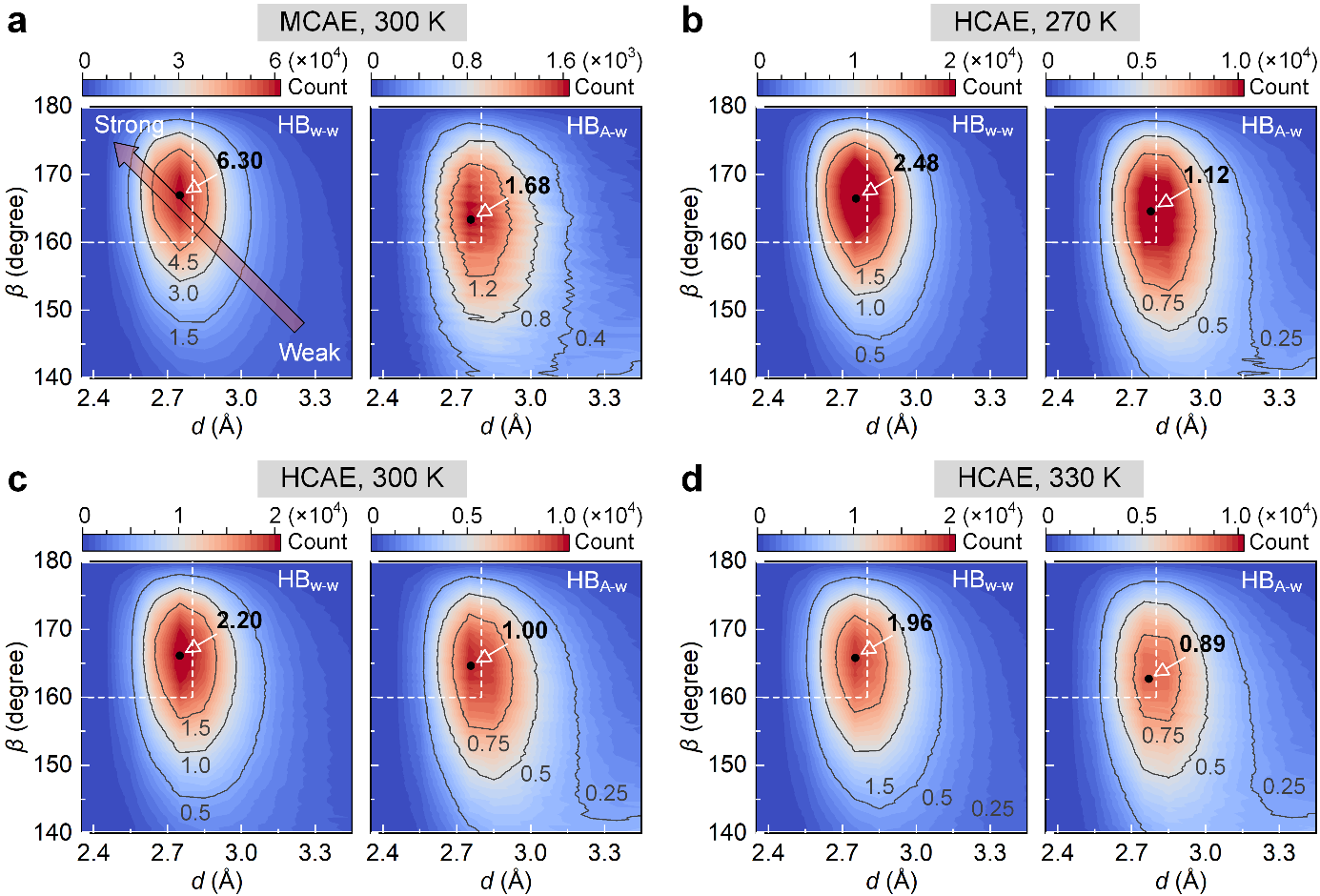


**Fig. S16** H-bond length–angle colormap for bulk aqueous electrolytes. **a** MCAE at 300 K. **b** HCAE at 270 K. **c** HCAE at 300 K. **d** HCAE at 330 K. In each panel, HB_w-w_ and HB_A-w_ denote water-water and anion-water H-bonds, respectively. H-bond length is the donor-acceptor distance (denoted as *d*, < 3.5 Å), and H-bond angle is the donor-H-acceptor angle (denoted as *β*, > 140°). The left top corner suggests strong H-bonds with a short length (*i.e.*, small *d* value) and linear geometry (*i.e.*, *β* near 180°), and the right bottom corner corresponds to weak H-bonds. The length–angle configuration with the highest count is marked by a black spot.

In terms of bulk MCAE (Fig. S16a), the high-count area of HB_w-w_ is concentrated at the left top corner, and its (*d*, *β*) coordinate with the highest count of 6.30 × 10^4^ is located at (2.75 Å, 167°). In contrast, the high-count area of HB_A-w_ disperses towards the right bottom corner to a certain extent, and its (*d*, *β*) coordinate with the highest count of 1.68 × 10^3^ is located at (2.75 Å, 163°). Hence, according to the colormap pattern, the highest count, and the preferred location, HB_A-w_ is weaker and fewer compared with HB_w-w_ in bulk MCAE.

By comparing panels (a) and (c), as the system shifts from MCAE to HCAE with the same simulation duration, the highest count of HB_w-w_ is dramatically reduced to 2.20 × 10^4^, and the highest count of HB_A-w_ is enlarged by an order of magnitude to 1.00 × 10^4^. The numbers of strong HB_w-w_ and weak HB_A-w_ are thus reduced and enlarged, respectively. This phenomenon verifies the weakened H-bonds without fully breaking H-bond networks as ion concentration increases, explaining the concentration-dependent shift of water frequency in IR spectra (Fig. 3b).

By comparing panels (b–d), with a raised temperature, the highest counts of strong HB_w-w_ and weak HB_A-w_ are slightly reduced without a change in order of magnitude, in line with the results of the normalized H-bond number (Fig. 6h in the main text). Meanwhile, the colormap patterns and the preferred locations are less changed with temperature, explaining the constant water frequency in IR spectra under thermal effects (Fig. 3b).


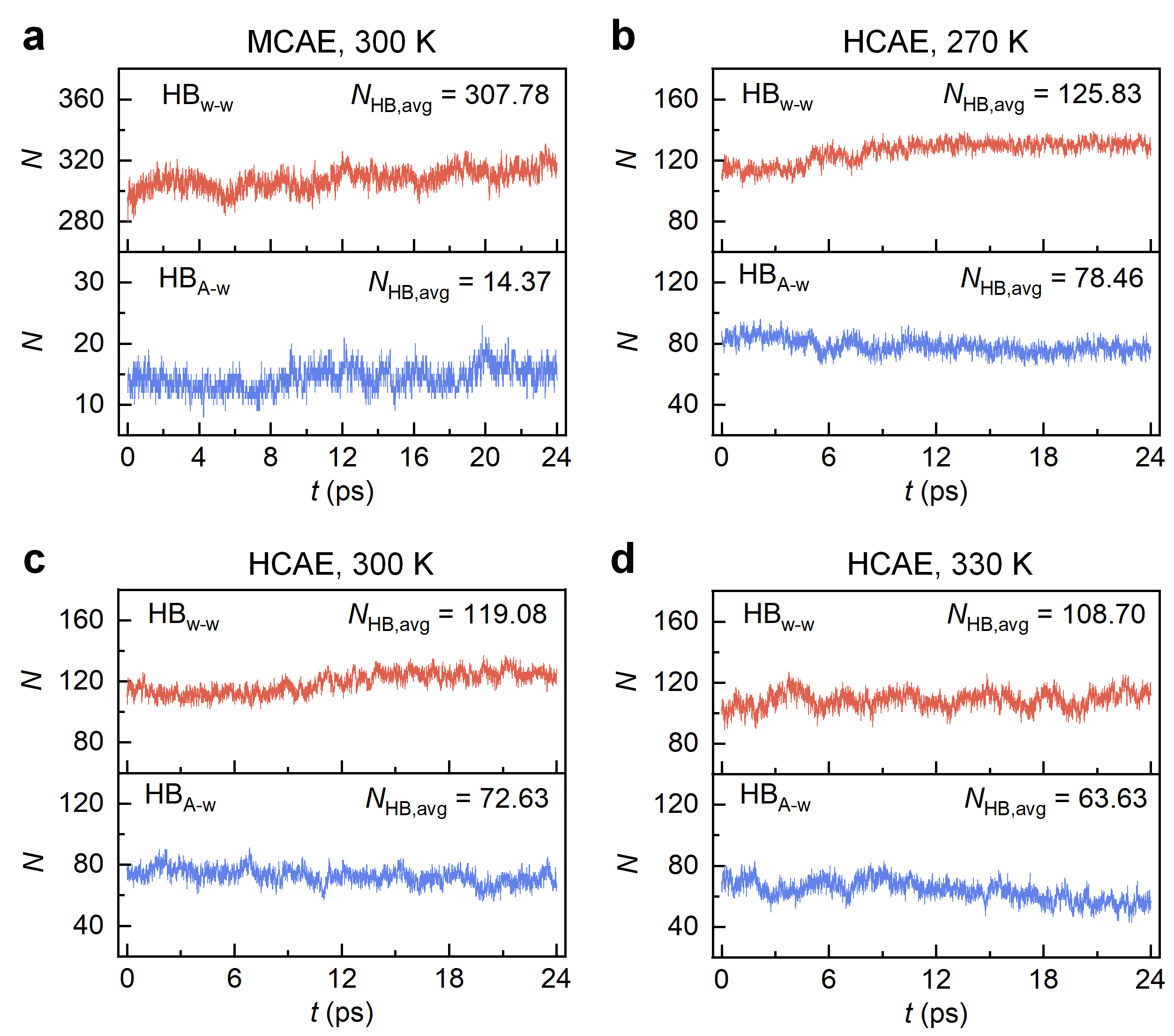


**Fig. S17** Time-evolved number of H-bonds in bulk aqueous electrolytes. **a** MCAE at 300 K. **b** HCAE at 270 K. **c** HCAE at 300 K. **d** HCAE at 330 K. HB_w-w_ and HB_A-w_ are water-water and anion-water H-bonds, respectively

In panel (a), the average number of HB_w-w_ (307.78) is notably higher than that of HB_A-w_ (14.37) for MCAE at 300 K, which is attributed to solvent water far more than ions in MCAE. As the concentration increases to HCAE, as shown in panels (b–d), the average number of HB_w-w_ and HB_A-w_ present notable reduction and enlargement, respectively, implying the weakened H-bond network connectivity among water molecules. In addition, the number of both H-bonds decreases slightly with temperature, indicating the thermally perturbed H-bond networks.


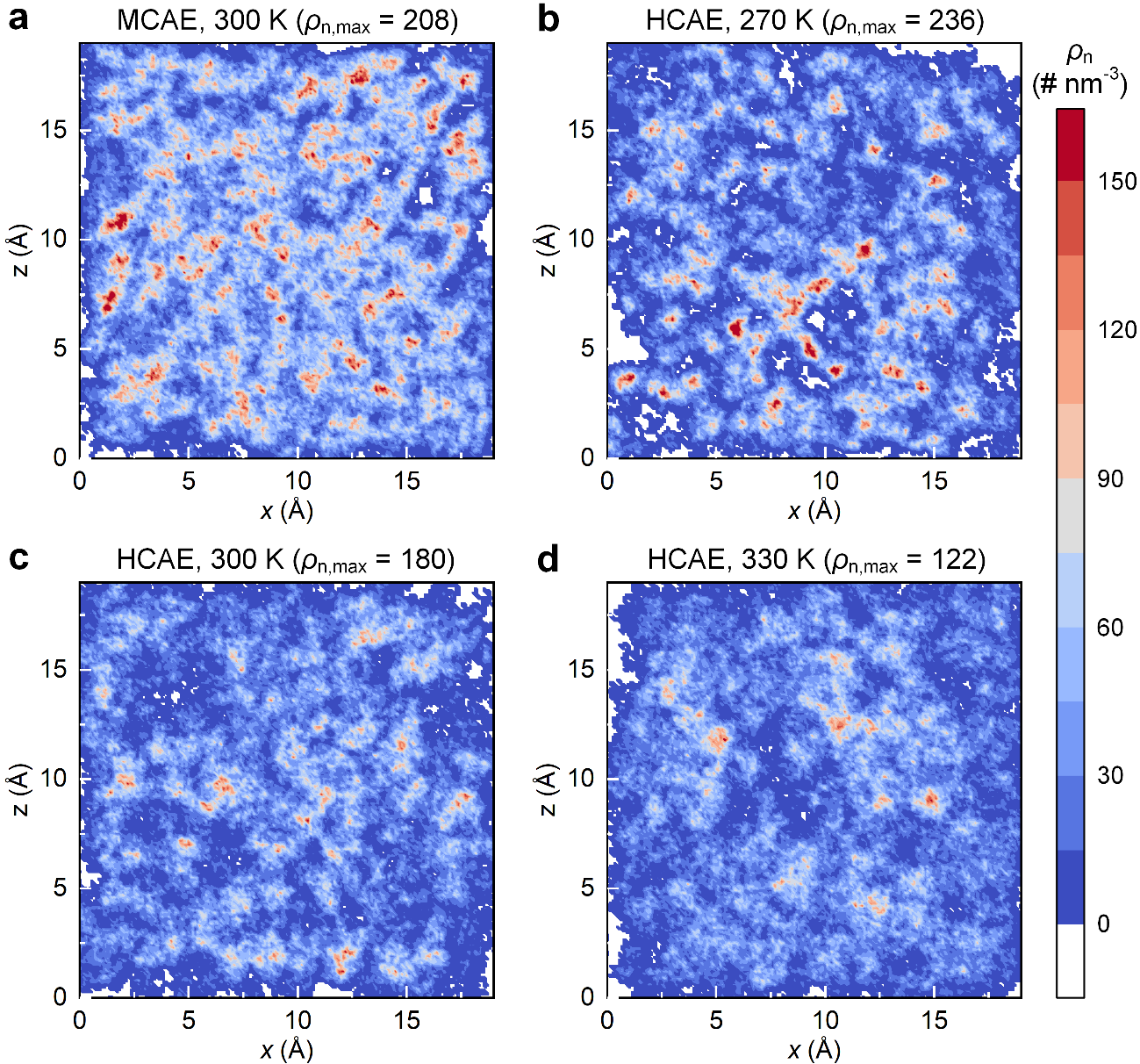


**Fig. S18** Density map of H-bonds in bulk aqueous electrolytes. **a** MCAE at 300 K. **b** HCAE at 270 K. **c** HCAE at 300 K. **d** HCAE at 330 K

In panel (a), H-bond networks present uniform distributions with numerous high-density spots for MCAE at 300 K. As the concentration increases to HCAE, as shown in panels (b–d), high-density spots significantly decrease, implying the weakened H-bond networks. Moreover, H-bonds tend to disperse with a lower density as the temperature increases, suggesting a more homogeneous spatial distribution of H-bond networks owing to thermal effects.

**
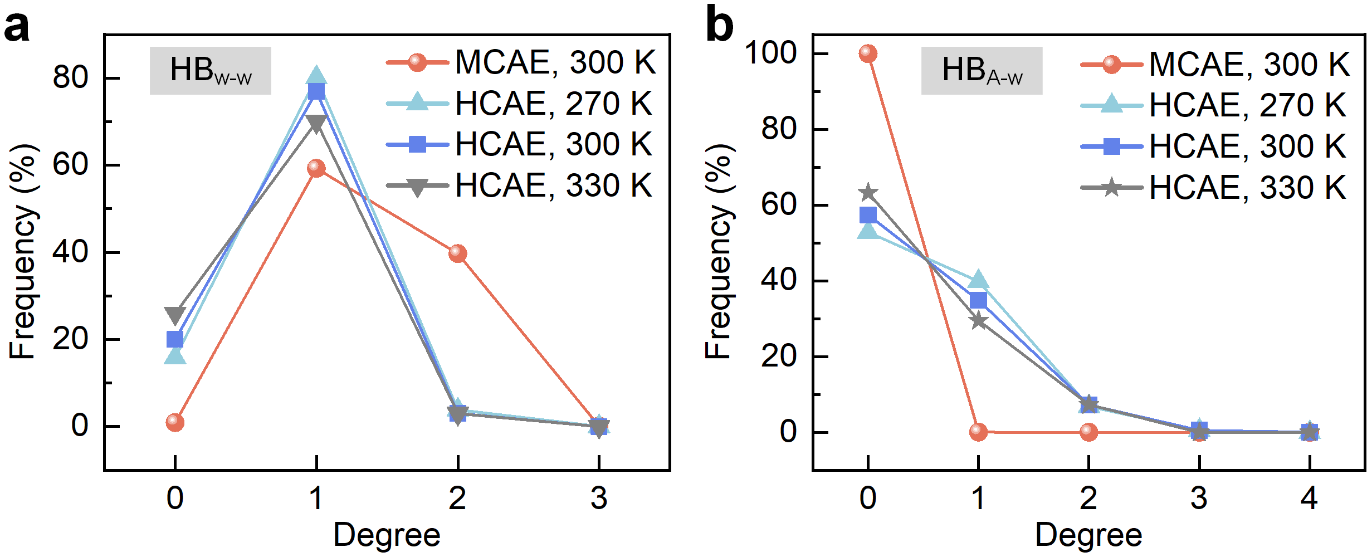
**

**Fig. S19** Degree distributions of H-bond networks in bulk MCAE and HCAE. **a–b** HB_w-w_ and HB_A-w_, respectively

In terms of H-bond networks, the essence of degree distribution is a frequency distribution of H-bond number (including HB_w-w_ and HB_A-w_) around a referenced water molecule. The H-bond criterion lies in a donor-acceptor distance less than 3.5 Å and a donor-H-acceptor angle larger than 140°. For HB_w-w_, the distribution is primarily within degree ranges of 1–2 and 0–1 for MCAE and HCAE at 300 K, respectively. For HB_A-w_, the dominant distribution is at the degree of 0 and within the degree range of 0–1 for MCAE and HCAE at 300 K, respectively. Moreover, the distributions related to both HB_w-w_ and HB_A-w_ for HCAEs tend to move toward a low degree under thermal effects. Hence, HCAE exhibits less connected H-bond networks compared with MCAE, and it presents fewer H-bonds due to thermal effects.


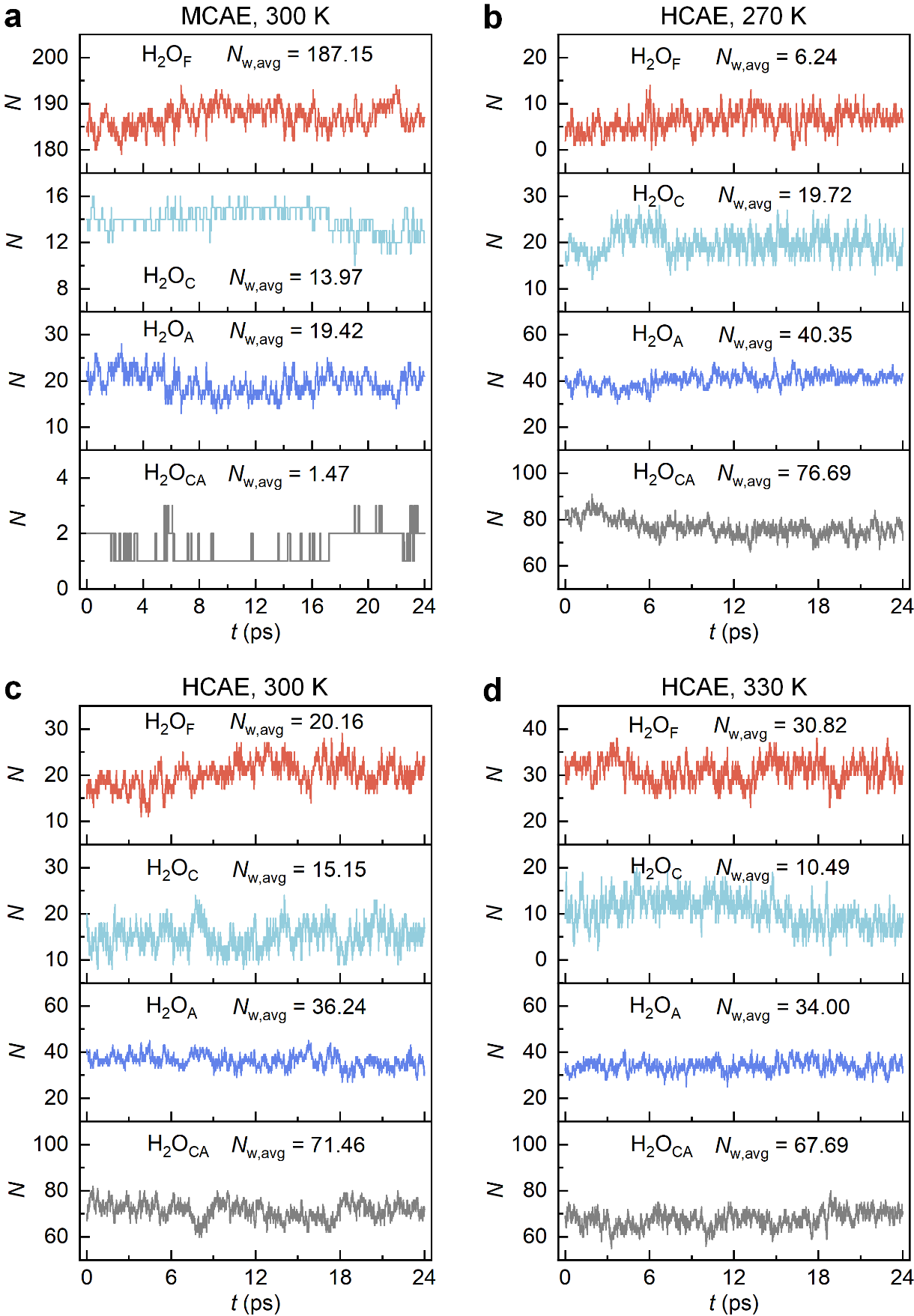


**Fig. S20** Time-evolved number of water molecules in bulk aqueous electrolytes. **a–d** MCAE at 300 K, HCAE at 270 K, HCAE at 300 K, and HCAE at 330 K, respectively. H_2_O_F_, H_2_O_C_, H_2_O_A_, and H_2_O_CA_ denote water molecules in a free state, solely solvated by cation, solely solvated by anion, and co-solvated by cation and anion, respectively

In panel (a), the average number of H_2_O_F_ is notably greater than that of water molecules in other states for MCAE at 300 K, and the average number of H_2_O_CA_ is almost zero, indicating that water molecules are predominantly in the free state. As the concentration increases to HCAE, as shown in panels (b–d), the average numbers of H_2_O_F_ and H_2_O_CA_ significantly decrease and increase, respectively. Hence, most water molecules in HCAEs are solvated and strongly bound. In addition, the average numbers of H_2_O_F_ and H_2_O_CA_ present certain growth and reduction with temperature, respectively, indicating that thermal effects can restore the free-state water.

**
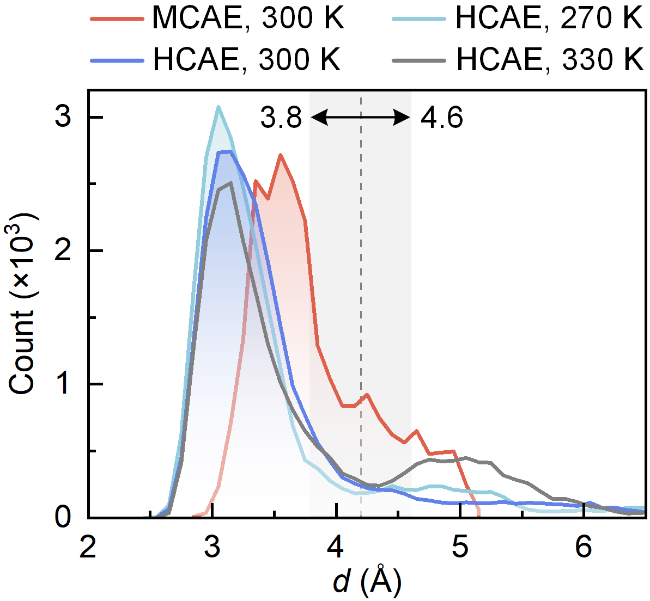
**

**Fig. S21** Probability distribution of the distance between a cation and its nearest-neighbor anion in bulk aqueous electrolytes. Gray dotted lines indicate the distance corresponding to the first RDF(Na^+^–NO_3_^–^) valley (*i.e.*, 4.2 Å, Fig. S8). Cation–anion distances less than 3.8 Å and larger than 4.6 Å imply a combination and separation of ion pairs, respectively. A portion of ion pairs exhibits distances between 3.8 and 4.6 Å, indicating the dynamic process of ion pair dissociation and combination. These phenomena suggest the ability to capture the dynamic evolution of ion pair patterns in bulk aqueous electrolytes in current simulations.

**
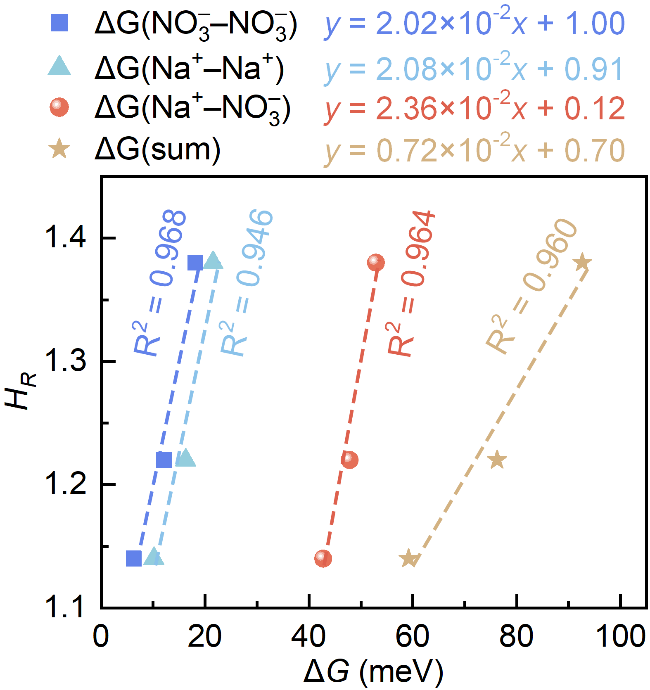
**

**Fig. S22** Quantitative relationship between the dissociation energy of ion pair (Δ*G*) and Haven ratio (*H_R_*) in bulk HCAEs. The *x*-axis involves Δ*G* values of ion pairs NO_3_^–^–NO_3_^–^, Na^+^–Na^+^, Na^+^–NO_3_^–^, and their sum (Fig. 6g in the main text). The accuracy of a linear relationship is evaluated by the coefficient of determination (R^2^).


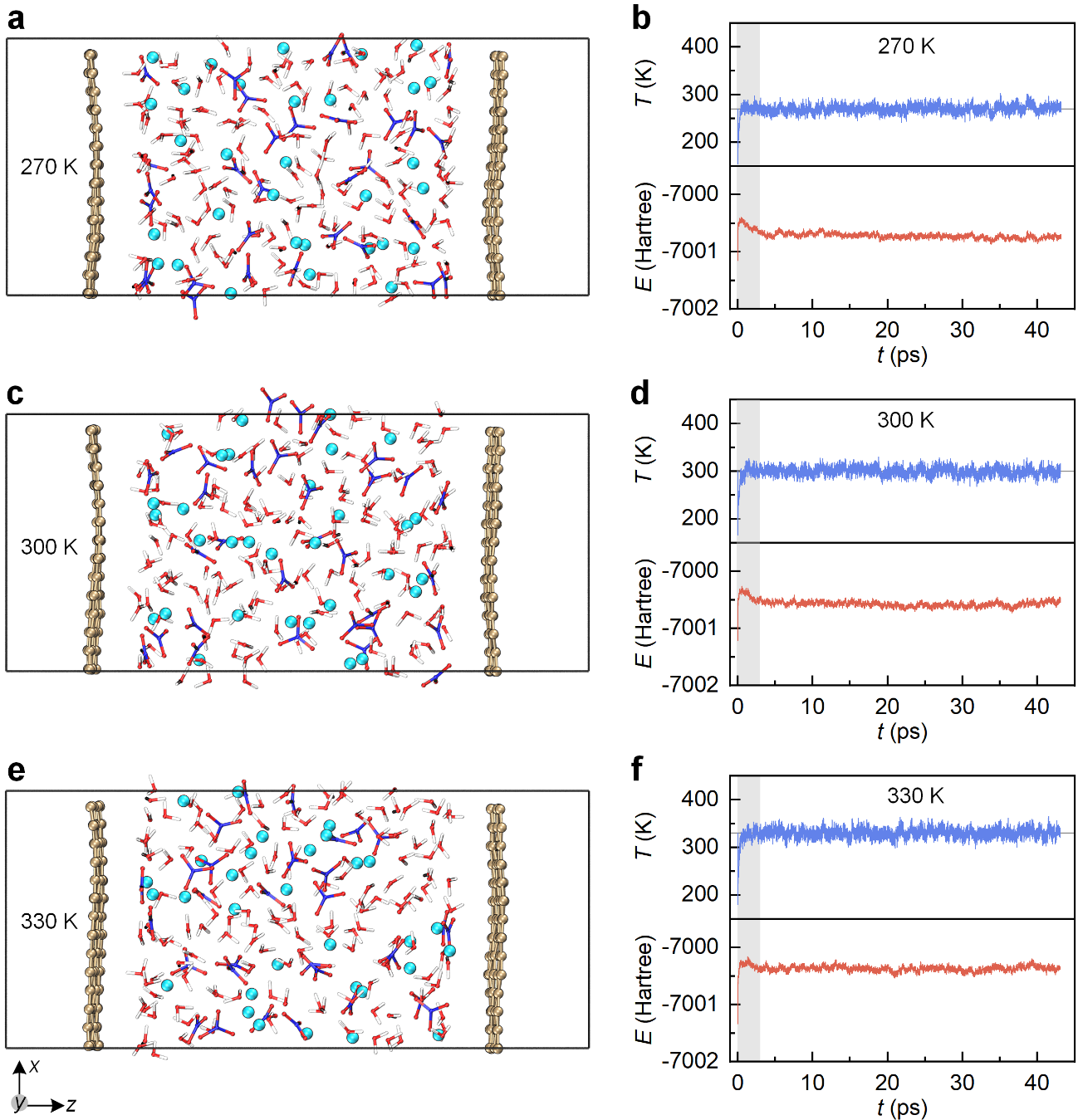


**Fig. S23** Snapshots and thermal equilibrium in FPMD simulations for HCAE at graphene interfaces under temperature regulations. **a–b** Snapshot as well as time-evolved temperature and energy at 270 K, respectively. **c–d** Snapshot as well as time-evolved temperature and energy at 300 K, respectively. **e–f** Snapshot as well as time-evolved temperature and energy at 330 K, respectively. Panel (c) is reproduced from Fig. 7a in the main text. In panels (b), (d), and (f), grey regions represent the 3-ps equilibrium periods, and time intervals from 3 to 43 ps denote the production periods, during which the temperature and energy slightly fluctuate around their target values. Hence, the systems realize thermal equilibrium for a sufficiently long period.


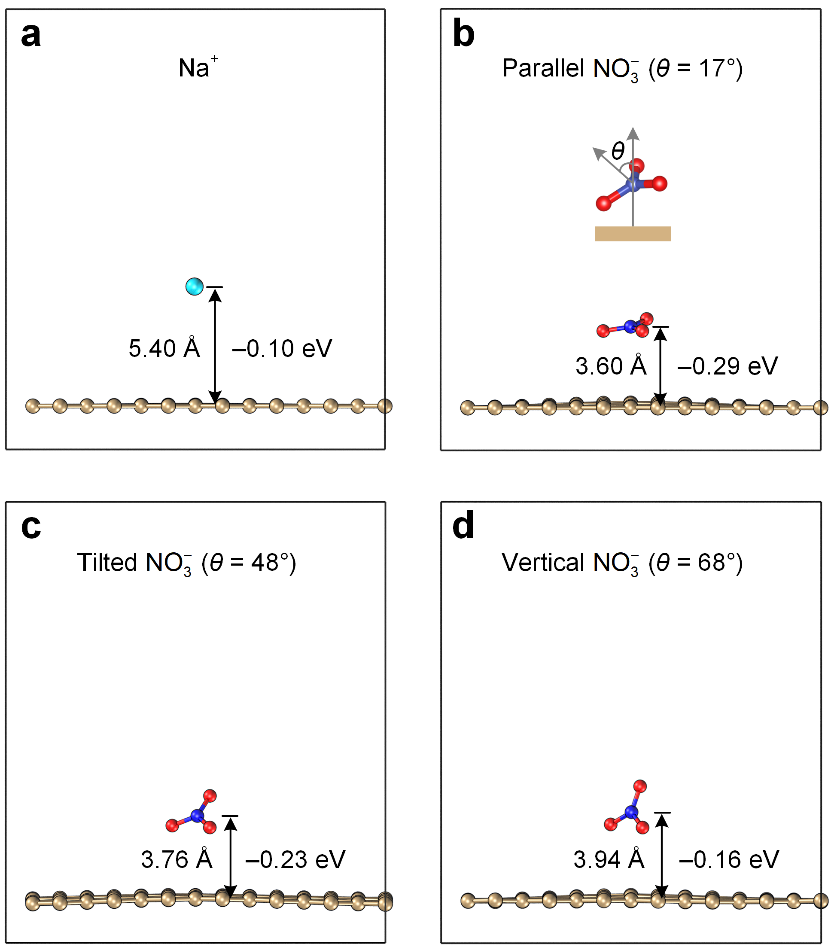


**Fig. S24** Adsorption distance and adsorption energy of cation and anion on the graphene surface. **a** Na^+^ ion. **b–d** NO_3_^–^ ion with parallel, tilted, and vertical orientations relative to the surface, respectively. All configurations are optimized to the local minimum value of energy. *θ* close to 0° denotes the parallel orientation, *θ* close to 90° denotes the vertical orientation, and other *θ* values denote the tilted orientation. NO_3_^–^ ion with parallel orientation can approach the graphene surface with a shorter distance and more negative adsorption energy.


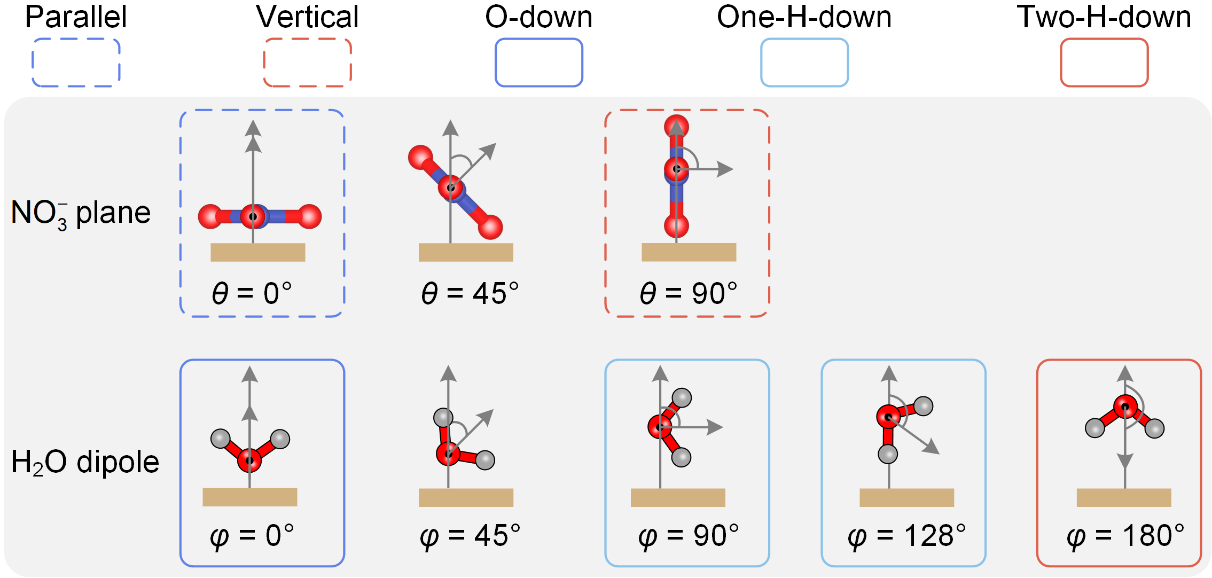


**Fig. S25** Schematic diagram of NO_3_^–^ ion and water orientations relative to the surface. *θ* is defined between the surface normal and the NO_3_^–^ plane normal. *θ* values near 0° and 90° denote parallel and vertical anion orientations, respectively. *φ* is defined between the surface normal and the opposite of water dipole. *φ* values close to 0°, 128°, and 180° represent O-down, one-H-down, and two-H-down water orientations, respectively.


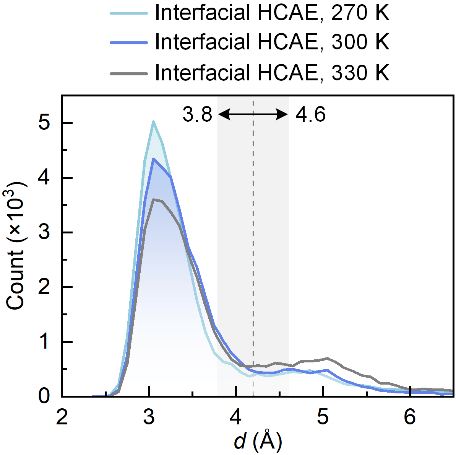


**Fig. S26** Probability distribution of the distance between a cation and its nearest-neighbor anion in interfacial HCAEs. Gray dotted lines indicate the distance corresponding to the first RDF(Na^+^–NO_3_^–^) valley (*i.e.*, 4.2 Å, Fig. S8). Cation–anion distances less than 3.8 Å and larger than 4.6 Å imply a combination and separation of ion pairs, respectively. A portion of ion pairs exhibits distances between 3.8 and 4.6 Å, indicating the dynamic process of ion pair dissociation and combination. These phenomena suggest the ability to capture the dynamic evolution of ion pair patterns in interfacial HCAEs in current simulations.


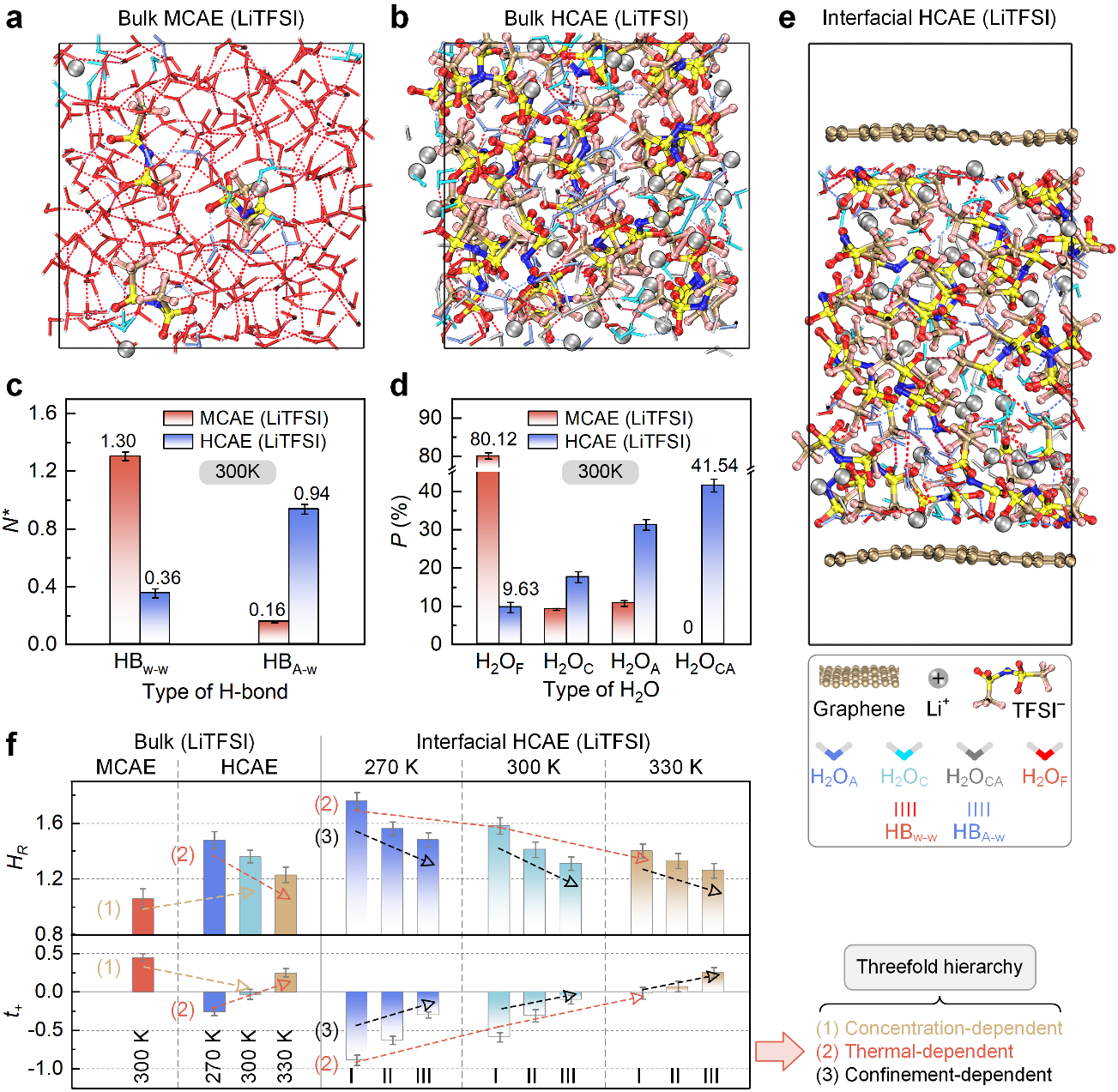


**Fig. S27** Threefold-hierarchical transport of LiTFSI aqueous electrolyte. **a–b** Ion correlation networks in bulk MCAE and HCAE, respectively. **c–d** Comparison between MCAE and HCAE in terms of normalized H-bond number and water proportion, respectively. **e** Ion correlation networks in the interfacial HCAE. **f** Threefold-hierarchical transport of LiTFSI electrolyte, as reflected by Haven ratio (*H_R_*) and cation transference number (*t*_+_). The error bars denote standard deviations.

## Supplementary Tables

**Table S1** FPMD simulation parameters used in this study. MCAE denotes the moderately concentrated aqueous electrolyte (0.71 M NaNO_3_), and HCAE denotes the highly concentrated aqueous electrolyte (7.29 M NaNO_3_)

| Simulation system | Bulk MCAE | Bulk HCAE | HCAE at interfaces |
| --- | --- | --- | --- |
| Number of species | 3 Na⁺, 3 NO_3_^–^, 222 H_2_O | 31 Na⁺, 31 NO_3_^–^, 143 H_2_O | 31 Na⁺, 31 NO_3_^–^, 143 H_2_O, 2 graphene sheets |
| Box size (Å) | 19.19 × 19.19 × 19.19 | 19.19 × 19.19 × 19.19 | 17.22 × 17.04 × 39.00 |
| Timestep (fs) | 1.0 | 1.0 | 1.0 |
| Ensemble | NVT | NVT | NVT |
| Simulation length (ps) | 24 | 24 | 40 |
| Temperature (K) | 300 | 270, 300, 330 | 270, 300, 330 |

**Table S2** Water diffusivity (*D*_w_, 10^–10^ m^2^ s^–1^) of bulk NaNO_3_ aqueous electrolytes obtained from PFG-NMR experiments and FPMD simulations. DAE, MCAE, and HCAE denote the diluted aqueous electrolyte (0.01 M NaNO_3_), moderately concentrated aqueous electrolyte (0.71 M NaNO_3_), and highly concentrated aqueous electrolyte (7.29 M NaNO_3_), respectively

|  | DAE | MCAE | | HCAE | |
| --- | --- | --- | --- | --- | --- |
| *T* (K) | PFG-NMR | PFG-NMR | FPMD | PFG-NMR | FPMD |
| 270 | – | – | – | 4.80 (at 274 K) | 4.65 |
| 285 | – | – | – | 6.82 | – |
| 300 | 23.41 | 21.81 | 19.15 | 10.02 | 8.04 |
| 315 | – | – | – | 16.31 | – |
| 330 | – | – | – | 19.99 | 17.46 |

**Table S3** Experimentally measured electrolyte conductivity (*σ*_exp_, mS cm^–1^) of bulk aqueous electrolytes. *σ*_exp_ of HCAE at 300 K is 191.37 mS cm^–1^, in line with the previous experimental result of 198 mS cm^–1^ measured at room temperature [S20]

| *T* (K) | DAE | MCAE | HCAE |
| --- | --- | --- | --- |
| 270 | 2.11 | 38.47 | 157.07 |
| 285 | 2.31 | 42.67 | 166.33 |
| 300 | 2.71 | 49.53 | 191.37 |
| 315 | 3.22 | 56.63 | 230.27 |
| 330 | 3.44 | 63.17 | 268.37 |

**Table S4** Deviation from the Nernst–Einstein relation of bulk aqueous electrolytes obtained from FPMD simulations. Ionic conductivity (*σ*) is provided with the unit of mS cm^–1^. Haven ratio (*H_R_*) and cation transference number (*t*_+_) are dimensionless

| Condition | $\sigma_{\text{GK}}$ | $\sigma_{\text{NE}}$ | $\sigma_{+}^{\text{s}}$ | $\sigma_{-}^{\text{s}}$ | $\sigma_{++}^{\text{d}}$ | $\sigma_{--}^{\text{d}}$ | $\sigma_{+-}^{\text{d}}$ | *H_R_* | *t_+_* |
| --- | --- | --- | --- | --- | --- | --- | --- | --- | --- |
| MCAE, 300 K | 50.19 | 50.31 | 20.27 | 30.04 | 1.63 | 3.87 | 2.81 | 1.00 | 0.38 |
| HCAE, 270 K | 152.53 | 212.26 | 62.33 | 149.93 | 702.03 | 885.4 | 823.58 | 1.39 | –0.39 |
| HCAE, 300 K | 200.83 | 247.56 | 80.39 | 167.17 | 517.93 | 712.36 | 638.51 | 1.23 | –0.20 |
| HCAE, 330 K | 267.85 | 308.58 | 117.63 | 190.95 | 485.51 | 559.98 | 543.11 | 1.15 | 0.22 |

**Table S5** Cation diffusivity (*D*_+_, 10^–10^ m^2^ s^–1^) and anion diffusivity (*D*_–_, 10^–10^ m^2^ s^–1^) in bulk aqueous electrolytes obtained from FPMD simulations

| Condition | *D*_+_ | *D*_–_ |
| --- | --- | --- |
| MCAE, 300 K | 7.76 | 11.63 |
| HCAE, 270 K | 2.29 | 5.51 |
| HCAE, 300 K | 2.95 | 6.15 |
| HCAE, 330 K | 4.32 | 7.02 |

**Table S6** Ionic conductivity (*σ*, mS cm^–1^), Haven ratio (*H_R_*), and cation transference number (*t*_+_) of HCAE at interfaces obtained from FPMD simulations. *H_R_* and *t*_+_ are dimensionless

| *T* (K) | Region | $\sigma_{\text{GK}}$ | $\sigma_{\text{NE}}$ | $\sigma_{+}^{\text{s}}$ | $\sigma_{-}^{\text{s}}$ | $\sigma_{++}^{\text{d}}$ | $\sigma_{--}^{\text{d}}$ | $\sigma_{+-}^{\text{d}}$ | *H_R_* | *t_+_* |
| --- | --- | --- | --- | --- | --- | --- | --- | --- | --- | --- |
| 270 | I | 100.85 | 160.24 | 47.49 | 112.75 | 719.39 | 953.66 | 866.22 | 1.59 | –0.99 |
|  | II | 134.08 | 181.80 | 52.73 | 129.07 | 757.19 | 931.41 | 868.16 | 1.36 | –0.43 |
|  | III | 152.64 | 200.91 | 57.59 | 143.32 | 795.54 | 912.41 | 878.11 | 1.32 | –0.16 |
| 300 | I | 117.62 | 179.78 | 62.41 | 117.37 | 512.22 | 712.36 | 643.37 | 1.53 | –0.58 |
|  | II | 158.38 | 205.83 | 65.49 | 140.34 | 541.14 | 701.99 | 645.29 | 1.30 | –0.24 |
|  | III | 194.31 | 233.26 | 71.70 | 161.56 | 601.11 | 691.54 | 665.80 | 1.20 | 0.04 |
| 330 | I | 202.44 | 245.03 | 91.32 | 153.71 | 451.25 | 596.68 | 545.26 | 1.21 | –0.01 |
|  | II | 231.30 | 269.46 | 98.42 | 171.04 | 470.51 | 587.59 | 548.13 | 1.17 | 0.09 |
|  | III | 253.34 | 287.95 | 107.37 | 180.58 | 485.14 | 580.55 | 550.15 | 1.14 | 0.17 |

**Table S7** Cation diffusivity (*D*_+_, 10^–10^ m^2^ s^–1^) and anion diffusivity (*D*_–_, 10^–10^ m^2^ s^–1^) of HCAE at interfaces obtained from FPMD simulations

| *T* (K) | Region | *D*_+_ | *D*_–_ |
| --- | --- | --- | --- |
| 270 | I | 1.75 | 4.14 |
|  | II | 1.94 | 4.74 |
|  | III | 2.12 | 5.27 |
| 300 | I | 2.29 | 4.31 |
|  | II | 2.41 | 5.16 |
|  | III | 2.64 | 5.94 |
| 330 | I | 3.36 | 5.65 |
|  | II | 3.62 | 6.29 |
|  | III | 3.95 | 6.64 |

## Description of Supplementary Videos

**Video S1** 24-ps FPMD trajectories of bulk MCAE and HCAE systems. Na^+^ ion is in cyan. O and N atoms in NO_3_^–^ ion are in red and blue, respectively. Water–water H-bond (HB_w-w_) and anion–water H-bond (HB_A-w_) are in red and light blue, respectively. Water molecules not solvated by any ion (H_2_O_F_), solely solvated by cation (H_2_O_C_), solely solvated by anion (H_2_O_A_), and co-solvated by cation and anion (H_2_O_CA_) are in red, cyan, light blue, and grey, respectively.

According to Video S1, HB_w-w_ and H_2_O_F_ are dominant in MCAE, whereas HB_A-w_ and H_2_O_CA_ are prevalent in HCAE. Meanwhile, the events of H-bond breaking and formation are observed between adjacent snapshots, and the occurrence states of water molecules in bulk MCAE and HCAE systems undergo transient transitions among H_2_O_F_, H_2_O_C_, H_2_O_A_, and H_2_O_CA_. All these phenomena suggest the dynamic microstructures in bulk electrolytes. Statistical results related to such bulk microstructures are presented in the main text.

**Video S2** 40-ps FPMD trajectory of the graphene–HCAE interface. C atom is in brown. Na^+^ ion is in cyan. O and N atoms in NO_3_^–^ ion are in red and blue, respectively. HB_w-w_ and HB_A-w_ are in red and light blue, respectively. Water molecules H_2_O_F_, H_2_O_C_, H_2_O_A_, and H_2_O_CA_ are in red, cyan, light blue, and grey, respectively.

According to Video S2, the events of H-bond breaking and formation are observed between adjacent snapshots, and the occurrence states of water molecules in bulk MCAE and HCAE systems undergo transient transitions among H_2_O_F_, H_2_O_C_, H_2_O_A_, and H_2_O_CA_. All these phenomena suggest the dynamic microstructures in HCAE at interfaces. Statistical results related to such interfacial microstructures are presented in the main text.

## Supplementary References

1. K.S. Han, J.D. Bazak, Y. Chen, T.R. Graham, N.M. Washton et al., Pulsed field gradient nuclear magnetic resonance and diffusion analysis in battery research. Chem. Mater. **33**(22), 8562–8590 (2021). <https://doi.org/10.1021/acs.chemmater.1c02891>
2. J. VandeVondele, M. Krack, F. Mohamed, M. Parrinello, T. Chassaing et al., Quickstep: Fast and accurate density functional calculations using a mixed Gaussian and plane waves approach. Comput. Phys. Commun. **167**(2), 103–128 (2005). <https://doi.org/10.1016/j.cpc.2004.12.014>
3. J. Hutter, M. Iannuzzi, F. Schiffmann, J. VandeVondele, cp2k: atomistic simulations of condensed matter systems. Wires Comput. Mol. Sci. **4**(1), 15–25 (2014). <https://doi.org/10.1002/wcms.1159>
4. G. Lippert, J. Hutter, M. Parrinello, A hybrid Gaussian and plane wave density functional scheme. Mol. Phys. **92**(3), 477–487 (1997). <https://doi.org/10.1080/00268979709482119>
5. J. VandeVondele, J. Hutter, Gaussian basis sets for accurate calculations on molecular systems in gas and condensed phases. J. Chem. Phys. **127**(11), 114105 (2007). <https://doi.org/10.1063/1.2770708>
6. S. Goedecker, M. Teter, J. Hutter, Separable dual-space Gaussian pseudopotentials. Phys. Rev. B **54**(3), 1703–1710 (1996). <https://doi.org/10.1103/physrevb.54.1703>
7. C. Hartwigsen, S. Goedecker, J. Hutter, Relativistic separable dual-space Gaussian pseudopotentials from H to Rn. Phys. Rev. B **58**(7), 3641–3662 (1998). <https://doi.org/10.1103/physrevb.58.3641>
8. J.P. Perdew, K. Burke, M. Ernzerhof, Generalized gradient approximation made simple. Phys. Rev. Lett. **77**(18), 3865–3868 (1996). <https://doi.org/10.1103/physrevlett.77.3865>
9. S. Grimme, J. Antony, S. Ehrlich, H. Krieg, A consistent and accurate *ab initio* parametrization of density functional dispersion correction (DFT-D) for the 94 elements H-Pu. J. Chem. Phys. **132**(15), 154104 (2010). <https://doi.org/10.1063/1.3382344>
10. S. Grimme, S. Ehrlich, L. Goerigk, Effect of the damping function in dispersion corrected density functional theory. J. Comput. Chem. **32**(7), 1456–1465 (2011). <https://doi.org/10.1002/jcc.21759>
11. J. VandeVondele, J. Hutter, An efficient orbital transformation method for electronic structure calculations. J. Chem. Phys. **118**(10), 4365–4369 (2003). <https://doi.org/10.1063/1.1543154>
12. T.D. Kühne, M. Krack, F.R. Mohamed, M. Parrinello, Efficient and accurate Car-Parrinello-like approach to Born-Oppenheimer molecular dynamics. Phys. Rev. Lett. **98**(6), 066401 (2007). https://doi.org/10.1103/PhysRevLett.98.066401
13. J. Kolafa, Time-reversible always stable predictor-corrector method for molecular dynamics of polarizable molecules. J. Comput. Chem. **25**(3), 335–342 (2004). <https://doi.org/10.1002/jcc.10385>
14. J. Lan, J. Hutter, M. Iannuzzi, First-principles simulations of an aqueous CO/Pt(111) interface. J. Phys. Chem. C **122**(42), 24068–24076 (2018). <https://doi.org/10.1021/acs.jpcc.8b05933>
15. T. Lu, A comprehensive electron wavefunction analysis toolbox for chemists, Multiwfn. J. Chem. Phys. **161**(8). 082503 (2024). https://doi.org/10.1063/5.0216272
16. M. Brehm, B. Kirchner, TRAVIS - a free analyzer and visualizer for Monte Carlo and molecular dynamics trajectories. J. Chem. Inf. Model. **51**(8), 2007–2023 (2011). <https://doi.org/10.1021/ci200217w>
17. M. Brehm, M. Thomas, S. Gehrke, B. Kirchner, TRAVIS-a free analyzer for trajectories from molecular simulation. J. Chem. Phys. **152**(16), 164105 (2020). <https://doi.org/10.1063/5.0005078>
18. W. Humphrey, A. Dalke, K. Schulten, VMD: Visual molecular dynamics. J. Mol. Graph. **14**(1), 33–38 (1996). <https://doi.org/10.1016/0263-7855(96)00018-5>
19. Q. Wang, Z. Qu, D. Tian, Electric double layer theory of interfacial ionic liquids for capturing ion hierarchical aggregation and anisotropic dynamics. Adv. Energy Mater. **15**(13), 2402974 (2025). <https://doi.org/10.1002/aenm.202402974>
20. J. Guo, Y. Ma, K. Zhao, Y. Wang, B. Yang et al., High-performance and ultra-stable aqueous supercapacitors based on a green and low-cost water-In-salt electrolyte. ChemElectroChem **6**(21), 5433–5438 (2019). <https://doi.org/10.1002/celc.201901591>
